# Supplementary material for: A Comparative Genomic and Phylogenetic Investigation of the Xenobiotic Metabolism Enzymes of Cytochrome P450 in Elephants Shows Loss in CYP2E and CYP4A
Source: Animals (Basel). 2023 Jun 9;13(12):1939. doi: 10.3390/ani13121939 (PMC10294912; doi:10.3390/ani13121939)
Supplement: Supplementary file 1 [file animals-13-01939-s001.zip › CYP seq file/CYP2A.txt]

>Bos taurus CYP2A13 XM 024979093------------------------ATGCTGGCCTCAGGGCTGCTCCTCGTGGCTTTGCTGGCCTGTCTGACTATCATGGTCTTGATGTCTGTCTGGCGGCAAAGGAACCTCAAAGGGAAATTGCCTCCAGGGCCCACTCCTCTGCCCTTCATCGGGAACTACCTGCAGCTGAACACCGAGCAGATGTGCAACTCCCTCATGAAGATCAGCGAGCACTACGGCCCTGTGTTCACGGTCCACCTGGGGACCCGGCAGATTGTGGTGCTGTGTGGCTATGATGCTGTGAAGGAGGCTCTGGTGGACCAGGCTGAAGAATTCAGTGGGCGAGGCAAGCAGGCTACCTTTGACTGGCTCTTCAAAGGCTATGGCGTGGCGTTCAGCAACGGGGAGCGCGCCAAGCAGCTCCGGCGCTTCTCGATTACAACTCTGCGGGACTTCGGCGTGGGCAAGCGCGGCATCGAAGAGCGCATCCAGGAGGAGGCGGGCTTCCTCATCGAGGCCTTCCGGGGCACTCGCGGC------GCCTTCATCGATCCCACCTTCTTCCTGAGCCGAACGGTCTCCAATGTCATCAGCTCCATTGTCTTCGGGGACCGCTTTGACTACGAGGACAAAGAGTTCTTGTCACTACTGCGAATGATGCTGGGAAGCTTCCAGTTCACCGCTACGTCTACCGGGCAGCTCTACGAGATGTTCTACTCAGTGATGAAATATCTGCCAGGGCCACAGCAACAGGCCTTTAAGGAGCTGCAGGGACTGGAGGACTTCATAGCCAAGAAGGTGGAACAAAACCAGCGCACGCTGGACCCCAACTCCCCACGGGACTTCATCGACTCCTTCCTCATCCGCATGCAGGAGGAGAAGGAGAATCCCAACACGGAGTTCTACAGGAAGAACCTGGTGATGACAACACTGAACCTCTTCTTTGCGGGCACCGAGACGGTCAGCACGACCATGCGGTATGGCTTCCTGCTGCTCATGAAGCACCCAGATGTGGAGGCCAAAATCCATGAGGAGATTGACCGCGTGATCGGCAAGAACCGTCAGCCCAAGTTTGAGGACCGAGCCAAGATGCCCTACACAGAGGCTGTGATCCACGAGATCCAGAGATTCGGAGACATGATCCCCATGGGCTTGGCTCGCAGAGTCACCAAGGATACCAAGTTTCGAGACTTCCTGCTCCCCAAGGGCACCGAAGTGTTCCCTATGCTGGGCTCTGTGCTGAGAGACCCCAAGTTCTTCTCCAACCCCCGAGATTTCAATCCCCAGCACTTCCTGGACGAGAAGGGGCAATTTAAGAAGAGTGATGCTTTTGTGCCCTTCTCCATCGGAAAGCGGTACTGTTTCGGAGAAGGCCTCGCCAGAATGGAGCTCTTCCTCTTCTTCACCACCATCATGCAGAACTTCCGCTTCAAGTCCCCACAATCGCCCCAGGACATCAACGTGTCCCCCAAACTCGTGGGCTTTGCCACTATCCCTCCAAACTACACCATGAGCTTCCTGCCCCGTTGA------------>Canis lupus familiaris CYP2A13 NM_001037345------------------------ATGTTGGCCTCAGGGCTTCTTCTGGTGGCTTTGCTGGCCTGCCTGACAATAATTGTCTTGATGTCTGTCTGGAAGCAGAGGAAACTGGGGGGGAAGCTCCCTCCAGGACCCACCCCACTGCCCTTCATCGGAAATTACCTGCAGCTGAACACAGAGCAGATGTACAACTCTCTCATGAAGATCAGCGAGCGCTATGGCCCGGTGTTCACCATCCATCTGGGGCCCCGGCCTGTCGTGGTGCTGTGTGGACACGAGGCGGTGAAGGAGGCTCTGGTGGACCAGGCGGAGGAATTCAGCGGGCGAGGCGAGCAGGCCACATTTGACTGGCTCTTCAAAGGCTATGGCGTGGCGTTCAGCAATGGGGAGCGGGCCAAGCAGCTCAGGCGCTTCTCCATCACCACACTGCGGGACTTTGGAGTGGGAAAGCGCGGCATTGAAGAGCGCATTCAGGAGGAGGCAGGCTTCCTCATTGAGGCCCTCCGGGGCACACGAGGT------GCCTTCATCGATCCCACCTTCTTCCTGAGCCGAACAGTGTCCAATGTCATCAGCTCCATTGTTTTTGGGGATCGCTTTGACTATGAGGACAAAGAGTTCCTGTCACTGCTGCGTATGATGCTGGGGAGCTTCCAGTTCACAGCTACATCTATGGGGCAGCTCTATGAGATGTTTTACTCAGTGATGAAACACCTGCCAGGGCCACAGCAACAGGCATTTAAGGAGCTGCAGGGTCTGGAAGACTTCATAACCAAGAAAGTGGAGCAGAACCAACGCACACTGGATCCCAACTCCCCTCGAGACTTCATTGACTCCTTCCTTATCCGCATGCAGGAGGAGCAGAACAACCCTAACACGGAGTTTTACTTGAAGAACCTGGTGTTGACCACACTGAACCTCTTCTTTGCGGGCACTGAGACAGTCAGTACAACCCTGCGGTATGGCTTCCTGCTGCTCATGAAACACCCAGATGTGGAGGCCAAAGTCCATGAGGAGATTGATCGGGTGATTGGCAAGAACCGTCAGCCCAAGTTTGAGGACAGGGCCAAGATGCCCTACACAGAGGCAGTGATCCATGAGATCCAAAGATTTGGAGACATGATCCCCATGGGCGTGGCCCGCAGAGTCATCAAGGACACCAAGTTTCGGGAGTTTCTCCTCCCCAAGGGCACTGAAGTGTTCCCTATGCTGGGCTCTGTGCTGAGAGATGCCAAGTTCTTCTCCAACCCCCAAGACTTCCACCCTCAGCACTTCCTGGATGAGAAGGGGCAGTTTAAGAAGAGTGATGCTTTTGTGCCCTTCTCCATTGGAAAGCGATACTGTTTTGGAGAAGGCCTGGCTAGGATGGAGCTCTTTCTCTTCCTCACCACCATCTTGCAGAACTTCCACTTCAAGTCCCCGCAGCTGCCTCAAGACATCGATGTGTCCCCCAAACATGTGGGTTTTGCGACCATCCCACGAAATTACACCATGAGCTTCCAGCCCCGCTGA------------>Canis lupus familiaris CYP2A7 NM_001048027------------------------ATGGTGGCCTCAGGGATCCTTCTGGTGGCTTTGCTCACCTGCTTGACAGTAATGGTCTTGATGTCTGTCAGGAGGCAGTGGAAGCTCTTGGAGAAGCTCCCTCCAGGACCTACTCCATTGCCCTTCATCGGGAACTATCTGCAGCTGAACATACAGCAGATGTCTGATTCCTTCATGAAGATCAGCAAACGCTATGGCCCTGTGTTCACCATCCACCTGGGGCCTAGGCGCGTCGTGGTACTGTGTGGATACGAGGCGGTGAAGGAAGCTCTGGTGGACCAGGCTGAAGAATTCAGTGGGCGAGGCGCACAGGCCACCTTTGACACTCTCTTCAAAGGCTATGGGGTGACGTTTAGCAATGGGGAGCGGGCCAAGCAGCTCAGGCGCTTCTCCATCACCACCCTGCGGGACTTTGGAGTGGGAAAGCGCGGCATTGAAGAGCGCATTCAGGAGGAGGCAGGCTTCCTCATTGAGGCCCTCCAGGGCACACGAGGT------GCCTTCATCGATCCCACCTTCTTCCTGAGCCGAACAGTGTCCAATGTCATCAGCTCCATTGTTTTTGGGGATCGCTTTGACTATGAGGACAAAGAGTTCCTGTCACTGCTGCGTATGATGCTGGGGAGCTTCCAGTTCACAGCTACATCTATGGGGCAGCTCTGTGAAATGTTCCATTCAGTGATAAAATACCTGCCAGGGCCACAGCAACAGGCAATTAAGGAGCTGCAGGGTCTGGAAGACTTCATAACCAAGAAGGTGGAGCAGAACCAACGCACACTAGACCCCAACTCTCCTCGAGACTTCATTGACTCCTTCCTCATCCGCATGCAGGAGGAGCAGAACAACCCCAACACGGAGTTTCACTTGAAGAACCTGGTGTTGACCACACTGAACCTCTTCTTTGCGGGCACTGAGACAGTCAGTACAACTCTGCGGTATGGCTTCCTGCTGCTCATGAAGCACCCAGATGTGGAGGCCAAAGTCCATGAGGAGATTGATCGGGTGATTGGTAAGAACCGTCAGCCCAAGTTTGAGGACAGGGCCAAGATGCCCTACACAGAGGCAGTGATCCATGAGATCCAAAGATTTGGAGACATAATCCCTTTGAGTCTGGCCCGCAGAGTCATCAAGGACACCAAGTTTCGGGAGTTTCTCCTCCCCAAGGGCACTGAAGTGTTCCCTATGCTGGGCTCTGTGCTGAGAGATGCCAAGTTCTTCTCCAACCCCCAAGACTTCCACCCTCAGCACTTCCTGGATGAGAAGGGGCAGTTTAAGAAGAGTGATGCTTTTGTGCCCTTCTCCATTGGAAAGCGATACTGTTTTGGAGAAGGCCTGGCTAGGATGGAGCTCTTTCTCTTCCTCACCACCATCTTGCAGAACTTCCACTTCAAGTCCCCGCAGCTGCCCCAAGACATCGACGTGTCCCCCAAGCTCGTGGGCTTAGCCACCATCCCACGAAATTACACCATGAGCTTCCAGCCCCGCTGA------------>Equus caballus CYP2A13-like XM 023649781------------------------ATGCTGGCCTCAGGGCTGCTTCTGGTGGCTTTGCTGGCCTGCCTGGTTTTAATGGTTTTCATGTCAGTCTGGTGGCAGAGGGAGCTCTGGGGAAAGCTGCCTCCTGGACCCACCCCATTGCCCTTCATCAGGAACTACCTGCAGCTGGACACAGAGCAGATATACGACTCCCTCATGAAGATCAGCAAGCGCTATGGGCCAGTGTGCATGGTTCACCTGGGGCCCCGGAGGGTCGTGGTGCTGCGTGGATACGAAGCTGTAAAGGAGGCTCTGCTAGACCAGGCAGAGGAATTCAGTGGACGAGGAGAGCTGGCCGTTGCTGACCGGATCTTCAAAGGCTATGGCGTGGCATTCAGCAACGGGGAGCGGGCCAAGCAGCTCAGGCGCTTCTCCATCACCACACTGAGGGACTTCGGAGTGGGCAAGCGTGGCATTGAGGAGCGCATCCAGGAGGAGGCGGGCTTCCTCATTGAGGCCTTCCAGAGCATGCGTGGC------AACTTCATTGATCCCACCTTCTTCCTGAGCCAAGCTGTCTCCAATGTCATCAGCTCCATTGTCTTTGGGAACCGCTTTGACTATGAGGACAAAGAGTTCCTGTCACTGCTGCGTATGATGCACGGAAGCTTCCAGTTCACANCTACATCTACGGGGCAGCTCTATGAGATGTTCTACTCAGTGATGAAACACCTGCCAGGGCCACAGCAACAGGCCTTTAAGGAGCTGCAGGGCCTGGAGGACTTCATAACCAAGAAGGTGGAGCAGAACCAACGCACCCTGGATCCCAACTCCCCGCGGAACTTCATCGACTCCTTCCTCATCCGCATGCAGCAGGAGCAGAAGAACCCCAACACGGAGTTCTGCTTGAAGAACCTGGTGCTGACCACGTTGAACCTCTTCATTGGTGGCACAGAGACGGTCAGCATAACCCTGCGTTATGGCTTCCTGCTGCTGATGAAGCACCCAGATATAGAGGCCAAGGTTCATGAGGAGATTGATCGAGTGATTGGCAAGAACCGTCAGCCCAAGTTTGAGGACCGGGCCAAGATGCCCTACACAGAGGCAGTGATCCATGAAATCCAAAGATTTGCAGATGTGGCCCCCATGAGCCTGGCCCGTAGAGTTATCAAGGATACCAAGTTTCGGGGCTTCCTCATCCCCAAGGGCACTGAAGTGTTCCCTATGCTGGGCTCCGTCCTGAGAGACCCCAAGTTCTTCTCCAATCCCCACGATTTCAACCCCCAGCACTTCCTGGATGAGAAGGGGCAGTTTAAGAAGAGTGATGCTTTTGTGCCTTTCTCCATTGGAAAGCGGCACTGCTTTGGAGAAGGCCTGGCTAGAATGGAGCTCTTTCTCTTCCTCACCACCATCATGCAGAATTTCTGCTTCAAGCACTCTCAGTCGCCCCAGGATATCGACGTGTCCCCGAAACACGTGGGCTTTGCCAGCATTCCAAGAAACTACACCATGAGCCTCCAGCCCCGCTAA------------>Equus caballus CYP2A13 NM 001111337 XM 001499763------------------------ATGCTGGCCTCAGGGCTGCTTCTGGTGGCTTTGCTGTCCTGCCTGACTTTAATGGTATTGATGTCTGTCTGGCGGCAGAGAAAGCTCTGGGGAAGGCTGCCTCCTGGACCCACCCCATTGCCCTTCATCGGGAACTACCTGCAGCTGGACACAGAGCGGATATGTGACTCCCTCATGAAGATAGGCAAGCGCTATGGGCCAGTGTTCACAGTTCACCTGGGGCCCCGCCGGGTCGTGGTGCTATGTGGATACGATGCTGTGAAGGAGGCTCTGTTGGACCAGGCTGAGGAATTCAGTGGACGAGGAGAGCAGGCCACCTTCAACTGGATCTTCAAAGGCTATGGTGTGGCATTCAGCAATGGGGAGCGGGCCAAGCAGCTCAGGCGCTTCTCCATCACCACACTGAGGGACTTCGGAGTGGGTAAGCGTGGCATTGAGGAGCGCATCCAGGAGGAGGCGGGCTTCCTCATCGAGGCCTTCCGGAGCACGTGTGGC------AACTTCATTGATCCCACCTTCTTCCTGAGCCAAGCTGTCTCCAATGTCATCAGCTCCATTGTCTTTGGGAACCGCTTTGACTATGAGGACAAAGAGTTCCTGTCACTGCTGCGTATGATGCTGGGAAACTTCCAGTTCACAGCTACATCTACGGGGCAGCTCTATGAGATGTTCTACTCAGTGATGAAACACCTGCCAGGGCCACAGCAACAGGCCTTTAAGGAGCTGCAGGGCCTGGAGGACTTCATAGCCAAGAAGGTGGAGCAGAACCAACGCACCCTGGATCCCAACTCCCCGCGGAACTTCATCGACTCCTTCCTCATCCGCATGCAGCAGGAACAGACGAACCCTAACACGGAGTTCTACTTGAAGAACCTGGTGATAACCACGTTGAACCTCTTCATTGCTGGCACAGAAACGATCAGCACAACCCTGCGTTATGGCTTCCTGCTGCTGATGAAGCACCCAGATATAGAAGCCAAGGTTCATGAGGAGATTGATCGAGTGATTGGCAAGAACCGTCAGCCCAAGTTTGAGGACCGGGCCAAGATGCCCTACACAGAGGCAGTGATCCACGAAATCCAAAGATTTGGAGATGTGATCCCCATGAGCCTGGCCCGTAGAGTTACCAAGGATACCAAGTTTCGGGGCTTCCTCATCCCCAAGGGCACTGAAGTGTTCCCTATGCTGGGCTCCGTCCTGAGAGACCCCAAGTTCTTCTCCAATCCCCACGATTTCAACCCCCAGCACTTCCTGGATGAGAAGGGGCAGTTTAAGAAGAGTGATGCTTTTGTGCCTTTCTCCATTGGTAAGCGGTACTGCTTTGGAGAAGGCCTGGCTAGAATGGAGCTCTTTCTCTTCCTCACCACCATCATGCAGAATTTCTGCTTCAGGTACCCTCAGTCGCCCCAGGATATCGACGTGTCCCCGAAACACGTGGGCTTTGCCAGCATTCCAAGAAACTACACCATGAGCCTCCAGCCCCGCTAA------------>Equus caballus CYP2A13 XM 001916316------------------------ATGCTGGCCTCAGGGCTGCTTCTGGTGGCTTTTCTGGCCTGCCTGACCTTAATGGTCTTGATGTCTGTCTGGCGGCAGGGGAAGTTCTTGGGGAAGCTGCCTCCTGGACCCACCCCATTGCCTTTCATTGGGAACTACCTGCAGCTGAACACAGAGCAGATGTACAACTCCCTCATGAAGATCAGCAAGCGCTATGGGCCAGTGTTCACGGTTCACCTGGGGCCCCGGCGAATCGTGGTGCTATGTGGATACGAAGCTGTGAAGGAGGCTCTGTTGGACCAGGCTGAGGAATTCAGTGGACGAGGAGAGCAGGCCACCTTCGACTGGCTCTTCAAAGGCTATGGTGTGGCATTCAGCAACGGCGAGCGTGCCAAACAGCTCAGACGCTTCTCCATCACTACACTGCGGGACTTCGGAGTGGGCAAGCGTGGCATTGAGGAACGCATTCAGGAGGAAGCAGGCTTCCTCATCGAGGCCTTCCGGAGCACACGTGGT------ACCTTGATTGATCCCACCTTCTTCCTGAGCCGAGCTGTCTCCAATGTCATCAGCTCCATTGTCTTTGGGGATCGCTTTGACTATGAAGACAAAGAGTTCCTGTCACTGCTGCGTATGATGCTGGGAAGCTTCCAATTTACAGCTACGTCTACGGGGCAGCTCTATGAGATGTTCTACTCAGTGATGAAACACCTGCCAGGGCCACAGCAACAGGCCTTTAAGGAGCTGCAGGGCCTGGAGGACTTCATAGCCAAGAAGGTGGAGCAGAACCAACGCACCCTGGATCCCAACTCCCCGCGGAACTTCATCGACTCCTTCCTCATCCGCATGCAGCAGGAGCAGAAGAACCCCAACACAGAGTTCTACTTGAAGAACCTGGTGCTGACCACGTTGAATGTCTTCTTTGCTGGCACAGAGACAGTCAGCACAACACTACGCTATGGCTTCCTGCTGCTCATGAAGCACCCAGATGTGGAGGCCAAGGTCCATGAGGAGGTTGACCGGGTGATTGGCAAGAACCGTCAGCCCAAGTTTGAGGACCGGGCCAAAATGCCCTACACAGAGGCGGTGATTCACGAGATCCAAAGATTTGGAGACATGATCCCAATGGGCGTGGCCCGCAGAGTCACCAAGGACACCAAATTTCGAGACTTTCTCCTCCCAAAGGGCACTGAAGTGTTCCCTATGCTGGGCTCTGTCCTGAGAGACCCCAGGTTCTTCTCCAATCCCCACGATTTCAACCCCCAGCACTTCCTGGATGAGAAGGGGCAGTTTAAGAAGAGTGATGGGTTCGTGCCCTTCTCCATTGGAAAGCGGTACTGTTTTGGAGAAGGCCTGGCTAGGATGGAGCTCTTTCTATTCCTCACTACCATCATGCAGAACTTCCACTTCAAATCCCCGCAATCACCTCAAGACATCGACGTGTCCCCCAAACATGTGGGCTTTGCCACTATCCCACGAAACTACACCATGAGCTTCCAGCCCCGCTAG------------>Elephantulus edwardii CYP2A13 XM 006901251 LOC102856980------------------------ATGCTGGCCTCAGGGCTGCTGCTGGTGGCTCTGCTAGCCTGCCTGTCTGTAATGATCCTGATGTCTGTCTGGCGACAAAGGAAGACGTGGGGGAAGCTCCCCCCGGGGCCCACTCCACTGCCCTTCATCGGGAACTACCTACAGCTGAACACAGAGCAGATGTACAATTCCCTCATGAAGCTCAGAGAGCACTATGGTCCTGTGTTCACCATCCACCTGGGGACCCGGCGGATCGTGGTGCTGTGCGGATATGATGCTGTAAAAGAGGCTCTGGTGGACCAGGCTGAAGAATTCAGTGGGCGAGGCGAACAAGCCACCTTCGACTGGCTCTTCAAAGGCTATGGCGTGGCGTTCAGCAACGGCGAGCGCGCCAAGCAGCTGCGGCGCTTCTCCATCACCACCCTGCGGGACTTCGGCGTGGGCAAGCGAGGCATCGAGGAGCGCATCCAGGAGGAGGCGGGCTTTCTCATCGAGACCTTCCGGAACACACGCGGA------GCCTTCATCGACCCCACCTACTTCCTGAGCCGCACTGTGTCCAATGTCATCAGCTCCATCGTCTTTGGGGACCGCTTTGACTATGAGGACAAAGAGTTCCTGTCACTCCTGCGTATGATGCTGGGAAGCTTCCAGTTCACAGCTACTTCCACCGGACAGCTCTATGAGATGTTTGCCCCAGTGATGAAACATCTCCCAGGGCCACAGCAACAGGCCTTCAAGGAGCTGCAGGGACTCGAGGACTTCATAGTTAAGAAGGTGGAGCAGAACCAGCGCACACTGGACCCCAACTCCCCACGGGACTTCATTGACTCCTTCCTCATCCGCATGCGAGAGGAGAAGAAGAACCCCAACACGGAGTTCTACATGAAGAACCTGGTGCTGACCACGCTGAACCTCTTCTTTGCGGGCACTGAGACCGTCAGCACCACCTTGCGTTACGGCTTCCTGCTGCTCATGAGGCATCCAGAAGTGGAGGCCAAGCTGCATGAAGAGATTGACCGGGTGATTGGCAAGAACCGTCAGCCCAAGTTTGAGGACCGGGCCAAGATGCCCTACATGGAGGCAGTGATCCACGAGATACAAAGATTTGGAGACATGATCCCCATGGGCTTGTCCCGCAGGGTCACCAAGGACACCAAATTCCGGGACTTCCTCATCCCTAAGGGCACTGAAGTATTCCCCGTGCTGGGTTCTGTGCTAAGAGACACCAAGTTCTACTCCCATCCCAACGATTTCCACCCCCAGCACTTCCTGGATGAGAAAGGGCAATTCAAGAAGAGCGATGCCTTTGTGCCCTTCTCCATCGGCAAGCGGTACTGTTTTGGCGAAGGCTTGGCCAGAATGGAGCTCTTCCTCTTCCTTACCACCATCCTGCAGAACTTCTCCTTCAAGTCCCCGCAGTCTCCCAAGGACATCGATGTGTCCCCAAAACATGTGGGCTTTGCCACCATCCCACGCACCTACACCATGAGCTTCCAACCCCGCTGA------------>Elephas maximus CYP2A13 XM 049900690 LOC126085373------------------------ATGCTGGCCTCAGGGATGCTTCTGGTGGCTTTGCTGGCCTGCCTGTCTGTAATGGTCTTGATGTCTGTCTGGCGACAAAGGAAGCTCTGGGGGAAGCTTCCCCCTGGTCCCACTCCCTTGCCTTTCATCGGGAACTACCTGCAGCTGAACACACAGCAGATGTACAACTCCCTCATGAAGCTCAGTGAGCGCTATGGCTTGGTGTTCACGGTCCACTTGGGGTCCCGGCGGGTTGTGGTACTGTGCGGATACGACGCTGTGAAGGAGGCTCTGGTGGACCAGGCTGAGGAATTCAGTGGGCGAGGCGAGCAGGCCACCTTCGACTGGCTCTTCAAAGGCTACGGAGTGGCGTTCAGCAACGGGGAGCGAGCCAAGCAGCTCCGGCGCTTCTCCATCACCACGCTGCGGGACTTCGGCGTGGGAAAGCGTGGCATTGAGGAGCGTATCCAGGAGGAGGCCGGCTTCCTCATCGAGACCTTCCGGGGCACGCGCGGC------ACCTTCATTGATCCCACCTACTTCCTGAGCCGAACTGTCTCCAATGTCATCAGCTCCATCGTCTTCGGAGACCGGTTTGACTATGAAGATAAAGAGTTCCTTTCACTGCTGCGTATGATGCTGGGAAGCTTCCAGTTCACAGCTACCGCTACCGGACAGCTCTATGACATGTTCTACTCGGTAATGAAATACCTACCAGGGCCACAGCAACAGGCCTTTAAGGAGCTGCAGGGGCTGGAGGACTTCATAACCAAGAAGGTGGAGCAGAACCAGCGCACACTGGACCCCAACTCCCCACGGGACTTCATTGACTCCTTCCTCATCCGCATGCGGGAGGAGAAGAAGAACCCCAACACAGAGTTCTACATGAAGAACCTGGTGCTGACCACGCTAAACCTCTTCTTTGCGGGCACAGAGACCGTCAGCACAACCCTGCGTTACGGCTTCCTACTGCTCATGAAGCACCCAGATGTGCAGGCCAAGTTGCACGAGGAGATTGACCGGGTGATTGGCAAGAACCGTCAGCCCAAGTTTGAGGACCGGGCCAAGATGCCCTACACCGAGGCTGTGATCCATGAGATCCAGAGATTTGCAGACATGATCCCCATGGGGGTGGCCCGCAGGGTCACCAAGGACACCAAGTTTCGGAACTTCTTCATCCCCAAGGGCACAGAAGTGTTCCCTATGCTGGGCTCTGTGCTGAGAGACACCAAGTTCTTCTCCAACCCCCAAGATTTCAACCCCCGGCACTTCCTGGATGAGAAAGGGCAATTTAAGAAGAACGACGCCTTTGTGCCCTTCTCCATTGGAAAGCGGTACTGTTTTGGGGAAGGTCTGGCCAGAATGGAGCTCTTTGTCTTCCTTACCACCATCTTGCAGAACTTCTGCTTCAAGTCCCCGCAGTCGTCCAAGGACATCGATGTGTCTCCCAAACACGTGGGCTTTGCCACTATCCCACGAACCTACACCATGAGCTTCTTGCCCCGCTGA------------>Elephas maximus CYP2A13 XM 049900691 LOC126085374------------------------ATGCTGGCCTCAGGGATTCTTCTGGTGGCTTTGCTGGCCTGCCTGTCTGTAATGGTCTTGATGTCTGTCGGGCGACAAAGGAAGCTCTGGGGGAAGCTTCCTCCTGGGCCCACTCCCTTGCCTTTCATCGGGAACTACCTGCAGCTGAACACACAGCACATGTACAACTCCCTGAAGAAGCTCAGTGAGCGCTATGGTTCGGTGTTCACGGTCCACCTGGGGCCCCGGCGGGTTGTGGTACTGTGGGGATTCGATGCTGTGAAGGAGGCTCTGGTGGACCAGGCTGAGGAATTCAGCGGACGAGGCGAGCAGGCCACCTTCAGCGAGATCTTCAAAGGCTACGGAGTGGCGTTCAGCAACGGGGAGCGCGCCAAGCAGCTCCGGCGCTTCTCCATCACCACGCTGCGGGACTTCGGCGTGGGAAAGCGCGGCATTGAGGAGCGCATCCAGGAGGAGGCTGGCTTCCTCATCGAGGCCTTCCGGGGCACGCGCTGC------GCCTTCATCGATCCCACCTACTTCGGGAGCCGTGCGGTCTCCAACGTCATCAGTTCCATCGTCTTTGGAAACCGCTTTGCCTATGAGGATAAAGAGTTCCTGTCACTGCTGCGTATGATACTGGGAAGCTTCCAGTTCACAGCTACTGCTACCGGACAGCTCTATGACATGTTCTACTCGGTAATGAAACACCTACCAGGGCCACAGAAACAGGCCTTGAAGTTGCTGCACGGGCTGGAGGATTTCATAACCAAGAAGGTGGAGCAGAACCAGCGCACACTGGACCCCAACTCCCCACGGGACTTCATCGACTCCTTCCTCATCCGCATGCAGGAGGAGAAGAAGAACCCCAACACAGAGTTCCATATGAAGAATCTGGTGATGACCACGCTGGCCCTCTTCTTTGCCGGCACAGAAACCATCAGCACGACCCTTCGCTGGGGCTTCCTGCTGCTCATGAAGCACCCAGATGTGCAGGCCAAGGTGCATGAGGAGATTGACCAAGTGATTGGCAAGAGCCGTCAGCCCAAGTTTGAGGACCGGGCCAAGATGCCCTACACCGAGGCAGTGATCCATGAGATCCAGAGATTTACAGACATGATCCCCATGGGGTTGCCCCGCAGGGTCACCAAGGACACCAAGTTTCGGGACTTCTTCATCCCCAAGGGCACAGAAGTGTTCGCTATGCTGGGCTCCGTGCTGAGAGACACCAAGTTCTTCTCCAACCCCCAAGATTTCAACCCCCAGCACTTCCTGGATGAGAAAGGGCAATTTAAGAAGAATGACGCCTTTGTGCCCTTCTCCGTTGGAAAGCGGTACTGTTTCGGGGAAGGTCTGGCCAGAATGGAGCTCTTTGTCTTCCTTACCACCATCTTGCAGAACTTCTGCTTCAAGTCCCCACAGTTGCCCAAGGACATCGATATGTCTCCCAAACATGTGGGCTTTGCCACCATCCCACAAACCTACACCATGAGCTTCTTGCCCCGCTGA------------>Elephas maximus CYP2A13 XM 049900693 LOC126085376------------------------ATGCTGGCCTCAGGGATGCTTCTGGTGGCTTTGCTGACCTGCCTGTCTGTAATGGTCTTGATGTCTGTCTGGCGACAAAGGAAGCTCTGGGGGAAGCTTCCCCCTGGGCCCACTCCATTGCCTTTCATCGGGAACTACCTGCAGCTGAACACAGAGCAGATGTACAACTCCCTGAAGAAGCTCAGTGAGCGCTATGGTTCGGTGTTCACGGTCCACCTGGGGCCCCGACGGGTCGTGGTACTGTGGGGATACGACGCTGTGAAGGAGGCTCTCATGGACCAGGCTGAGGAATTCAGTGGGCGAGGGGAGCAGGCCACCTTCGACTGGCTCTTCAAAGGCTACGGCGTGGCGTTCAGCGAGGGGGAGCGGGCCAAGCAGCTCCGGCGCTTCTCCATCACCACGCTACGGGACTTCGGGGTGGGCAAGCGCGGCATTGAAGAGCGCATCCAGGAGGAGGCCGGCTTCCTCGTGGAGACCTTGCGAGGCACATGCGGC------GCCTTCGTCGATCCCACCTACTTCTTGAGCCGTGCAGTCTCCAACGTCATCAGCTCCATTGCCTTTGGGGACCGTTTTGCCTATGAAGATAAAGAGTTCCTGTCACTGCTGCGTATGATGCTGGGAAGCTTCCAGTTCACAGCTACCGCTACCGGACAGCTCTATGACATGTTCTACTCGGTAATGAAACACCTACCAGGGCCACAGCAACAGGCCTTTAAGGAGCTGCACGGGCTGGAGGATTTCATAACCAAGAAAGTGGAGCAGAACCAGCGCACACTGGACCCCAGCTCCCCACGGGACTTCATCGACTCCTTCCTCATCCGCATGCAGAAGGAGAAGAAGAACCCCAACACAGAGTTCTGCATGAAGAACCTGGTGTTGACCACACTGAACCTCTTCTTTGCCGGCACCGAGACTGTCAGCACGACTCTGCGCTACGGCTTCCTGCTGTTCATGAAGCACCCAGATGTGTGGGCCAAGGTGCAAGAGGAGATTGACCTGGTGATTGGCAAGAACCGTCAGCCCAAGTTTGAGGACCGGGCCAAGATGCCCTACACCGAGGCAGTGATCCACGAGATCCAGAGATTTACAGACATGATCCCCATGGGGGTGGCCCGCAGGGTCACCAAGGACACCAAGTTTCGGGACTTCTTCATCCCCAAGGGCACTGAAGTGTTCCCTGTGCTGGGCTCCGTGCTGAGAGACACCAAGTTCTTCTCCAACCCCCAAGATTTCAACCCCCAGCACTTCCTGGATGAGAAAGGGCAATTTAAGAAGAACGATGCCTTTGTGCCCTTCTCCATTGGAAAGCGGTACTGTTTCGGGGAAGGTCTGGCCAGAATGGAGCTCTTTGTCTTCTTTACCACCATCCTGCAGAACTTCTGCCTCAAGTCCCCTCAGTCGCCCGAGGACATCGATGTGTCTCCCAAACATGTGGGCTTTGCCACCATCCCACGAACCTACACCATGAGCTTCTTACCCCGCTGA------------>Felis catus CYP2A13 XM 003997781------------------------ATGCTGGCAGCAGGGCTCCTTCTGGTGGCTTTGCTCACCTGCCTGACAATAATGGTCTTGATGTCTGCCTGGAGGCAGAGGAAGCTCTGGGGGAAGCTCCCTCCAGGACCCACCCCATTGCCCTTCATCGGGAATTACCTGCAGCTGAACACACAGCAAATGTACAACTCTCTCATGAAGATCAGCGAGCGCTATGGCCCCGTGTTCACGGTCCACCTGGGGCCACGGCGCATTGTGGTGCTGTGTGGACACGAGGCCGTGAAGGAGGCGCTGGTGGACCAGGCTGAGGAATTCAGTGGGCGAGGAGAGCAGGCCACCTTCGACTGGCTCTTCAAAGGCTATGGTGTGGCGTTCAGCAATGGCGAGCGAGCCAAGCAGCTCCGGCGCTTCTCCATCACCACGCTGCGGGACTTTGGTGTGGGCAAGCGCAGCATCGAGGAGCGCATCCAGGAGGAAGTGGGCTTCCTCATCGAGGCCCTCCGGGGCACGCATGGT------GCCTTCATCGATCCCACCTTCTTCCTGAGCCGGACAGTGTCCAATGTCATCAGCTCCATTGTCTTTGGGGACCGCTTTGACTATGAGAACAAAGAGTTCCTGTCACTGCTGCGTATGATGCTGGGAAGCTTCCAGTTCACAGCTACATCTATGGGTCAGCTCTATGAGATGTTTTATCCCGTGATGAAATACCTGCCAGGACCACAGCAACAGGCATTTAAAGATCTGAAAGGTCTGGAGGACTTCATAACCAAGAAGGTGGAGCAGAACCAACGCACGCTAGATCCCAACTCCCCGCGGGACTTCATCGACTCCTTCCTCATCCGCATGCAGGAGGAGCAGAACAACCCCAACACAGAGTTCTACTTGAAGAACCTGGTGCTGACCACACTGAACCTCTTCTTTGCGGGCACTGAGACAGTCAGCACGACCCTGCGGTATGGCTTCCTGCTGCTCATGAAGCACCCAGATGTGGAGGCCAAAATCCACGAGGAGATTGACCAGGTGATTGGCAAGAACCGGCAGCCCAAGTTTGAGGACAGGGCCAAGATGCCCTACACAGAGGCGGTGATCCATGAGATCCAAAGATTTGGAGACATGATCCCCATGGGATTGGCCCGCAGAGTCACCAAGGACACCAAGTTTCGGGAGTTCTTCCTCCCCAAGGGCACTGAAGTGTTCCCTATGCTGGGCTCTGTGCTGAGAGACCCCAAGTTCTTTTCCCACCCCCGAGACTTCCACCCCCAGCACTTCCTGGATGAGAAGGGCCAGTTTAAGAAGAGTGATGCTTTTGTGCCCTTCTCCATCGGAAAGCGGTACTGTTTCGGAGAAGGTCTGGCTAGAATGGAGCTCTTTCTCTTCCTCACCACCATCTTGCAGAACTTCTGCCTCAAGTCCCCTCAGCTGCCCCAAGACATTGACGTGTCCCCCAAAGTTGTGGGCTTTGCCACTATCCCACGAAATTACACCATGAGCTTCCAGCCCCGCTGA------------>Felis catus CYP2A25 XM 011289955------------------------ATGCTGGCCTCAGAGCTCCTTCTGGTGGCTTTGCTCACCTGCCTGACAATGATGGTCTTGATGTCTGCCTGGAGGCAGAGGAAGCTCTGGGGGAAGCTCCCTCCAGGACCCACCCCATTGCCCCTCATTGGGAACTACCTGCAGCTGAACACAAAGCAGCTGTCGGATTCCTTCATGAAGATCAGTGAGCGCTATGGCCCCGTGTTCACGGTCCACCTGGGGCCACGGCGCATTGTGGTGCTGTGTGGACACGAGGCCGTGAAGGAGGCGCTGGTGGACCAGGCTGAGGAATTCAGTGGGCGAGGCGGGCAGGCCACCTTCGACTTACTCTTCAAAGGCTATGGGGTGACGTTCAGCAATGGCGAGCGAGCCAAGCAGCTCCGGCGCTTCTCCATCACCACGCTGCGGGACTTTGGTGTGGGCAAGCGTGGCATCGAGGAGCGGATCCAGGAGGAGGCAGGCTTCCTCATCGAGGCTTTCCAGGGCACGCAAGGC------ACCTTCATCGATCCCACCTTCTTCCTGAGCCGGACAGTGTCCAATGTCATCAGCTCCATTGTCTTTGGGGACCGCTTTGACTATGAGGACAAAGAGTTCCTGTCACTGCTGCGTATGATGCTGGGAAGCTTCCAGTTCACAGCTACATCTATGGGTCAGCTCTGTGAAATGTTCCCATCAGTCATGAAACACCTGCCAGGACCACAGCAACAGGCATTTAAGTATCTGAAAGGTCTGGAGGACTTCATAACCAAGAAGGTGGAGCAGAACCAACGCACGCTAGATCCCAACTCCCCGCGGGACTTCATCGACTCCTTCCTCATCCGCATGCAGGAGGAGCAGAACAACCCCAACACAGAGTTCTACTTGAAGAACCTGGTGCTGACCACACTGAACCTCTTCTTTGCGGGCACTGAGACAGTCAGCACGACCCTGCGGTATGGCTTCCTGCTGCTCATGAAGCACCCAGATGTGGAGGCCAAAATCCACGAGGAGATTGACCAGGTGATTGGCAAGAACCGGCAGCCCAAGTTTGAGGACAGGGCCAAGATGCCCTACACAGAGGCGGTGATCCATGAGATCCAAAGATTTGGAGACATAATCCCCCTGAGCCTGGCCCGCAGAGTCACCAAGGACACCAAGTTTCGGGAGTTCTTCCTCCCCAAGGGCACTGAAGTGTTCCCTATGCTGGGCTCTGTGCTGAGAGACCCCAAGTTCTTTTCCCACCCCCGAGACTTCCACCCCCAGCACTTCCTGGATGAGAAGGGCCAGTTTAAGAAGAGTGATGCTTTTGTGCCCTTCTCCATCGGAAAGCGGTACTGTTTCGGAGAAGGTCTGGCTAGAATGGAGCTCTTTCTCTTCCTCACCACCATCTTGCAGAACTTCTGCCTCAAGTCCCCTCAGCTGCCCCAAGATATTGACGTGTCCCCCAAACTTGTGGGCTTAGCCACTATCCCACGAAATTACACCATGAGCTTCCAGCCCCGCTGA------------>Homo sapiens CYP1A1 NM 000499------------------------ATGCTTGCCACGGAGTTTCTTCTGGCCTCTGTCATCTTCTGTCTGGTATTCTGGGTAATCAGGGCCTCAAGACCTCAGGTCCCCAAAGGCCTGAAGAATCCACCAGGGCCATGGGGCTGGCCTCTGATTGGGCACATGCTGACCCTGGGA---AAGAACCCGCACCTGGCACTGTCAAGGATGAGCCAGCAGTATGGGGACGTGCTGCAGATCCGAATTGGCTCCACACCCGTGGTGGTGCTGAGCGGCCTGGACACCATCCGGCAGGCCCTGGTGCGGCAGGGCGATGATTTCAAGGGCCGGCCCGACCTCTACACCTTCACCCTCATCAGTAATGGTCAGAGCATGTCCTTCAGCTCTGGACCAGTGTGGGCTGCCCGCCGGCGCCTGGCCCAGAATGGCCTGAAAAGTTTCTCCATTGCCTCTGACTACCTGGAAGAGCATGTGAGCAAGGAGGCTGAGGTCCTGATAAGCACGTTGCAGGAGCTGATGGCA------GGGCACTTTAACCCCTACAGGTATGTGGTGGTATCAGTGACCAATGTCATCTGTGCCATTTGCTTTGGCCGGCGCTATGACCACAACCACCAAGAACTGCTTAGCCTAGTCAACCTGAATAATAATTTCGGGGAG------GTGGTTGGCTCTGGAAACCCAGCTGAC---TTCATCCCTATTCTTCGCTACCTACCCAACCCTTCCCTGAATGCCTTCAAGGACCTGAATGAGTTCTACAGCTTCATGCAGAAGATGGTCAAGGAGCACTACAAAACCTTTGAGAAGGGCCACATCCGGGACATCACAGACAGCCTGATTGAGCACTGTCAGGAGAAGCAGCTGGATGAGAACGCCAATGTCCAGCTGTCAGATATCATTAACATCGTCTTGGACCTCTTTGGAGCTGGGTTTGACACAGTCACAACTGCTATCTCCTGGAGCCTCATGTATTTGGTGATGAACCCCAGGGTACAGAGAAAGATCCAAGAGGAGCTAGACACAGTGATTGGCAGGTCACGGCGGCCCCGGCTCTCTGACAGATCCCATCTGCCCTATATGGAGGCCTTCATCCTGGAGACCTTCCGACACTCTTCCTTCGTCCCCTTCACCATCCCCCACAGCACAACAAGAGACACAAGTTTGAAAGGCTTTTACATCCCCAAGGGGCGTTGTGTCTTTGTAAACCAGTGGCAGATCAACCATGACCAGAAGCTATGGGTCAACCCATCTGAGTTCCTACCTGAACGGTTTCTCACCCCTGATGGTGCTATCGACAAGAGTGAGAAGGTGATTATCTTTGGCATGGGCAAGCGGAAGTGTATCGGTGAGACCATTGCCCGCTGGGAGGTCTTTCTCTTCCTGGCTATCCTGCTGCAACGGGTGGAATTCAGCGTGCCACTGGGCGTGAAG---GTGGACATGACCCCCATCTAT---GGGCTAACCATGAAGCATGCCTGCTTCCAAATGCAGCTGCGCTCTTAG--------------->Homo sapiens CYP1A2 NM_000761ATGGCATTGTCCCAGTCTGTTCCCTTCTCGGCCACAGAGCTTCTCCTGGCCTCTGCCATCTTCTGCCTGGTATTCTGGGTGCTCAAGGGTTTGAGGCCTCGGGTCCCCAAAGGCCTGAAAAGTCCACCAGAGCCATGGGGCTGGCCCTTGCTCGGGCATGTGCTGACCCTGGGG---AAGAACCCGCACCTGGCACTGTCAAGGATGAGCCAGCGCTACGGGGACGTCCTGCAGATCCGCATTGGCTCCACGCCCGTGCTGGTGCTGAGCCGCCTGGACACCATCCGGCAGGCCCTGGTGCGGCAGGGCGACGATTTCAAGGGCCGGCCTGACCTCTACACCTCCACCCTCATCACTGATGGCCAGAGCTTGACCTTCAGCTCTGGACCGGTGTGGGCTGCCCGCCGGCGCCTGGCCCAGAATGCCCTCAACACCTTCTCCATCGCCTCTGACCCACTGGAGGAGCATGTGAGCAAGGAGGCTAAGGCCCTGATCAGCAGGTTGCAGGAGCTGATGGCAGGGCCTGGGCACTTCGACCCTTACAATCAGGTGGTGGTGTCAGTGGCCAACGTCATTGGTGCCATGTGCTTCGGACAGCACTTCCCTGAGAGTAGCGATGAGATGCTCAGCCTCGTGAAGAACACTCATGAGTTCGTGGAG------ACTGCCTCCTCCGGGAACCCCCTGGAC---TTCTTCCCCATCCTTCGCTACCTGCCTAACCCTGCCCTGCAGAGGTTCAAGGCCTTCCAGAGGTTCCTGTGGTTCCTGCAGAAAACAGTCCAGGAGCACTATCAGGACTTTGACAAGAACAGTGTCCGGGACATCACGGGTGCCCTGTTCAAGCACAGCAAGAAGGGGCCTAGAGCCAGCGGCAACCTCATCCCACAGGAGAAGATTGTCAACCTTGTCAATGACATCTTTGGAGCAGGATTTGACACAGTCACCACAGCCATCTCCTGGAGCCTCATGTACCTTGTGACCAAGCCTGAGATACAGAGGAAGATCCAGAAGGAGCTGGACACTGTGATTGGCAGGGAGCGGCGGCCCCGGCTCTCTGACAGACCCCAGCTGCCCTACTTGGAGGCCTTCATCCTGGAGACCTTCCGACACTCCTCCTTCTTGCCCTTCACCATCCCCCACAGCACAACAAGGGACACAACGCTGAATGGCTTCTACATCCCCAAGAAATGCTGTGTCTTCGTAAACCAGTGGCAGGTCAACCATGACCCAGAGCTGTGGGAGGACCCCTCTGAGTTCCGGCCTGAGCGGTTCCTCACCGCCGATGGCACTGCCATTAACAGTGAGAAGATGATGCTGTTTGGCATGGGCAAGCGCCGGTGTATCGGGGAAGTCCTGGCCAAGTGGGAGATCTTCCTCTTCCTGGCCATCCTGCTACAGCAACTGGAGTTCAGCGTGCCGCCGGGCGTGAAA---GTCGACCTGACCCCCATCTACGGGCTGACCATGAAGCACGCCCGCTGTGAACATGTCCAGGCGCGGCTGCGCTTCTCCATCAATTGA>Homo sapiens CYP2A13 NM 000766------------------------ATGCTGGCCTCAGGGCTGCTTCTGGTGACCTTGCTGGCCTGCCTGACTGTGATGGTCTTGATGTCAGTCTGGCGGCAGAGGAAGAGCAGGGGGAAGCTGCCTCCGGGACCCACCCCATTGCCCTTCATTGGAAACTACCTGCAGCTGAACACAGAGCAGATGTACAACTCCCTCATGAAGATCAGTGAGCGCTATGGCCCTGTGTTCACCATTCACTTGGGGCCCCGGCGGGTCGTGGTGCTGTGCGGACATGATGCCGTCAAGGAGGCTCTGGTGGACCAGGCTGAGGAGTTCAGCGGGCGAGGCGAGCAGGCCACCTTCGACTGGCTCTTCAAAGGCTATGGCGTGGCGTTCAGCAACGGGGAGCGCGCCAAGCAGCTCCGGCGCTTCTCCATCGCCACCCTAAGGGGTTTTGGCGTGGGCAAGCGCGGCATCGAGGAACGCATCCAGGAGGAGGCGGGCTTCCTCATCGACGCCCTCCGGGGCACGCACGGC------GCCAATATCGATCCCACCTTCTTCCTGAGCCGCACAGTCTCCAATGTCATCAGCTCCATTGTCTTTGGGGACCGCTTTGACTATGAGGACAAAGAGTTCCTGTCACTGTTGCGCATGATGCTGGGAAGCTTCCAGTTCACGGCAACCTCCACGGGGCAGCTCTATGAGATGTTCTCTTCGGTGATGAAACACCTGCCAGGACCACAGCAACAGGCCTTTAAGGAGCTGCAAGGGCTGGAGGACTTCATCGCCAAGAAGGTGGAGCACAACCAGCGCACGCTGGATCCCAATTCCCCACGGGACTTCATCGACTCCTTTCTCATCCGCATGCAGGAGGAGGAGAAGAACCCCAACACAGAGTTCTACTTGAAGAACCTGGTGATGACCACCCTGAACCTCTTCTTTGCGGGCACTGAGACCGTGAGCACCACCCTGCGCTACGGTTTCCTGCTGCTCATGAAGCACCCAGAGGTGGAGGCCAAGGTCCATGAGGAGATTGACAGAGTGATCGGCAAGAACCGGCAGCCCAAGTTTGAGGACCGGGCCAAGATGCCCTACACAGAGGCAGTGATCCACGAGATCCAAAGATTTGGAGACATGCTCCCCATGGGTTTGGCCCACAGGGTCAACAAGGACACCAAGTTTCGGGATTTCTTCCTCCCTAAGGGCACTGAAGTGTTCCCTATGCTGGGCTCCGTGCTGAGAGACCCCAGGTTCTTCTCCAACCCCCGGGACTTCAATCCCCAGCACTTCCTGGATAAGAAGGGGCAGTTTAAGAAGAGTGATGCTTTTGTGCCCTTTTCCATCGGAAAGCGGTACTGTTTTGGAGAAGGCCTGGCCAGAATGGAGCTCTTTCTCTTCTTCACCACCATCATGCAGAACTTTCGCTTCAAGTCCCCTCAGTCGCCTAAGGATATCGACGTGTCCCCCAAACACGTGGGCTTTGCCACGATCCCACGAAACTACACCATGAGCTTCCTGCCCCGCTGA------------>Homo sapiens CYP2A6 NM 000762------------------------ATGCTGGCCTCAGGGATGCTTCTGGTGGCCTTGCTGGTCTGCCTGACTGTAATGGTCTTGATGTCTGTTTGGCAGCAGAGGAAGAGCAAGGGGAAGCTGCCTCCGGGACCCACCCCATTGCCCTTCATTGGAAACTACCTGCAGCTGAACACAGAGCAGATGTACAACTCCCTCATGAAGATCAGTGAGCGCTATGGCCCCGTGTTCACCATTCACTTGGGGCCCCGGCGGGTCGTGGTGCTGTGTGGACATGATGCCGTCAGGGAGGCTCTGGTGGACCAGGCTGAGGAGTTCAGCGGGCGAGGCGAGCAAGCCACCTTCGACTGGGTCTTCAAAGGCTATGGCGTGGTATTCAGCAACGGGGAGCGCGCCAAGCAGCTCCGGCGCTTCTCCATCGCCACCCTGCGGGACTTCGGGGTGGGCAAGCGAGGCATCGAGGAGCGCATCCAGGAGGAGGCGGGCTTCCTCATCGACGCCCTCCGGGGCACTGGCGGC------GCCAATATCGATCCCACCTTCTTCCTGAGCCGCACAGTCTCCAATGTCATCAGCTCCATTGTCTTTGGGGACCGCTTTGACTATAAGGACAAAGAGTTCCTGTCACTGTTGCGCATGATGCTAGGAATCTTCCAGTTCACGTCAACCTCCACGGGGCAGCTCTATGAGATGTTCTCTTCGGTGATGAAACACCTGCCAGGACCACAGCAACAGGCCTTTCAGTTGCTGCAAGGGCTGGAGGACTTCATAGCCAAGAAGGTGGAGCACAACCAGCGCACGCTGGATCCCAATTCCCCACGGGACTTCATTGACTCCTTTCTCATCCGCATGCAGGAGGAGGAGAAGAACCCCAACACGGAGTTCTACTTGAAAAACCTGGTGATGACCACGTTGAACCTCTTCATTGGGGGCACCGAGACCGTCAGCACCACCCTGCGCTATGGCTTCTTGCTGCTCATGAAGCACCCAGAGGTGGAGGCCAAGGTCCATGAGGAGATTGACAGAGTGATCGGCAAGAACCGGCAGCCCAAGTTTGAGGACCGGGCCAAGATGCCCTACATGGAGGCAGTGATCCACGAGATCCAAAGATTTGGAGACGTGATCCCCATGAGTTTGGCCCGCAGAGTCAAAAAGGACACCAAGTTTCGGGATTTCTTCCTCCCTAAGGGCACCGAAGTGTACCCTATGCTGGGCTCTGTGCTGAGAGACCCCAGTTTCTTCTCCAACCCCCAGGACTTCAATCCCCAGCACTTCCTGAATGAGAAGGGGCAGTTTAAGAAGAGTGATGCTTTTGTGCCCTTTTCCATCGGAAAGCGGAACTGTTTCGGAGAAGGCCTGGCCAGAATGGAGCTCTTTCTCTTCTTCACCACCGTCATGCAGAACTTCCGCCTCAAGTCCTCCCAGTCACCTAAGGACATTGACGTGTCCCCCAAACACGTGGGCTTTGCCACGATCCCACGAAACTACACCATGAGCTTCCTGCCCCGCTGA------------>Homo sapiens CYP2A7 NM 000764------------------------ATGCTGGCCTCAGGGCTGCTTCTGGTGGCCTTGCTGGCCTGCCTGACTGTGATGGTCTTGATGTCTGTCTGGCAGCAGAGGAAGAGCAGGGGGAAGCTGCCTCCGGGACCCACCCCACTGCCCTTCATTGGAAACTACCTCCAGCTGAACACAGAGCACATATGTGACTCCATCATGAAGTTCAGTGAGTGCTATGGCCCCGTGTTCACCATTCACTTGGGGCCCCGGCGGGTCGTGGTGCTGTGTGGACATGATGCCGTCAGGGAGGCTCTGGTGGACCAGGCTGAGGAGTTCAGCGGGCGAGGCGAGCAAGCCACCTTCGACTGGGTCTTCAAAGGCTATGGCGTGGCGTTCAGCAACGGGGAGCGCGCCAAGCAGCTCCTGCGCTTTGCCATCGCCACCCTGAGGGACTTCGGGGTGGGCAAGCGAGGCATCGAGGAGCGCATCCAGGAGGAGTCGGGCTTCCTCATCGAGGCCATCCGGAGCACGCACGGC------GCCAATATCGATCCCACCTTCTTCCTGAGCCGCACAGTCTCCAATGTCATCAGCTCCATTGTCTTTGGGGACCGCTTTGACTATGAGGACAAAGAGTTCCTGTCACTGCTGAGCATGATGCTAGGAATCTTCCAGTTCACGTCAACCTCCACGGGGCAGCTCTATGAGATGTTCTCTTCGGTGATGAAACACCTGCCAGGACCACAGCAACAGGCCTTTAAGTTGCTGCAAGGGCTGGAGGACTTCATAGCCAAGAAGGTGGAGCACAACCAGCGCACGCTGGATCCCAATTCCCCACAGGACTTCATCGACTCCTTTCTCATCCACATGCAGGAGGAGGAGAAGAACCCCAACACGGAGTTCTACTTGAAGAACCTGATGATGAGCACGTTGAACCTCTTCATTGCAGGCACCGAGACGGTCAGCACCACCCTGCGCTATGGCTTCTTGCTGCTCATGAAGCACCCAGAGGTGGAGGCCAAGGTCCATGAGGAGATTGACAGAGTGATCGGCAAGAACCGGCAGCCCAAGTTTGAGGACCGGACCAAGATGCCCTACATGGAGGCAGTGATCCACGAGATCCAAAGATTTGGAGACGTGATCCCCATGAGTTTGGCCCGCAGGGTTAAAAAGGACACCAAGTTTCGGGATTTTTTCCTCCCTAAGGGCACCGAAGTGTTCCCTATGCTGGGCTCCGTGCTGAGAGACCCCAGCTTCTTCTCCAACCCTCAGGACTTCAATCCCCAGCATTTCCTGGATGACAAGGGGCAGTTTAAGAAGAGTGATGCTTTTGTGCCCTTTTCCATCGGAAAGCGGAACTGTTTCGGAGAAGGCCTGGCCAGAATGGAGCTCTTTCTCTTCTTCACCACCGTCATGCAGAACTTCCGCCTCAAGTCCTCCCAGTCACCTAAGGACATTGACGTGTCCCCCAAACACGTGGTCTTTGCCACGATCCCACGAAACTACACCATGAGCTTCCTGCCCCGCTGA------------>Loxodonta africana CYP2A13-like XM 023539929------------------------ATGGAGCTAGGAGGGGCTTTCACTATCTTTCTAGCACTCTGCTTTTCTTGTCTGATTATCCTCATTGCCTGGAAAAGAATCAGCAAGGGAGGGAAGCTGCCCCCAGGCCCCATGCCACTACCTTTCCTGGGAAACATCCTGCAAGTCCGCACTGACGCCACCTTTCAGTCTTTCATGAAGCTCAAGGAGAAATATGGCCCTGTGTTCACTGTGTACATGGGACCCAGGCCAGTAGTTGTTTTATGTGGACATGAAGCAGTGAAGGAGGCCCTGGTAGACCAAGCAGATGATTTCAGTGGCCGTGGAGAATTGGCTTCCGTAGAGCGAAACTTCCTAGGTCATGGTGTAGCCCTGGCTAATGGTGAACGATGGAGGATTCTCCGCCGCTTCTCCCTGACCATTCTCCGGGACTTTGGGATGGGAAAGCGAAGCATTGAGGAGCGGATCCAGGAGGAGGCTGGCTACCTACTGGAGGCATTACGGAAGACCGACGGT------GCCTTCACTGATTCCACCTACTTCCTGAGCCGCACTATCTCCAATGTCATCAGCTCCATCGTCTTCGGAGACCGGCTGGACTAGGAAGATAAAGAGTTCCTGGCACTGCTGCGTATGATGCTGGGAAGCTTCCTTCCTTTCTCCTCTCCCCATCTCCAGCTCTATGACATGTTCTACTCGGTAATGAAATACCTACCAGGGCCACAGCAACAGGCCTTTAAGGAGCTGCAGGGGCTGGAGGACTTCATAACCAAGAAGGTGGAGCAGAACCAGCGCACACTGGACCCCAACTCCCCATGGGACTTCATCGACTCCTTCCTCATCCGCATGCGGGAGGAGAAGAAGAACCCCAACACAGAGTTCTACATGAAGAACCTGGTGCTGACCACACTAAACCTCTTCTTTGCAGGCACAGAGACCATCAGCACCACCCTGCGCTACGGCTTCCTGCTGCTCATGAAGCACCCAGATGTGCAGGCCAAGTTGCACGAGGAGATTGACCAGGTGATTGGCAAGAACCGTCAGCCCATGTTTGAGGACCGGGCCAAGATGCCCTACACCGAGGCTGTGATCCACGAGATCCAGAGATTTGCAGACATGATCCCCATGGGGGTGGCCCACAGGGTCACCAAGGACACCAAGTTTCGGGACTTCTTCATCCCCAAGGGCACAGAACTGTTCCCTATGCTGGGCTCCGTGCTGAGAGACACCAAGCTCTTCTCCAACCCCCAAGATTTCAACCCCCGGCACTTCCTGGATGAGAAAGGGCAATTTAAGAAGAACAACATCTTTGTGCCCTTCTCCATTGGAAAGCGGTACTGTTTCGGGGAAGGTCTGGCCAGAATGGAGCTCTTTGTCTTCCTTACCACCATCTTGCAGAACTTCTGCTTCAAGTCCCCGCAGTCGCCCAAGGACATCGATGTGTCTCCCAAACACGTGGGCTTTGCCACTATCCCACGAACCTACACCATGAGCTTCTTGCCCCGCTGA------------>Loxodonta africana CYP2A13 XM 003420788------------------------ATGCTGGCCTCAGGGATGCTTCTGGTGGCTTTGCTGGCCTGCCTGTCTGTAATGGTCTTGATGTCTGTCTGGCGACAAAGGAAGCTCTGGGGGAAGCTTCCCCCTGGGCCCACTCCCTTGCCTTTCATCGGGAACTACCTGCAGCTGAACACACAGCAGATGTACAACTCCCTCATGAAGCTCAGTGAGCGCTATGGCTTGGTGTTCACGGTCCACTTGGGGTCCCGGCGGGTTGTGGTACTGTGCGGATACGACGCTGTGAAGGAGGCTCTGGTGGACCAGGCTGAGGAATTCAGTGGGCGAGGCGAGCAGGCCACCTTCGACTGGCTCTTCAAAGGCTACGGAGTGGCGTTCAGCAACGGGGAGCGAGCCAAGCAGCTCCGGCGCTTCTCCATCACCACGCTGCGGGACTTCGGCGTGGGAAAGCGTGGCATTGAGGAGCGTATCCAGGAGGAGGCCGGCTTCCTCATCGAGACCTTCCGGGGCACGCGCGGC------ACCTTCATTGATCCCACCTACTTCCTGAGCCGAACAGTCTCCAATGTCATCAGCTCCATTGTCTTCGGAGACCGGTTTGACTATGAAGATAAAGAGTTCCTTTCACTGCTGCGTATGATGCTGGGAAGCTTCCAGTTCACAGCTACCGCTACCGGACAGCTCTATGACATGTTCTACTCGGTAATGAAATACCTACCAGGGCCACAGCAACAGGCCTTTAAGGAGCTGCAGGGGCTGGAGGACTTCATAACCAAGAAGGTGGAGCAGAACCAGCGCACACTGGACCCCAACTCCCCACGGGACTTCATTGACTCCTTCCTCATCCGCATGCGGGAGGAGAAGAAGAACCCCAACACAGAGTTCTACATGAAGAACCTGGTGCTGACCACGCTAAACCTCTTCTTTGCGGGCACAGAGACCGTCAGCACAACCCTGCGTTACGGCTTCCTGCTGCTCATGAAGCACCCAGATGTGCAGGCCAAGTTGCACGAGGAGATTGACCAGGTGATTGGCAAGAACCGTCAGCCCAAGTTTGAGGACCGGGCCAAGATGCCCTACACCGAGGCTGTGATCCATGAGATCCAGAGATTTGCAGACATGATCCCCATGGGGGTGGCCCGCAGGGTCACCAAGGACACCAAGTTTCGGAACTTCTTCATCCCCAAGGGCACAGAAGTGTTCCCTATGCTGGGCTCTGTGCTGAGAGACACCAAGTTCTTCTCCAACCCCCAAGATTTCAACCCCCGGCACTTCCTGGATGAGAAAGGGCAATTTAAGAAGAACGACGCCTTTGTGCCCTTCTCCATTGGAAAGCGGTACTGTTTCGGGGAAGGTCTGGCCAGAATGGAGCTCTTTGTCTTCCTTACCACCATCTTGCAGAACTTCTGCTTCAAGTCCCCGCAGTCGCCCAAGGACATCGATGTGTCTCCCAAACACGTGGGCTTTGCCACTATCCCACGAACCTACACCATGAGCTTCTTGCCCCGCTGA------------>O. afer afer CYP2A13 LOC103198761 XM 007942968------------------------ATGCTGGCCTCGGGGCTGCTTCTGGTGGCTCTGCTGGCCTGCTTGTCTGTCATGATCTTGACGTCTGTCTGGCGACAAAGGAAGCTCTGGGGGAAGCTGCCCCCTGGGCCGACTCCATTGCCCTTTATTGGGAACTACCTGCACCTGAATACAGAGCAGATGTACAATTCTCTCATGAAGCTCAGCAAGCGCTACGGTTCCGTGTTCACGGTCCACCTGGGGCCCCGGCGGATCGTGGTACTGTGTGGATACGACGCAGTGAAGGAAGCACTGGTGGACCAAGCTGAGGAATTCAGTGGACGAGGCGAGCAGGCCACCTTCGACTGGCTCTTCAAAGGCTACGGTGTGGCGTTCAGCAACGGTGAACGTGCCAAGCAGCTACGGCGCTTCTCCATCACCACGCTGCGGGACTTCGGCGTGGGCAAACGCGGCATCGAGGAGCGCATCCAGGAGGAGGCGGGCTTCCTCATCGAGACCTTCCGGGGCACGCGCGGC------GCCTTCATCGATCCTACCTACTTCCTGAGCCGCACCGTCTCCAACGTCATCAGCTCCATCGTCTTCGGGGACCGCTTTGACTATGAAGACAAAGAGTTCCTGTCACTGTTGCGTATGATGCTGGGAAGTTTCCAGTTCACAGCTACTTCCACGGGACAGCTCTATGAGATGTTCTCCTCAGTAATGAAACACCTCCCAGGACCACAGCAGCAAGCCTTTAAGGAGCTGCAGGGGCTAGAGGATTTCATAGCCAAGAAGGTGGAGCAGAACCAGCACACGCTGGACCCCAACTCCCCACGGGATTTCATCGATTCCTTCCTCATCCGCATGCAGGAGGAGAAGAACAACCCCAACACGGAGTTCTACATGAAGAACCTGGTTCTGACCACACTGAACCTCTTCTTCGCGGGCACTGAGACCGTCAGCACCACCCTGCGTTACGGCTTCCTGCTGCTCATGAAGCACCCAGATGTGGAGGCCAAGCTGCACGAAGAGATTGACCGGGTGATCGGCAAGAACCGTCAGCCCAAGTTTGAGGACCGGGCCAAGATGCCCTACACAGAGGCAGTGATCCATGAGATCCAGAGATTTGGAGACATGATCCCCATGGGTGTGGCCCGCAGGGTTGTCAAGGACACCAAGTTCCGGGACTTCCTCCTCCCTAAGGGCACTGAAGTGTTCCCCATGCTGGGCTCTGTGCTGAGAGACACCAAGTTCTTCTCCAGCCCCCAAGACTTCAACCCTCAGCACTTCCTGGATGAGAAAGGGCAATTTAAGAAGAATGATGCCTTTGTGCCCTTCTCCATTGGAAAGCGGTACTGTTTCGGGGAAGGTCTGGCCAGAATGGAGCTCTTTCTCTTCCTTACCACCATCCTGCAGAACTTCCATTTCAAGTCCCCGCAGTCACTGAAGGACATCGATGTGTCCCCCAAACATGTGGGTTTTGCCACCATCCCACCAAACTACACTATGAGCTTCCAGCCCCGCTGA------------>Rattus norvegicus Cyp2a1 NM 012692------------------------ATGCTGGACACAGGACTGCTTCTGGTGGTCATATTGGCCTCCCTGAGCGTCATGCTCTTGGTGTCCCTCTGGCAGCAG---AAAATCAGGGGGAGATTGCCTCCAGGACCCACTCCTTTGCCTTTCATTGGAAATTATCTGCAGCTGAATACAAAAGACGTATACAGTTCCATCACACAGCTCAGTGAGCGCTATGGTCCTGTGTTCACCATCCACCTTGGGCCTCGCCGGGTTGTGGTGCTTTATGGATACGATGCAGTCAAAGAGGCTTTGGTGGACCAAGCTGAGGAGTTCAGTGGACGAGGCGAACAGGCTACCTACAATACACTCTTCAAAGGCTATGGCGTGGCATTCAGCAGTGGGGAGCGGGCAAAACAACTCAGGCGCCTCTCTATAGCCACATTGAGAGATTTTGGTGTGGGCAAGCGTGGTGTAGAGGAGCGTATCCTGGAGGAGGCAGGCTATTTGATCAAGATGTTGCAGGGCACTTGTGGA------GCCCCCATTGACCCCACCATCTACCTGAGCAAAACAGTCTCCAATGTTATTAGCTCCATTGTCTTCGGGGAACGCTTCGACTATGAGGACACGGAGTTCCTGTCACTGCTGCAGATGATGGGTCAAATGAACAGATTTGCAGCTTCACCCACAGGGCAGCTCTATGACATGTTCCATTCAGTGATGAAGTACCTGCCTGGACCACAGCAACAGATCATCAAGGTTACTCAGAAACTGGAAGACTTCATGATAGAGAAAGTGAGGCAGAACCATAGTACCCTGGACCCCAATTCCCCAAGGAACTTCATTGACTCCTTTCTCATCCGCATGCAAGAGGAGAAA---AATGGCAATTCAGAGTTCCACATGAAGAACCTAGTGATGACAACACTAAGCCTCTTCTTTGCTGGGTCTGAGACAGTCAGCTCCACACTACGCTACGGCTTCCTTCTACTCATGAAGCATCCAGATGTGGAGGCCAAGGTCCATGAGGAAATTGAGCAGGTGATCGGCAGGAACCGACAGCCTCAGTATGAGGACCACATGAAGATGCCCTACACCCAGGCTGTGATCAATGAGATCCAAAGATTTTCTAACTTGGCTCCCTTGGGCATTCCTCGAAGGATTATCAAGAACACAACCTTCCGTGGCTTCTTCCTCCCCAAGGCCACCGATGTGTTCCCTATATTAGGTTCTCTGATGACAGACCCAAAGTTCTTCCCTAGCCCCAAAGACTTCGACCCCCAGAACTTCCTGGATGACAAGGGACAGTTGAAGAAAAATGCTGCTTTCCTCCCTTTCTCCACTGGGAAGCGATTCTGCTTGGGAGATGGCCTGGCTAAGATGGAGCTCTTCCTGCTGCTCACCACTATTTTACAGAACTTCCGTTTCAAGTTCCCAATGAAACTAGAAGACATCAACGAGTCCCCCAAACCCTTGGGGTTTACCAGGATCATACCAAAGTACACCATGAGCTTCATGCCCATCTGA------------>Rattus norvegicus Cyp2a2 NM 012693------------------------ATGCTGGACACAGGACTGCTCCTGGTGGTCATACTGGCCTCCCTAAGTGTCATGTTCTTGGTGTCCCTCTGGCAGCAG---AAAATCAGGGAGAGATTGCCTCCAGGACCCACTCCTTTGCCTTTCATTGGAAATTATCTGCAGCTGAATATGAAAGACGTATACAGTTCCATCACACAGCTCAGTGAGCGCTATGGTCCTGTGTTCACCATTCACCTTGGGCCTCGACGGATTGTTGTGCTTTATGGATACGATGCAGTCAAAGAGGCTTTGGTGGACCAAGCTGAGGAGTTCAGTGGACGTGGCGAACTGCCTACCTTTAATATACTCTTCAAAGGCTATGGTTTTTCATTGAGCAATGTGGAACAGGCCAAGCGTATCAGGCGCTTCACCATAGCCACATTGAGAGATTTTGGTGTGGGCAAGCGTGATGTACAGGAGTGTATCCTGGAGGAGGCAGGCTATTTGATCAAGACGTTGCAGGGCACTTGTGGA------GCCCCCATTGACCCTTCCATCTACCTGAGCAAAACAGTCTCCAATGTCATTAACTCCATTGTCTTCGGGAACCGCTTCGACTATGAGGACAAAGAGTTCTTGTCACTGTTGGAGATGATCGATGAAATGAATATATTTGCAGCCTCAGCCACAGGGCAGCTCTATGACATGTTCCATTCAGTGATGAAGTACCTGCCTGGACCACAGCAACAGATCATCAAGGTTACTCAGAAACTGGAAGACTTCATGATAGAGAAAGTGAGGCAGAACCATAGTACCCTGGACCCCAATTCCCCAAGGAACTTCATTGACTCCTTTCTCATCCGCATGCAAGAGGAGAAA---TATGTTAATTCAGAATTCCACATGAACAACCTAGTGATGTCATCATTAGGCCTCCTCTTTGCTGGGACTGGGTCAGTCAGCTCCACGCTATACCATGGTTTCCTGCTACTCATGAAGCATCCAGATGTGGAAGCCAAGGTCCATGAGGAAATTGAGCGAGTGATCGGCAGGAACCGACAGCCTCAGTATGAGGACCACATGAAGATGCCCTACACCCAGGCTGTGATCAATGAGATCCAAAGATTTTCTAACTTGGCTCCCTTGGGCATTCCTCGAAGGATTATCAAGAACACAACCTTCCGTGGCTTCTTCCTCCCCAAGGGCACCGATGTATTCCCTATAATAGGTTCTCTGATGACAGAACCAAAGTTCTTCCCTAACCACAAAGACTTCAACCCCCAGCACTTCCTGGATGACAAGGGACAGTTGAAGAAGAATGCTGCATTTCTCCCTTTTTCCATTGGAAAGCGATTCTGCTTGGGAGATAGCCTGGCTAAAATGGAGCTCTTCCTGCTGCTCACCACCATCTTGCAGAACTTCCGTTTTAAGTTCCCAATGAATCTAGAAGACATCAACGAGTACCCCAGTCCCATAGGGTTTACCAGGATCATACCAAATTACACCATGAGCTTCATGCCCATCTGA------------>Rattus norvegicus Cyp2a3 NM 012542------------------------ATGCTGGCCTCAGGACTCCTTCTGGTGGCCTCAGTGGCCTTCCTCAGTGTCCTGGTCTTGATGTCTGTCTGGAAGCAGAGGAAGCTCTCAGGGAAGCTGCCTCCTGGACCCACCCCATTGCCCTTCATCGGGAACTACCTCCAGCTGAACACAGAGAAAATGTACAGCTCTCTCATGAAGATCAGCCAACGTTACGGTCCTGTATTCACCATCCACCTGGGACCTCGCCGAGTTGTGGTGCTGTGCGGACAGGAGGCAGTCAAGGAGGCTCTGGTGGACCAAGCTGAGGAATTCAGTGGTCGGGGAGAGCAGGCCACCTTCGACTGGCTTTTCAAAGGCTATGGGGTAGCCTTCAGCAGCGGGGAGCGAGCCAAACAGCTAAGGCGCTTCTCCATCGCCACGCTGCGGGACTTCGGCGTGGGCAAGCGTGGCATCGAGGAGCGTATCCAAGAGGAGGCGGGCTTTCTCATCGAGTCATTTCGAAAGACGAACGGT------GCCCTCATTGACCCCACCTTCTATCTGAGCCGGACAGTCTCCAATGTCATTAGCTCAATAGTCTTCGGGGACCGCTTCGACTATGAGGACAAAGAGTTCCTGTCACTGCTTCGAATGATGCTGGGAAGCTTCCAGTTCACAGCTACCTCCACGGGGCAGCTCTATGAGATGTTCTCTTCTGTGATGAAACACCTGCCAGGCCCCCAGCAACAGGCCTTTAAGGAGCTGCAGGGGCTGGAGGACTTCATAACCAAGAAGGTGGAACAGAATCAGCGCACGCTGGATCCCAATTCCCCAAGGGACTTCATCGACTCTTTCCTCATCCGAATGCTGGAGGAAAAGAAGAACCCCAATACTGAGTTCTACATGAAGAACTTGGTGCTGACTACCCTAAATCTCTTCTTTGCCGGCACAGAGACCGTCAGCACCACCCTGCGTTACGGCTTTCTGTTGCTCATGAAGCACCCGGATATTGAGGCTAAGGTCCACGAGGAGATTGACCGGGTGATTGGCAGGAACCGGCAGGCCAAGTATGAGGACCGAATGAAGATGCCCTACACGGAGGCTGTGATCCACGAGATCCAGAGATTTGCAGACATGATCCCCATGGGCCTGGCTCGCAGGGTCACCAAGGACACCAAGTTTCGAGAGTTCCTCCTCCCCAAGGGTACTGAAGTATTTCCTATGCTGGGCTCTGTACTGAAAGACCCTAAGTTCTTCTCCAACCCCAACGACTTCAACCCAAAGCACTTCCTAGATGACAAGGGACAGTTTAAGAAGAGTGATGCCTTTGTGCCCTTTTCCATTGGAAAACGGTATTGTTTCGGGGAAGGACTGGCAAGGATGGAACTCTTTCTCTTCCTCACAAACATCATGCAGAACTTCTGCTTCAAATCCCCACAGGCACCCCAGGACATCGATGTGTCTCCTAGACTTGTGGGCTTTGCCACAATCCCACCAAACTACACTATGAGTTTCTTGTCCCGTTGA------------>Sus scrofa CYP2A19 NM 214417------------------------ATGCTGGCCTCAGGCTTGCTTCTCGTGGCTCTGCTGACCTGCCTGACCATAATGGTCTTGATGTCCGTCTGGCGCCAGAGGAAGCTCCAGGGGAAACTGCCCCCCGGACCCACCCCGCTGCCCTTCATCGGGAACTACCTGCAGCTGAACACGGAGCAGATGTACAACTCCCTCATGAAGATCAGCCAGCGCTATGGCCCTGTGTTCACCGTCCACCTGGGGCCCCGGCGGATAGTGGTGCTGTGTGGATACGACGCGGTGAAGGAGGCCCTGGTGGACCAGGCTGAGGAATTCAGCGGGCGAGGCGAGCAGGCCACTTTCGACTGGCTCTTCAAAGGCTATGGCGTGGCCTTCAGCAACGGCGAGCGTGCCAAGCAGCTCCGGCGCTTCTCCATCACCACGCTGCGGGACTTCGGCGTGGGCAAGCGGGGTATCGAGGAGCGCATCCAGGAGGAGGCGGGCCACCTCATCGAGGCCTTCCGGGGCACGCGCGGC------GCGTTCATCGACCCCACCTACTTCCTCAGCCGAACGGTTTCCAATGTCATCAGCTCCATTGTCTTCGGAGACCGCTTTGACTATGAGGACAAAGAGTTCCTCGCACTGCTGCGGATGATGCTGGGAAGCTTTCAGTTCACAGCTACCTCTACCGGACAGCTCTATGAGATGTTCTACTCGGTGATGAAACACCTGCCAGGGCCGCAGCAACAGGCATTTAAGGACCTGCAGGGGCTGGAGGACTTCATAGCCAGGAAGGTGGAACACAACCAGCGCACGCTGGATCCCAACTCCCCGCGAGACTTCATCGACTCCTTCCTCATCCGCATGCAGGAGGAGAAGAAGAATCCTGACACCGAGTTCTATTGGAAGAACCTGGTTCTGACCACACTGAACCTCTTCTTCGCGGGCACCGAGACGGTCAGCACAACGATGCGCTACGGCTTCCTGCTGCTCATGAAGAAACCGGATGTGGAGGCCAAAGTCCACGAGGAGATTGACCGCGTGATCGGCAGGAACCGCCAGGCCAAGTTCGAGGACCGGGCCAAGATGCCCTACACGGAGGCCGTGATCCACGAGATCCAGAGATTCGGAGACATGATCCCCATGGGCCTGGCCCGAAGAGTCACCAAGGATACCAAGTTTCGGGACTTCCTCCTCCCCAAGGGCACTGAGGTGTTCCCTATGCTGGGCTCTGTGCTGAGAGACCCCAAGTTCTTCTCCAACCCCCGAGGCTTCAACCCCCAGCACTTCCTGGATGAGAACGGGCAGTTTAAGAAGAATGATGCTTTTGTGCCCTTCTCCATCGGAAAGCGGTACTGTTTCGGAGAAGGTCTGGCTAGAATGGAGCTCTTCCTCTTCCTCACCAACATCCTGCAGAACTTCCACCTCAAGTCTCCGCAGCTGCCCCAGGACATCGACGTGTCCCCCAAACACGTGGGCTTCGCCACCATCCCCCCGACCTACACCATGAGCTTCCAGCCCCGCTGA------------
