# Supplementary material for: A Comparative Genomic and Phylogenetic Investigation of the Xenobiotic Metabolism Enzymes of Cytochrome P450 in Elephants Shows Loss in CYP2E and CYP4A
Source: Animals (Basel). 2023 Jun 9;13(12):1939. doi: 10.3390/ani13121939 (PMC10294912; doi:10.3390/ani13121939)
Supplement: Supplementary file 1 [file animals-13-01939-s001.zip › CYP seq file/CYP3A.txt]

>Bos taurus CYP3A24 XM 024985097---ATGGAGCTAATCCCAAGCTTTTCCATGGAAACCTGGGTTCTCCTGTCTATCAGCCTGGTGCTCCTTTATCTATATGGAACTTATTCACACGGACTATTTAAGAAGCTGGGAGTTCCTGGGCCAAGACCTCTGCCTTATTTTGGAAATGTTCTGTCCTACCGAAAGGGTGTTTGTGAGTTTGATGAAGAATGTTTTAAAAAGTATGGGAAAATGTGGGGGATTTTTGAAGGTAAACATCCTCTGTTGGTTATCACAGATCCAGATGTGATCAAAACAGTACTAGTGAAAGAATGTTATTCTGTCTTCACAAACCGGAAGGTTTTTGGTCCAAGGGGAATTATGAAAAATGCTGTTTCTGTGGCTGAGGATGAACAATGGAAGAGAATACGGACATTGCTCTCTCCAGCCTTCACCAGTGGGAAGCTCAAGGAGATGTTCCCTATCATTGGGAAGTATGGAGATGTGTTGGTGAGGAACCTGAGGAAGGAAGCAGAAAAAGGCAAGTCCGTCAACATGAAAGAGGTCTTTGGGGCCTACAGCATGGATGTGATTACTAGCACATCATTTGGAGTGAATATTGATTCCCTTGGCAACCCGCAAGATCCCTTTGTGGAGAATGCCAAGAAGCTCTTAAGATTTGATATCCTCAATCCATTTCTTCTCTCAGTAGTACTCTTTCCATTCCTTGTCCCAATCTTTGAAGTATTAAATATCACCATGTTTCCAAAAAGTGCTGTGAATTTTTTGGCAAAATCTGTAAAAAGAATAAAAGAAAGTCGCCTCAAAGATAATCAAAAGCCACGTGTGGATTTTCTTCAGCTGATGATTAACTCCCAGAATTCCAAAGAAACAGACAATCATAAAGCTCTCTCTGACCAAGAACTCATGGCCCAAAGTGTTATCTTTATTTTTGCTGGCTATGAGACCACTAGCAATACTCTTTCCTTCCTTTTGTATATTTTGGCCACTCACCCTGATGTCCAGCAGAAGCTGCAGGAGGAAATTGATGTGACTTTCCCCAATAAGGCCCCTCCGACCTATGATGTCCTGGCACAGATGGAGTATCTTGACATGGTGGTGAATGAGACTCTCAGAATGTTCCCAATTACTGTTAGACTTGATAGGCTCTGTAAGAAGGATGTGAAAATCCATGGAGTGTCCATTCCCAAAGGGACAACAGTGACGGTGCCAATCTCTGTGCTTCACAGAGACCCACAGCTCTGGCCAGAGCCTGAGGAGTTCCGTCCTGAAAGGTTCAGTAAGAAGAACAAGGACACCATAAGTCCTTATGTATACCTGCCTTTTGGAACTGGACCCCGAAATTGCATTGGCATGAGGTTTGCCATCATGAATATGAAACTTGCTGTTGTCAGAGTCCTGCAGAACTTCTCCTTCAAATCTTGTAAAGAAACACAGATCCCCTTGAAGATAAACAGTCAAGGACTTATAAGACCAGAAAAACCCATTTTTCTGAAGGTTGTGCTCAGAGATGAGACCATAAGTGGAGCTTGA--------------------->Bos taurus CYP3A4 NM 001099367 XM 010825838 XM 584699---ATGGAGCTAATCCTGAGCTTTTCCACGGAAACCTGGGTTCTCCTGGCTACCGGTCTGGTGCTCCTCTATCTATATGGAACATATTCCTATGGGCTTTTTAAGAAGTTGGGAGTTCCTGGACCAAGACCTCTACCTTATTTTGGAAACATTCTGTCCTACCGAAAGGGTGTATGTGAGTTTAATGAAGAATGTTTTAAAAAGTATGGGAAAATTTGGGGGATTTTTGAAGGTAAACAACCTCTGTTGGTTATCACAGATCCAGACATGATCAAAACAGTACTAGTGAAAGAATGTTATTCTGTCTTCACAAACCGGAGGGTTTTTGGTCCATCGGGAGTTATGAAAAATGCTATTTCTGTGGCTGAGGATGAACAATGGAAGAGAATACGGACATTGCTGTCTCCAACCTTCACCAGTGGGAAGCTCAAGGAGATGTTCCCTATCATTGGGAAGTATGGAGATGTGTTGGTGAGGAACCTGAGGAAGGAAGCAGAGAAAGGCACGTCTGTCGACATTAAAGACATCTTTGGAGCCTACAGCATGGATGTGATTACTAGCACATCATTTGGAGTGAATATTGATTCGCTCAGCAACCCACAAGATCCCTTTGTGGAGAATGTCAAGAAGCTCTTAAGATTTAGTATCCTCGATCCCTTTCTTCTCGCAGTAGTACTCTTTCCATTCCTTGTCCCAATCCTTGATGTATTAAATATCACCATATTTCCAAAAAGTGTTGTGAATTTTTTTACAAAATCTGTAAAAAGGATAAAAGAAAGTCGCCTCAAAGATAATCAAAAGCCACGTGTGGACTTTCTTCAGCTGATGATTAACTCCCAGAATTCCAAAGAAACAGACAATCATAAAGCTCTCTCTGACCAAGAACTCATAGCCCAGAGTATTATCTTTATTTTTGCTGGCTATGAGACCACTAGCAGTACTCTTTCCTTCCTTCTGTATATTTTGGCCACTCACCCTGATGTCCAGCAGAAGCTGCAGGAGGAGATTGATGCAACTTTCCCCAATAAGGCCCCTCCGACCTATGATGTCCTGGCACAGATGGAGTACCTTGACATGGTGGTGAATGAGACTCTCAGAATGTTTCCTATTGCTATTAGACTTGAGAGGCTCTGTAAGAAGGATGTGGAAATCCATGGGGTGTCCATTCCCAAAGGGACAACAGTGATGGTGCCAATCTCCGTGCTGCACAAAGACCCACAGCTCTGGCCAGAGCCTGAGGAGTTCCGTCCTGAAAGGTTCAGTAAGAAGAACAAGGACAGCATAAATCCTTACGTCTACCTGCCTTTTGGAACTGGACCCCGAAATTGCATTGGCATGAGGTTTGCTATCATGAACATGAAACTTGCTATCGTCAGAGTCCTGCAGAACTTCTCCTTCAAACCTTGTAAAGAAACACAGATCCCCTTGAAAATAAGCAGTCAAGGAGTTTTAAGACCGGAAAAACCTGTTGTCCTAAAGGTTGTGCTCAGAGATGGAACCATAAGTGGAGCCTGA--------------------->Bos taurus CYP3A5 NM 001075888 XM 605055---ATGGAGCTAATCCCAAACTTTTCCGTGGAAACCTGGGTTCTCCTGGCTATCAGCCTGGTGCTCCTCTATCTATATGGAACTTATTCACATGGACTGTTTAAGAAGCTGGGGGTTCCTGGCCCAAGACCTCTGCCTCTTTTTGGAAACGTTCTATCCTACCGAAAGGGTGTTTGTGAGTTTGATGAAGAATGTTTTAAAAAGTATGGGAAAATGTGGGGGATTTTTGAAGGTAAACATCCTCTGTTGGTTATCACGGATCCAGACATGATCAAAACAGTACTAGTGAAAGAATGTTATTCTGTCTTCACAAACCGGAGGGTTTTTGGTCCAATGGGAGTTATGAAAAATGCTGTTTCTGTGGCTGAGGATGAACAATGGAAGAGAATACGGACATTGCTGTCTCCAACCTTCACCAGTGGGAAGCTCAAGGAGATGTTCCCTATCATTGGGAAGTATGGAGATGTGTTGGTGAGGAACCTGAGGAAGGAAGCAGAGAAAGGCACGTCCGTCGACATGAAAGAGGTCTTTGGGGCCTACAGCATGGATGTGATTACTAGCACATCATTTGGAGTGAATATTGATTCCCTTGGCAACCCACAAGATCCCTTTGTGGAGAATGCCAAGAAGCTCTTAAGATTTGATATCCTTGATCCATTTCTACTTTCAGTAGTACTCTTTCCATTCCTTATCCCAATCTTTGAAGTATTAAATATCAGCATATTTCCAAAAAGTGCTGTGAATTTTTTGACAACATCCGTAAAAAAGATAAAAGAAAGTCGCCTCAAAGATACTCAAAAGCCGCGTGTGGACTTTCTTCAGCTGATGATTAACTCCCAGAATTCCAAAGAAACAGACAATCATAAAGCTCTCTCTGACCAAGAACTCATGGCCCAGAGTATTATCTTTATTTTTGGTGGCTATGAGACCACTAGCACTTCTCTTTCCTTCATTATATATGAATTGGCCACTCACCCTGATGTCCAGCAGAAGCTGCAGGAGGAGATTGATGCGACTTTCCCCAATAAGGCGCCTCCGACCTATGATGTCCTGGCACAGATGGAGTATCTTGACATGGTGGTGAATGAGACTCTCAGAATGTTTCCTATTGCTGTTAGACTTGAGAGGTTCTGTAAGAAGGATGTGGAAATCCATGGGGTGTCCATTCCCAAAGGGACAACGGTGACGGTGCCAATCTCTGTGCTTCACAGAGACCCACAGCTCTGGCCAGAGCCTGAGGAGTTCCGTCCTGAAAGGTTCAGTAAGAAGAACAAGGACAGCATAAATCCTTACGTCTACCTGCCTTTTGGAACTGGACCCCGAAATTGCATTGGCATGAGGTTTGCTATCATGAACATGAAACTTGCTGTTGTCAGAGTCCTGCAGAACTTCTCCTTCAAACCTTGTAAAGAAACACAGATCCCCTTGAAAATAAAAAGTCAAGGACTTTTAAGACCGGAAAAACCCATTGTTCTGAAGGTTGTGCTCAGAGATGAGACCATAAGTGGAGCTTGA--------------------->Bos taurus CYP3A5 NM 174531 XM 606182 XM 612901---ATGGAACTGATCCCAAGCTTTTCCATGGAAACCTGGGTTCTCCTAGCTACCAGCCTGGTGCTCCTCTATATATATGGGACTTATTCATATGGACTTTTTAAGAAGCTGGGGATTCCTGGGCCAAGACCTGTGCCCTATTTTGGAAGTACTATGGCTTACCACAAGGGTATTCCAGAGTTTGACAATCAGTGTTTTAAAAAGTATGGGAAAATGTGGGGGTTTTATGAAGGTCGACAACCTATGTTGGCTATCACAGATCCTGATATAATCAAAACAGTACTAGTGAAAGAATGTTATTCTGTCTTCACAAACCGGAGGATTTTTGGTCCAATGGGAATTATGAAATATGCCATCTCTCTGGCTTGGGATGAACAATGGAAGAGAATACGGACATTGCTGTCTCCAGCCTTCACCAGCGGAAAGCTCAAGGAGATGTTCCCCATCATTGGCCAGTATGGAGATATGTTGGTGAGGAACCTGAGGAAGGAAGCAGAGAAAGGCAATCCCGTCAACATGAAAGACATGTTTGGAGCCTACAGCATGGATGTGATTACTGGCACAGCATTTGGAGTGAATATTGATTCCCTCAACAACCCACACGATCCCTTTGTGGAACATAGCAAGAACCTCTTAAGATTTAGACCCTTCGATCCATTCATTCTTTCAATAATATTATTTCCATTTCTCAACCCAGTTTTTGAAATATTAAACATTACTCTGTTTCCAAAAAGTACTGTGGACTTTTTCACAAAATCTGTAAAGAAGATTAAAGAAAGTCGCCTCACAGATAAACAAATGAATCGAGTGGATTTACTTCAGCTAATGATTAATTCTCAGAATTCCAAAGAAATTGACAACCATAAAGCTCTGTCTGACATAGAACTCGTGGCCCAAAGTACTATCTTTATTTTTGGTGGCTATGAGACCACTAGCAGTACTCTTTCCTTCATTATATATGAATTGACCACTCACCCTCATGTCCAGCAGAAGGTGCAGGAGGAAATTGATGCAACTTTCCCTAATAAGGCCCCTCCCACCTACGATGCCCTGGTGCAGATGGAGTATCTTGACATGGTGGTGAATGAGACTCTGAGAATGTTTCCAATTGCTGGGAGACTTGAGAGGGTCTGTAAGAAGGATGTGGAAATCCACGGGGTGACCATTCCCAAAGGGACAACCGTGCTGGTGCCACTCTTCGTTCTTCACAACAACCCAGAGCTCTGGCCAGAGCCCGAGGAGTTCCGTCCCGAAAGGTTCAGTAAGAATAACAAGGACAGCATAAATCCTTACGTCTACCTGCCTTTTGGAACTGGACCCCGAAACTGCCTTGGCATGAGGTTTGCCATCATGAACATAAAACTTGCTCTTGTCAGAATCCTGCAGAACTTCTCCTTCAAACCTTGTAAAGAAACACAGATCCCCCTGAAATTATACACTCAAGGACTCACGCAGCCAGAACAACCTGTTATTCTGAAGGTTGTGCCCAGACTGGGACCACAGGTGGAGCCTGACTTTCTCTAA------------>Canis lupus familiaris CYP3A12 XM 038538847 LOC489851---ATGGACCTGATCCCAAGCTTTTCCACAGAAACCTGGCTTCTTCTGGCCACCAGCCTGGTGCTGTTCTACCTCTATGGGACCTACACGCATGGACTTTTTAAGAAGCTGGGAATTCCTGGGCCAACACCTCTGCCTTTTTTGGGAACTGTTCTTGGCTACCGTAATGGTTTTTGTGATTTTGATGAGAAATGTTTTAGAAAGTATGGAAGAATGTGGGGGTTTTATGATGGGCGACAGCCCGTGTTGGCTATCATGGATCCAGACATGATTAAAACAGTGCTAGTGAAAGAATGTTATTCTGTCTTCACAAACCGACGGTCCTTTGGTCCAGTGGGATTTATGAAAAGTGCCATCACTGTGTCTGAGGATGAAGAATGGAAGAGAATACGAACATTGCTGTCTCCAACTTTCACCAGCGGAAAGCTCAAGGAGATGTTTCCCATCATTGGCCAGTATGGAGATATGTTGGTGAGGAACCTGAGGAAGGAGGCAGAGAAAGGCAAAGCCATCAGCTTGAAAGACATCTTTGGAGCCTACAGCATGGATGTGATCACCAGCACATCGTTTGGAGTGAACATTGATTCCCTCAACAACCCACAAGATCCGTTTGTGGAAAATGCCAAGAAGCTCTTAAAATTTGATTTCCCTGATCCATTTTTACTCTCCATAATATTGTTTCCATTCCTTACTCCACTATATGAAATGTTAAATATCTGGCTATTTCCAAAGAAAATTACTGATTTTTTCACAAAGTCTGTAAAAAGGATGAAGGAAAGTCGCCTCAAAGATAAACAAAAGCACCGAGTCGATTTTCTGCAACTGATGATTAACTCCCAGAATTCTAAAGAAATGAATACCCACAAAGCTCTGTCTGATCTGGAGCTTGTTGCTCAATCTATTATCTTTGTTGTTGCTGGCTATGAGACCACTAGTACTTCTCTCTGCCTCCTTATGTATGAATTGGCCACTCACCCTGATGTCCAGCAGAAACTGCAGAAGGAGATTGATGCGACTTTCCCCAATAAGGCAGCACCTACTTATGATACTCTTGTACAGATGGAGTATCTTGACATGGTGTTGAATGAAAGTCTCAGATTATACCCAATCACTGGTAGACTTGTAAGGGTCTGTAAGAAAGATGTGGAAATCAGTGGTGTGTTCATTCCTAAAGGGACAGTGGTGATGGTGCCAACCTTTACTCTTCACCAAGACCCGGATATCTGGCCAGAGCCTGAGAAGTTCCAACCTGAAAGGTTCAGTAAGAAGAACAAGGACAGCATAAATCCTTATACATACCTGCCCTTTGGAACTGGACCCCGAAACTGCCTTGGAATGAGATTCGCAATCATGAACATGAAACTTGCCCTCATCAAAGTCCTGCAGAACTTCTCCTTCAAACCTTGTAAAGAAACACAGATTCCCCTGAAATTAAGTTCTCAAGGGTTAATTCGACCAGAAGAACCCATTATTCTCAACGTTGAGCCAAGAGATGGGAGTGTGCGTGGAGCCTGA--------------------->Canis lupus familiaris CYP3A12 XM 038539351 LOC119876349---ATGGACCTGATCCCAAGCTTTTCCACAGAAACCTGGCTTCTTCTGGCCACCAGCCTGGTGCTGTTCTACCTCTATGGGACCTACACGCATGGACTTTTTAAGAAGCTGGGAATTCCTGGGCCAACACCTCTGCCTTTTTTGGGAACTGTTCTTGGCTACCGTAATGGTTTTTGTGATTTTGATGAGAAATGTTTTAGAAAGTATGGAAGAATGTGGGGGTTTTATGATGGGCGACAGCCCGTGTTGGCTATCATGGATCCAGACATGATTAAAACAGTGCTAGTGAAAGAATGTTATTCTGTCTTCACAAACCGACGGTCCTTTGGTCCAGTGGGATTTATGAAAAGTGCCATCACTGTGTCTGAGGATGAAGAATGGAAGAGAATACGAACATTGCTGTCTCCAACTTTCACCAGCGGAAAGCTCAAGGAGATGTTTCCCATCATTGGCCAGTATGGAGATATGTTGGTGAGGAACCTGAGGAAGGAGGCAGAGAAAGGCAAAGCCATCAGCTTGAAAGACATCTTTGGAGCCTACAGCATGGATGTGATCACCAGCACATCGTTTGGAGTGAACATTGATTCCCTCAACAACCCACAAGATCCGTTTGTGGAAAATGCCAAGAAGCTCTTAAAATTTGATTTCCCTGATCCATTTTTACTCTCCATAATATTGTTTCCATTCCTTACTCCACTATATGAAATGTTAAATATCTGGCTATTTCCAAAGAAAATTACTGATTTTTTCACAAAGTCTGTAAAAAGGATGAAGGAAAGTCGCCTCAAAGATAAACAAAAGCACCGAGTCGATTTTCTGCAACTGATGATTAACTCCCAGAATTCTAAAGAAATGAATACCCACAAAGCTCTGTCTGATCTGGAGCTTGTGGCTCAATCTATTATCTTTATTTTTGGTGGCTATGAGACCACTAGTACTTCTCTCTGCCTCCTTATGTATGAATTGGCCACTCAGCCTGATGTCCAGCAGAAACTGCAAAAGGAGATTGATGCGACTTTCCCCAATAAGGCAGCACCCACTTACGAGGCCCTTGTACAGATGGAGTATCTGGACATGGTGTTGAATGAAAGTCTCAGATTATACCCAATTGCTGGTAGACTAGAGAGGGTCTGTAAGAAAGATGTGGAAATCAGTGGTGTGTTCATTCCCAAAGGGACATTGGTGGTGGTGCCAACATTTACTCTTCACCGAGACTTGGATCTCTGGCCAGAGCCTGAGGAGTTCCAACCTGAAAGGTTCAGTAAGAAGAACAAGGACAGCATAAATCCTTATACATACCTGCCCTTTGGAACTGGACCCCGAAACTGCCTTGGAATGAGATTCGCAATCATGAACATGAAACTTGCCCTCATCAAAGTCCTGCAGAACTTCTCCTTCAAACCTTGTAAAGAAACACAGATTCCCCTGAAATTAAGTTCTCAAGGGTTAATTCGACCAGAAGAACCCATTGTTCTCAATGTTGAGCCAAGAGATGGGAGTGTGCGTGTAGCCTGA--------------------->Canis lupus familiaris CYP3A12 XM 038589240 LOC119868939---ATGGACTTGATCCCAAGCTTTTCCACAGAAACCTGGCTTCTTCTGGCTATCAGCCTGGTGCTCCTCTATCTGTATGGGACCTACACACATGGGATTTTTAGGAAGCTGGGAATTCCTGGGCCAACACCTCTGCCTTTTGTGGGAACTGCTCTGGGCTACCGTAATGGTTTTTATGTTTTTGACATGAAATGTTTTAGTAAGTATGGAAGAATGTGGGGGTTTTATGATGGGCGACAGCCTGTGCTGGCTATCACAGATCCGGACATGATCAAACAGNNTACTAGTGAAGAATGTTATTCTGTCTTCACAAACCGGCGGACTCTTGGTCCAGTGGGATTTATGAAAAGTGCGATCTCTCTGTCTGAGGATGAAGAGTGGAAGAGAATACGAACTTTGCTGTCTCCAACCTTCACCACTGGAAAGCTCAAGGAGATGTTCCCCATCATTGGCCAGTATGGAGATGTGTTGGTGAACAACCTGAGGAAGGAGGCAGAGAAAGGCAAGGCCATCAACTTGAAAGATGTCTTTGGAGCCTACAGCATGGATGTGATTACCAGCATATCGTTTGGAGTGAACATTGATTCCCTCAACCACCCACAAGATCCGTTTGTGGAAAATACCAAGAATCTCTTAAAATTTGATTTCCTTGACCCATTTTTATTCTCCATATTACTGTTTCCATTCCTTACTCCGGTTTTTGAAATATTAAATATCTGGCTATTTCCAAAGAAAGTTACTGATTTTTTCAGAAAATCTGTAGAGAGAATGAAGGAAAGTCGCCTAAAAGATAAACAAAAGCACCGAGTGGACTTTCTTCAGCTGATGATTAACTCCCAGAATTCCAAAGAAATGGACACTCATAAAGCTCTATCTGATTTGGAGCTGGTGGCCCAATCTATTGTCTTTATTTTTGCTGGCTATGAGACCACTAGCAGTTGTCTTTCCTTTCTTATGTATGAATTGGCCACTCACCGTGATGTCCAGCAGAAACTGCAGGAGGAGATTGATGCGACTTTCCCCAATAAGGCAGCACCCACTTATGAGGCCCTTGTACAGATGGAGTATCTGGACATGGTATTGAATGAAACTCTCCGATTATACTCAGTCGCTGGTAGACTTGAGAGGGTCTGTAAGAAAGATGTGGAAATCAGTGGTGTGTTCATTCCCAAAGGGACAGTGGTGATGGTGCCAACCTTTATTCTTCATCGAGACCAGAATCTCTGGCCAGAGCCTGAGGAATTCCGACCTGAAAGGTTCAGTAGGAAGAACAAGGACAGCATAAATCCTTATACATACCTGCCTTTTGGAACTGGACCCCGAAACTGCATTGGAATGAGGTTTGCAATCATGAACATGAAACTTGCCCTTGTCAGGGTCCTGCAGAACTTCTCCTTCAAATCTTGTAAAGAAACACAGATCTCCCTGAGAATAAATACTCGAGGGATTATTCAACCTGAAAAACCCGTTGTTCTCAAGGTTGAGCCAAGAGATGGGAGTGTAAGTGGAGCCTGA--------------------->Canis lupus familiaris CYP3A12 XM 038668128 LOC119875773---ATGGAGGTGGGACCAACTAAGACTACCCTGGCTTCTACAATTATCCAAACACTCAGTCGGGCACAGCTGAGCTATGGGACCTACACGCATGGACTTTTTAAGAAGCTGGGAATTCCTGGGCCAACACCTCTGCCTTTTTTGGGAACTGTTCTTGGCTACCGTAATGGTTTTTGTGATTTTGATGAAAAATGTTTTAGAAAGTATGGAAGAATGTGGGGGTTTTATGATGGGCGACAGCCCGTGTTGGCTATCATGGATCCAGACATGATCAAAACAGTGCTAGTTAAAGAATGTTATTCTGTCTTCACAAACCGACAGTCTTTTGGACCAGTGGGATTTATGAAAAGTGCCATCACTGTGTCTGAGGATGAAGAATGGAAGAGAATACGAACATTGCTGTCTCCAACTTTCACCAGCGGAAAGCTCAAGGAGATGTTTCCCATCATTGGCCAGTATGGAGATGTGTTGGTGAGGAACCTGAGGAAGGAGGCAGAGAAAGATAAAGCCATCAGCTTGAAAGACATCTTTGGAGCCTACAGCATGGATGTGATCACCAGCACATCGTTTGGAGTGAACATTGATTCCCTCAACAACCCACAAGATCCGTTTGTGGAAAATGCCAAGAAGCTCTTAAAATTTGATTTCCCTGATCCATTTTTACTCTCCATAATATTGTTTCCATTTCTTACTCCACTATATGAAATGTTAAATATCTGGCTATTTCCAAAGAAAATTACTGATTTTTTCACAAAGTCTGTAAAAAGGATGAAGGAAAGTCGCCTCAAAGATAAACAAAAGCACCGAGTCGATTTTCTGCAACTGATGATTAACTCCCAGAATTCTAAAGAAATGAATACCCACAAAGCTCTGTCTGATCTGGAGCTTGTTGCTCAATCTATTATCTTTGTTGTTGCTGGCTATGAGACCACTAGTACTTCTCTCTGCCTCCTTATGTATGAATTGGCCACTCACCCTGATGTCCAGCAGAAACTGCAGAAGGAGATTGATGCGACTTTCCCCAATAAGGCAGCACCTACTTATGATACTCTTGTACAGATGGAGTATCTTGACATGGTGTTGAATGAAAGTCTCAGATTATACCCAATCACTGGTAGACTTGTAAGGGTCTGTAAGAAAGATGTGGAAATCAGTGGTGTGTTCATTCCTAAAGGGACAGTGGTGATGGTGCCAACCTTTACTCTTCACCAAGACCCGGATATCTGGCCAGAGCCTGAGAAGTTCCAACCTGAAAGGTTCAGTAAGAAGAACAAGGACAGCATAAATCCTTATACATACCTGCCCTTTGGAACTGGACCCCGAAACTGCCTTGGAATGAGATTCGCAATCATGAACATGAAACTTGCCCTCATCAAAGTCCTGCAGAACTTCTCCTTCAAACCTTGTAAAGAAACACAGATTCCCCTGAAATTAAGTTCTCAAGGGTTAATTCGACCAGAAGAACCCATTATTCTCAACGTTGAGCCAAGAGATGGGAGTGTGCGTGGAGCCTGA--------------------->Canis lupus familiaris CYP3A12 XM 038668133---ATGGACCTGATCCCAAGCTTTTCCACAGAAACCTGGCTTCTTCTGGCCACCAGCCTGGTGCTGTTCTACCTCTATGGGACCTACACGCATGGACTTTTTAAGAAGCTGGGAATTCCTGGGCCAACACCTCTGCCTTTTTTGGGAACTGTTCTTGGCTACCGTAATGGTTTTTGTGATTTTGATGAGAAATGTTTTAGAAAGTATGGAAGAATGTGGGGGTTTTATGATGGGCGACAGCCCGTGTTGGCTATCATGGATCCAGACATGATTAAAACAGTGCTAGTGAAAGAATGTTATTCTGTCTTCACAAACCGACGGTCCTTTGGTCCAGTGGGATTTATGAAAAGTGCCATCACTGTGTCTGAGGATGAAGAATGGAAGAGAATACGAACATTGCTGTCTCCAACTTTCACCAGCGGAAAGCTCAAGGAGATGTTTCCCATCATTGGCCAGTATGGAGATATGTTGGTGAGGAACCTGAGGAAGGAGGCAGAGAAAGGCAAAGCCATCAGCTTGAAAGACATCTTTGGAGCCTACAGCATGGATGTGATCACCAGCACATCGTTTGGAGTGAACATTGATTCCCTCAACAACCCACAAGATCCGTTTGTGGAAAATGCCAAGAAGCTCTTAAAATTTGATTTCCCTGATCCATTTTTACTCTCCATAATATTGTTTCCATTCCTTACTCCACTATATGAAATGTTAAATATCTGGCTATTTCCAAAGAAAATTACTGATTTTTTCACAAAGTCTGTAAAAAGGATGAAGGAAAGTCGCCTCAAAGATAAACAAAAGCACCGAGTCGATTTTCTGCAACTGATGATTAACTCCCAGAATTCTAAAGAAATGAATACCCACAAAGCTCTGTCTGATCTGGAGCTTGTGGCTCAATCTATTATCTTTATTTTTGGTGGCTATGAGACCACTAGTACTTCTCTCTGCCTCCTTATGTATGAATTGGCCACTCAGCCTGATGTCCAGCAGAAACTGCAAAAGGAGATTGATGCGACTTTCCCCAATAAGGCAGCACCCACTTACGAGGCCCTTGTACAGATGGAGTATCTGGACATGGTGTTGAATGAAAGTCTCAGATTATACCCAATTGCTGGTAGACTAGAGAGGGTCTGTAAGAAAGATGTGGAAATCAGTGGTGTGTTCATTCCCAAAGGGACATTGGTGGTGGTGCCAACATTTACTCTTCACCGAGACTTGGATCTCTGGCCAGAGCCTGAGGAGTTCCAACCTGAAAGGTTCAGTAAGAAGAACAAGGACAGCATAAATCCTTATACATACCTGCCCTTTGGAACTGGACCCCGAAACTGCCTTGGAATGAGATTCGCAATCATGAACATGAAACTTGCCCTCATCAAAGTCCTGCAGAACTTCTCCTTCAAACCTTGTAAAGAAACACAGATTCCCCTGAAATTAAGTTCTCAAGGGTTAATTCGACCAGAAGAACCCATTGTTCTCAATGTTGAGCCAAGAGATGGGAGTGTGCGTGTAGCCTGA--------------------->Canis lupus familiaris CYP3A26 NM 001003338---ATGGACTTGATCCCAAGCTTTTCCACAGAAACCTGGCTTCTTCTGGCTATCAGCCTGGTGCTCCTCTATCTGTATGGGACCTACACACATGGGATTTTTAGGAAGCTGGGAATTCCTGGGCCAACACCTCTGCCTTTTGTGGGAACTGCTCTGGGCTACCGTAATGGTTTTTATGTTTTTGACATGAAATGTTTTAGTAAGTATGGAAGAATGTGGGGGTTTTATGATGGGCGACAGCCTGTGCTGGCTATCACAGATCCGGACATGATCAAAACAGTACTAGTGAAAGAATGTTATTCTGTCTTCACAAACCGGCGGACTCTTGGTCCAGTGGGATTTATGAAAAGTGCGATCTCTCTGTCTGAGGATGAAGAGTGGAAGAGAATACGAACTTTGCTGTCTCCAACCTTCACCACTGGAAAGCTCAAGGAGATGTTCCCCATCATTGGCCAGTATGGAGATGTGTTGGTGAACAACCTGAGGAAGGAGGCAGAGAAAGGCAAGGCCATCAACTTGAAAGATGTCTTTGGAGCCTACAGCATGGATGTGATTACCAGCATATCGTTTGGAGTGAACATTGATTCCCTCAACCACCCACAAGATCCGTTTGTGGAAAATACCAAGAATCTCTTAAAATTTGATTTCCTTGACCCATTTTTATTCTCCATATTACTGTTTCCATTCCTTACTCCGGTTTTTGAAATATTAAATATCTGGCTATTTCCAAAGAAAGTTACTGATTTTTTCAGAAAATCTGTAGAGAGAATGAAGGAAAGTCGCCTAAAAGATAAACAAAAGCACCGAGTGGACTTTCTTCAGCTGATGATTAACTCCCAGAATTCCAAAGAAATGGACACTCATAAAGCTCTATCTGATTTGGAGCTGGTGGCCCAATCTATTGTCTTTATTTTTGCTGGCTATGAGACCACTAGCAGTTGTCTTTCCTTTCTTATGTATGAATTGGCCACTCACCGTGATGTCCAGCAGAAACTGCAGGAGGAGATTGATGCGACTTTCCCCAATAAGGCAGCACCCACTTATGAGGCCCTTGTACAGATGGAGTATCTGGACATGGTATTGAATGAAACTCTCCGATTATACTCAGTCGCTGGTAGACTTGAGAGGGTCTGTAAGAAAGATGTGGAAATCAGTGGTGTGTTCATTCCCAAAGGGACAGTGGTGATGGTGCCAACCTTTATTCTTCATCGAGACCAGAATCTCTGGCCAGAGCCTGAGGAATTCCGACCTGAAAGGTTCAGTAGGAAGAACAAGGACAGCATAAATCCTTATACATACCTGCCTTTTGGAACTGGACCCCGAAACTGCATTGGAATGAGGTTTGCAATCATGAACATGAAACTTGCCCTTGTCAGGGTCCTGCAGAACTTCTCCTTCAAATCTTGTAAAGAAACACAGATCTCCCTGAGAATAAATACTCGAGGGATTATTCAACCTGAAAAACCCGTTGTTCTCAAGGTTGAGCCAAGAGATGGGAGTGTAAGTGGAGCCTGA--------------------->Canis lupus familiaris CYP3A4 XM 038668135---ATGGACCTAATCCCAAGCTTTTCCATGGAAACCTGGCTTCTCCTGGCTACCAGCCTGGTGCTCCTCTATCTGTATGGGACCTACACACATGGGGTTTTTAAGAAGCTAGGAATTCCTGGACCAACACCTCTGCCTTTTGTGGGAACTGCTCTGGGCTACCGTAAGGGTTTTTCTGTTTTTGATGAGAACTGTTTTAGAAAGTATGGAAGAATGTGGGGGTTTTATGATGGGCGACGGCCTGTGCTTGCTATTACAGATCCGGACATGATCAAAACAGTGCTAGTGAAAGAATGTTATTCTGTCTTCACAAACCGGCGGTCTTTTGGTCCAGTGGGATTTATGAAAAGTGCCATCTCTCTGTCTGAGGATGAAGAATGGAAGAGAATACGAACATTGCTGTCCCCAACCTTCACCAGTGGAAAGCTCAAGGAGATGTTCCCCATCATTGGCCAGTATGGAGATGTGTTGGTGAGGAACCTGAGGAAGGAGGCAGAGAAAGGCAAATCCATCAACTTGAAAGACATCTTTGGAGCCTACAGCATGGATGTGATTACCAGCACATCATTTGGAGTGAACATTGATTCCCTCAACAACCCACAAGATCCCTTTGTGGAAAATATCAAGAAGCTCTTAAAATTTGATTTCCTTGATCCATTTTTCTTCTCAATATTACTGTTTCCATTCCTTACCCCAGTTTTTGAAGTATTAAATATCTGGCTCTTTCCAAAAAGTGTTACTGATTTTTTCACAAAATCTGTAAAAAGAATGAAGGAAAATCGCCTCAAAGATAAACAAAAGCATCGAGTGGACTTTCTTCAGCTGATGATTAACTCCCAGAATTCTAAAGAAACAGACACTCATAAAGCTCTATCTGATTTGGAGCTGGTGGCCCAATCTATTATCTTTATTTTTGCTGGCTATGAGACCACTAGCACTTCTCTTTCCTTCCTTATGTATGAATTGGCCACTCACCCTGATGTCCAGCAGAAACTGCAGGAGGAGATTGATGCGACTTTCCCCAATAAGGCATTGCCCACTTACGATGCCCTTGTGCAGATGGAATATCTGGACATGGTGTTGAATGAAACTCTCCGATTATACCCAATCGCTGGTAGACTTGAGAGGGTCTGTAAGAAAGATGTGGAAATCAGTGGTGTGTTCATTCCCAAAGGGACAGTGGTGATGGTGCCAACCTTTACTCTTCATCGAGACCAGAGTCTCTGGCCAGAGCCTGAGGAATTCCGACCTGAAAGGTTCAGTAAGGAGAAGAAGGACAGCATAAATCCTTATACATACCTGCCTTTTGGAACTGGACCCCGAAACTGCATTGGAATGAGGTTTGCGATCATGAACACGAAACTTGCCCTTGTCAGGGTCCTGCAGAACTTCTCCTTCAAACCTTGTAAAGAAACACAGATCCCCCTGAAATTAAATGCTCAAGGGATTATTCAACCTGAAAAGCCCATTGTTCTCAAGGTTGAGCCAAGAGATGGGAGTGTAAATGGAGCCTGA--------------------->Elephantulus edwardii CYP3A12 XM 006889615 LOC102869879---ATGGACCTGATCCCAAGCTTTTCCACCGAGACCTGGATTCTCCTGGTCACCAGCCTGCTGCTCGTTTATCTCTATGGAATCCGCAAACACAGTGTCCTGAAGAAGCTTGGGATTCCTGGCCCCAAACCTCTGCCCTTTGTAGGAACCGTCCTGGCCTACCGCAAGAGTATTTGGGAATTTGACATGCAAAATTCCAAAAAATACGGAAAACTGTGGGGGTTTTATGACGGTTCGCAGCCCGTGATCGCCATCACAGACCCAAACACCATCAAGACGATCTGGGTGAAAGAATGCTATTCCATCTTCACGAACCGGAGGGCTTTTGGTCCTGTGGGATTCATGAAATCTGCTGTTTCTGTGGCTGAAGATGAAGAGTGGAAGAGGATGCGAACGTTGCTGTCTCCGACCTTCACCAGTGGAAAACTCAAGGAGATGTTCCCCATCCTCCGCCAGTATGGAGAAGTAATGGTGAAGCATCTGAGGGAAAAAACAGAGAAGGGCCAACCTGTCGAGCTGAAAAGCCTCTTCGGGGCCTACAGCATGGATGTGATCACCAGCACGTCGTTTGGCGTCAACGTCGATTCCCTCAACAACCCAGAAGATCCCTTTGTGCAAAATGTCAAGAAGCTCATAAAATTTGATTTCCTGGACCCATTT---TTCCTTACAGCCGTCTTTCCATTCCTTACTCCTGTTTTCGAAGCATTAAATATTTCTGTGTTTCCGAGAGATGTTACTGATTTTTTCAAAAAGTCTGTAAAAAGAATGAAAGAAGAGCGCCTTAAAGAAAACAAGAAGAACCGGGTGGATTTACTTCAGCTGATGATTGACTCCCAGAAGTCCAAAGAA------ACCCATAAAGCTTTGACAGATCTGGAGCTCGTGGCGCAGTCAATCATCTTCATCTTTGCTGGCTATGAGACCACCAGCACCACTCTGTGCTTCCTCATGCATTTGTTGGCCACAAACCCGGACGCTCAGCAGAAGCTGCACGAGGAGATTGATGCCATTTTGCCCAATAAGGCCCCTCCCACGTATGATGCTGTGTTCCAGATGGAGTATCTTGACATGGTATTGAATGAAACACTCAGGTTATATCCTATTGCTGGCCGACTGGAAAGAGTCTGTAAAAAAGATGTTGAAATCAATGGTGTGAACATTCCCAAAGGAAGTGTGGTGATGGTGCCATCTTATGTGATTCATCGAGACCCAGAACTCTGGCCAGAGGCTGAAAAATTCATTCCTGAAAGGTTCAGTAAGGAAAACAAGGACAGCATTGACCCTTACTTATACCTGCCCTTCGGAACTGGACCCCGAAACTGCATTGGCATGAGGTTTGCTCTGATGAACATGAAATTTGCTATCGTCAGAATTCTACAGGAGTTCTCCGTGAAACCTTGTAAAGAAACCCAGATTCCCATGAAAATTGGCAGAGAACCAATTCTCACACCAGCAGTGCCTGTTGTTCTAAAGTTTGAGTCCCGGGTTGGAATTGTAAATGGAGCCTGA--------------------->Elephantulus edwardii CYP3A9 XM 006889745 LOC102857952---ATGGAGCTCTTGCCCAGCCTGGGGCCGGAGACTTGGACCCTCCTAGTGGCCTGCAGCGTCCTGCTATTGCTGTATGGGATATGGCCCTATACTGTTTTTAAGAAGCTGGGCATTCCTGGACCCAGGCCCCTGCCATTCCTTGGGACATATCTGGGATGTCGAAAGGGAATTTTTCAATTTGAACTTGAATGTTTTAACAAGTATGGCAAAATATGGGGCATCTATGAAGGCAGACAGCCCGTCTTGGCCATCTTGGACGCTGCCATCATCAAGACCATTCTGGTGAAGGAGTTCTACACCCTCTTCACCAACCGCCAAAACTTCGGCTTAAATGGAGACTTGGAATCTGCTATCATCTTTGCAGAGGATGAAAGGTGGAAGTGGATCAGAGCCACGATCTCCCCGACCTTCACCAGTGGGAAGCTCAAGGAAATGTTTTCCCTCATTAACCATCATGGAGATATTTTGGTGAAAAATCTTGAGAAGAAAGTGGCTCGGGACGAGTTGGTGGACGTGAAGGAGATTTTTGGGGCCTACAGTCTGGATGTCATCACCAGCACTTCCTTTGGTGTGGACACTGACTCCATCAACAACCCTGAGGACGTTCTTCTCCAACGAATTAAGAAGCTGCTCTCCATCAATGTCTTCACCCCTATGATCTTCTTTGCAGCAATATTCCCTTTTCTGAAGCCACTGATGGAAAGGATGAATGTGACCATATTTCCACGGAAGGAGTTGAATTTTTTTGCAAGTGTAACCAAGCGTCTTAAGGAACAAAGGCAAGAAAGTGGATGCAGGGACCGTGTGGATTTTCTACAGCTGATGATTGATTCCCAGGCTGAGGTCGGCTCAGAACACAGTGAGGCTTTGACTGACGAGGAGATTTCTGCTCAAGCCATCACCTTCATTTTTGCTGGATATGAGACCTCGAGCTTAGCCCTGAGCTATGTGGCTTACAACCTGGCTACTCACCCTGAGACACAAGCGAGGCTTCAGGAGGAGATAGACCGTGCCTGCCCCAGCAAGGCAAACCCCACCTATGAGGTTCTCCTCAAGATGGAGTATCTGGACATGGTGATAAACGAGACCCTCCGGCTCTTCCCTGTGGGAGGGCGTCTGGAGAGAATGTGCAAGAAGACCATTGAGATCCACGGAGTGACCATCCCCAAGGGAACAGTGGTGATAGTCCCCACCTACGTGCTGCACCGAGACCCTGAGTACTGGCCCGAGCCGGAGGAGTTCCGGCCCGAGAGGTTCAGCAAGGAGAACGAGCGGGCGAGGGACCCCTACGTGTTCCTCCCCTTCGGGGCCGGGCCCCGGAACTGCGTGGGCATGAGGTTTGTTGTCCTCTCCATGAAGGCCGCCCTCGTCTCGCTCCTGCAGAACTTCTCCTTGGAGACCTGCACGGAGACTACAATCCCCCTGGAACTGAGCACCAACCTCCTGATGCAGCCCAAAAAACGCATCCTGCTGAAGCTGACCCCCAGGAGACGCTGCGCCTGA------------------------------>Elephas maximus indicus CYP3A8 XM 049903682 LOC126086883---ATGGACCTGATTCCAAGCTTTTCAGCGGAGACCTGGATTCTCCTGGTCACCAGCCTGGTGCTCCTCTACCTATATGGAACCCATTCACATAATGTTCTAAAGAAGCTCAGGATTCCTGGGCCCAAGCCTCTGCCTTTTGTGGGGAGTGTTCTGGCCCACCGCAAGGGTTTGTGGGATTTTGACATGAAATGTTCTAAAAAGTATGGAAAAATATGGGGGTTTTATCATGGTCTACAGCCTGTGATAGCCATCACAGATCCTGGCATGATCAAGACAATAATGGTGAAAGAATGTTATTCCACCTTCACAAACCGGAGGGTTTTTGTTCCAATGGGATTTATGAAATCTGCCATTTCCTTGTCTAAGGATGAAGAATGGAGGAGAGTACGAACGTTGCTGTCTCCAACCTTCACCAGTGGAAAACTCAAGGAGATGCTCCCCATCATCGGCCAGTATGGAGAAGTATTGTTGAAGCATCTGAGAGAGGAAGCAGAAAAAGGCAAGCCTGTCACATTGAAGAACATCTTCGGGGCTTACAGCATGGATGTGATCACAAGCACGTCATTTGGAGTGAACATCGATTCCCTCAACAACCCACAAGATCCCTTTGTGCAAAACATCAGGAAGCTCATGAGATTTAATATCTTCGACCCATTGATTTTCACAATAACAGTCTTTCCATTCCTTACTCCAATTCTTGAAGCACTAAGTATCTCTGTGTTTCCAAGAGCTGTTACTGATTTTTTTACAAAATCTGTAAAAACAATAAAAGAAAGCCGCCTTAAAGACAATAAAAAGCACCGAGTGGACTTTCTTCAGCTGATGATCGATTCCCAGAATTCCAAGGAAACTGTGTCCCATAAAGCTTTGACTGATATGGAGCTCGTAGCCCAATCAATTATCTTTATTTTTGCTGGTTATGAGACCACTAGCACTACTCTTTCCTTCCTTATGTATTTATTGGCCACCCACCCTGATATTCAGCAGAAACTGCAGAAGGAGATTGATGCGGCTTTCCCCAATAAGGCATCTCCCACGTATGATGTCATGTTGCAGATGGAATATCTTGACATGGTGGTGAATGAAACACTCAGATTATTCCCAATTGTTGGCAGAATTGAGAGGGTCTGCAAGAAAGATGTTGAAATCAGTGGAGTGACCATTCCCAAAGGGGCAGTGGCAATGGTGCCAGCCTTTGCTCTTCACCGAGACCCAGAACACTGGCCAGAGCCTGAGAAGTTCATTCCTGAGAGGTTCAGTAAGGAGAACAAGGACAGCATAGATCCTTACTTATACCTGCCCTTTGGAATTGGACCCCGAAATTGCATCGGTATGAGGTTTGCTCTCATGAACATGAAACTTGCTATCATCAGAGTTCTGCAGGAGTTCTCCGTCAAACCTTGTAAAGAAACACAGATCCCCATGAAAGTAGGCCATGGAGCAATTATTGCACCAGAAGTACCTGTTGTTATAATGTTTGAGTCAAGAGATGGAAATGCAAGTGGAGCCTGA--------------------->Elephas maximus indicus CYP3A8 XM 049903685 LOC126086884---ATGGACCTGATCCCAAACTTTTCCATGGAGACCTGGATTCTTCTGGCCACCACCCTGGTGCTCCTCTACATATGTGGAACCTATTCACATAATGTTCTAAAGAAGCTCGGGATTCCTGGGCCCAAACCTTTGCCTTTTTTGGGAACTCTTCTGGCCTACCGCAAGGGTGCGTGGGATTTTGATGTGAAATGTTCTAAAAAGTATGGAAAATTATGGGGGTTTTATGATGGTCCGCAGCCTGTGATAGCCATCACAGATCCAGGCATGATCAAGACAGTACTGGTGAAAGAAAGTTATTCCACCTTCACAAATCGGAGGATGATAGGTCCAATGGGATTTATGAAATCTGCTCTTTCCTCGTCCAAGGATGAACAGTGGAAGAGATTACGAACATTGCTATCTCCAACCTTCAGCAGTGGAAAACTCAAGGAGATGTTCCCCATCATCAGCCAATATGGTGACTTGGTGGTGAAGCATCTGAGAGAGAAAACACAGAAAGGCAAGCCTGTCACATTGAAAAGTGTCTTCGGGGCTTACAGCATGGATGTGATAACTAGCACGTCATTTGGAGTGAACATCGATTCCCTCAGCAACCCACAAGATCTCTTTGTGAAAAATGCCAGGAACCTCATAAGATTTGATTTCTTGGACCCACTTATTTTCTTAATAACACTCTTTCCATTCCTTATTCCAATTTGTGAAGCATTAAAAATCTCTGTGTTCCCAAGAGCTGCTACTGATTTTTTCACAAAATCTGTACAAAGAATTAAAGAAAGCCGCCTTAAAGATAATCAAAAGCGCCGAGTGGATTTGCTTCAGCTGATGATGGACTCCCAGGATACCAAAGAAATCTCACCCCAGAAAGCTCTGACTGATACGGAGCTCGTGGCTCAATCAATTATGTTTATTTTTGCTGGCTATGAGACCACTAGCACTGCTCTTTCCTTCGTTATGTATTTATTGGCCACACACCCTGATATTCAGGAGAAATTGCAGAGGGAGATTGATGCAGCTTTTCCCAATAAGGCACCTCCGACGTATGACGCCATGTTACAGATGGCGTATCTTGACATGGTGGTGAACGAAACACTCAGATTATTCCCAATTGCTGGCAGACTTGAGAGGGTCTGTAAGAAAGATATTGAAATCAATGGAGTGACCATTCCCAAAGGGACAATCGTGATGGTGCCAATCTTTGTTCTTCACCGAGATGCAGAACACTGGCCAGAGCCTGAGAAGTTCATGCCTGAAAGGTTCAGTAAGGAGAACAAGGACAACGTAGACCCTTACTTATATCTGCCCTTTGGAACTGGACCCCGAAACTGCATCGGCATGAGGTTTGCTCTCATGAACATGAAGCTTGCTATCATCAAAATTCTGCAAGAGTTCTCCGTCAAACCTTGTAAAGAAACACAGATCCCCTTAAAATTAGGCAGGGAAAGAATCCTGGCACCGGAAGGACCTATTGTTATAAAGTTTGAGTCAAGAGATGGACATGCAAGTGGAGCCTAA--------------------->Elephas maximus indicus CYP3A9 XM 049904229 LOC126087140---ATGGCCTTTCTGCCCAGCCTGGGAGCAGAGACCTGGGTGTTTCTGGTGACCTGTGGTGCCCTCTTGCTGCTGTATGGGATATGGCCATATAATTTTTTTAGGAAGCTGGGTATTCCTGGACCCAGGCCTCTGCCATTCATTGGGACATATTTGGAATACCGAAAGGGAATGTTAGAATTTGATCTGGAATGTTCTAAGAAATATGGCAAAATATGGGGCCTATATGAAGGCAGACAGCCTATCCTGGCCATCCTGGATCCTGATCTCATCAAGACGGTTCTTGTCAAAGAGTTCTACACCGCTTTTACCAACCGACGGAACTTGGCTTTAAGTGGAAATCTGAAATTGGCCATCACTGAGGTAGAGGATGAGATGTGGAAGCGGATTAGGGCCATTATCTCCCCAACCTTCTCCAGTGGGAAGCTCAAGGAGATGTTTCCCCTCATCAAACACCATGGAGACATTCTGATGAAAAACATTGAGAAGAAAGTGGCTCAGGATGAGGTGGTCAATGTCAGCGAGATTTTTGGAGCCTACAGTCTGGATGTCATCACCAGCACTTCCTTTGGTATAGACACTGATTCCATCAATAACCCCGATGATATTATTCTACGCTGTGTTAAGAAGGCGGTCTCCGTCAGTTTTCTGAGCCCCCTGATCTTCCTAACAGGGTTGTTCCCTTTTCTTGTACCATTGCTGGAAAGGATGAATGTGACTCTGCTTTCCAGGAAGGAGTTGGACTTCTTTGTGAATGTAACCCAGCGTCTTAAGGAGCAACGGCAAGCAAGTGGACGCAGTGACCGTGTGGATTTGCTACAGCTAATGATCGATTCCCAGGCCACAGTCAGCCTGGAATCC------GCTTTGACGGACGTAGAGATTACTGCTCAAAGTATCATCTTTATTTTTGCTGGATTTGAGACCTCAAGTTTAACCCTCAGCTTCATAGCTTATAACCTCGCCACTCACCCCGAGGTGCAAGAGAAACTTCAAGAGGAGATCGACAGTGCCTTGCCCAACAAGGAGGACTTCACCTACGATGCCCTCTTCCAGATGGAGTATCTGGACATGGTGGTGAATGAGACCCTCCGGCTCTTCCCTCTGGGGGGACGTCTGGAGAGAGTTTGCAAGAAGACTATTGAGATCAATGGTGTCACTGTCCCCAAAGGAACGGTCGTGGTCATTCCCACTTATGTTCTGCACCGCGATCCAGCATATTGGCCTGAGCCGGAGAAATTCTGTCCTGAGAGGTTCAGTAAAGACAACAAGAAGGGGTTGGACCCCTATGTGTTCCTCCCCTTTGGGATCGGGCCTCGGAACTGCATTGGCATGAGGTTCTCACTCCTTTCTCTGAAGGCAGCCCTTGTCCTGCTTCTGCAGAACTTCTCCTTGGAGATTTGCAAAGAGACTCCCATCCCCTTAGAACTGAACACCAACAGCTTCATGGTACCCAAGAAGCCTATCTTTCTGAAGCTCACACCCAGAACAAGGGTTGTGTCCCAGGAGTGAGTTGTGTCCCAGGAGTGA--->Elephas maximus indicus CYP3A21 XM 049904232 LOC126087141---ATGGCTCTTCTGCCCAGCCTTGGGGCAGAGACCTGGGTGCTCCTGGCAACCTGCGGTGCCCTCTTGCTGCTGTATGGGATATGGCCATATAATTTTTTTAAGAAGCTGGGTATTCCTGGACCCAGGCCTCTGCCATTTGTTGGGACATTTTTGGAATATCGAAAGGGAATGTTAGAATTTGACCTGGAATGTTCTAAGAAATATGGCAAAATATGGGGCATCTATCACGGCAGACAGCCTGTCCTGGCCATCACGGACCCTGTTCTCATCAAGACGGTTCTGGTCAAGGAGTTCTACACTGTTTTTACCAACCGACGGAACTTGGGTTTAAATGGAGATTTGATATCTAGCATCAGCATCGCAGATGATGAAAAGTGGAAGTGGATCAGAGCCCTTCTCTCTCCAGCCTTCAGCAGCGGGAAGCTCAAGAAAATGTTTCCCCTCATCAAACACCATGGAGACATGCTGGTGCAAAACCTTGAGAAGAAAGTATCCCGGGGTGAGGCAGTGAACATGACAGAGATTTTTGGAGCCTACAGTCTGGATGTCATCGCCAGCACTTCCTTTGGTGTGGACATTGATTCCATCAACAACCCGGATGATATTCTTCACCACCACGTTAAGAAGTTGATCTCCTTCCCTTTTAATAACCCCCTGATCTTCCTCATAGAGTTGTTCCCTTTCCTTGTGCCATTGCTGGAAAGGATGAATGTGTCTCTGCTTTCCTGGAAGGAGCATGACTTCTTTGTGAATGTAACCCAGCGTCTTAAGGAGCAACGGCAAGCAAGTGGATGCAGGGACTGTGTGGATTTGCTACAGCTAATGATCGATTCCCAGGCCACGGGCAGCCCAGAATCC------GCTTTGACAGACATGGAGATTGCTGCTCAAGTCATCACCTTTATTTTTGCTGGCTATGAGACCTCAAGCTTAACCCTCAGCTTCATATCTTACAACCTTGCTACTCATCCTGAGGTGCAAGAGAGGCTTCAAGAGGAGATAGACAGTGCCTTGCCCAACAAGGCAGACCCGACCTACGAGGTCCTCTTCCAGATGGAGTATCTGGATATGGTGGTAAATGAGACTCTACGGCTCTTCCCTCTGGGGGGACGTCTGGAGAGGGTCTGCAAGAAGACTGTTGAGATCAACGGGGTGACCATCCCCAAGGGAACGATGGTGGTCATTCCCACCTATGTTCTGAATCACAATTCTGAGTACTGGCCTGAGCCCGAGGAGTTCTGTCCTGAGAGGTTCAGTAAGGAGAACAAGAAGAGGCTGGACCCCTATGTGTTCCTCCCCTTTGGGATCGGGCCTCGGAACTGCATTGGCATGAGGTTTGCGCTCCTTGCCCTAAAAGCCGCCCTTGTCCTGCTTCTGCAGAACTTCTCCTTGGAGACATGCAAAGACACCCCAATCCCCTTAGAGCTGGACACCAACAGCTTCATGGTACCCAAGAAACCCATCTTTCTGAAGCTCACGCCCAGAATGAAGACTGTGTCCCAGGAGTGAACTGTGTCCCAGGAGTGA--->Equs caballus CYP3A12 LOC102149219 XM 023616805---ATGGACCTGATCCCAAGCTTTTCCACGGAAACCTGGGTTCTCCTGGCTACCAGCCTAGTGCTCATTTATCTATATGGGACCTATACGCATGGACTTTTTAAGAAGCTGGGAATTCCTGGGCCAACACCTCTGCCTTTTTTTGGAAATGTTCTGTCCTACCGTAAGGGTATTTGGAATTTTGACAGGAAATGTTTTAAAAAGTATGGGAAAATGTGGGGGGTTTATGATGGTAGACGGCCTGTGTTGGCTGTCACAGATCCAGACATGATCAAAACAGTACTAGTGAAAGAATGTTATTCTGTCTTCACAAACCGGCGGCCTTTTCGTCCAGTGGGGTTTATGAAAAGTGCCATCTCTCTGTCTGAGGATGAGGAATGGAAGAGAATACGAACGTTGCTGTCTCCAACCTTCACCAGTGGAAAGCTCAAGGAGATGTTCACAATCATTGGCCAGTATGGAGATGTGTTGGTGAGGAACTTGAGGAAGGAAGCAGAGAAAGGCAAACCCGTCACCTTGAAAGACATCTTTGGGGCCTACAGCATGGATGTGATTACTAGCACATCATTTGGAGTAAACATCGATTCCCTCAACAACCCGCAGGATCCCTTTGTGGAAAATAGCAACAAGCTCTTAAGATTTGATTTCCTCAATCCACTCATTCTTTTAATGGTACTCTTTCCGTTTCTTCAGCCAATTTTTGAAGTATTAAATATCTCTCTGTTTCCAAAAAGTGCTATTGATTTTTTCACAAAATCTGTAAAAAGGATGAAAGAGAGTCGCCTCAAAGATAAAGACAAGCACCGAGTTGATTTTCTTCAGCTGATGATTAACTCTCAGAATTCCAAAGAACTGGACACCCATAAAGGTCTGTCTGATCTGGAGCTCGTGGCCCAATCTATTGTCTTTATTTTTGCTGGCTATGAGACCACAAGCACTTCTCTTTCCTTCCTTTTGTATCTTTTGGCCACTCACCCTGATGTCCAGCAGAAGCTGCAGGAGGAGATTGATGCAATTTTCCCGAATAAGGCACCTCCCACCTATGATGCCCTGGTACAGATGGACTATCTTGACATGGTGTTGAATGAATCTCTCAGATTATTCCCAGTTGCTGTTAGACTTGAGAGAGTCTGTAAGAAAGATGCGGAAATCAATGGAGTGTTCATTCCCAAAGGGACAGTGGTGATGGTGCCAACCTTTAGTCTTCACCGAGCCTCAGAGTTTTGGCCTGAGCCCGAGGAGTTCCGTCCTGAAAGATTCAGTAAGAAGAACAAGGACAACATAAATCCTTGTATATACATGCCTTTTGGAAACGGACCCCGAAATTGCATCGGCATGAGGTTTGCTATGGTGAATATGAAACTTGCTCTTGTCAGAGTGCTGCAGAACTTCTCCTTCAAACCTTGTAAAGAAACACAGATTCCCCTGAAATTAGGCAATCAAGGACTTCTTCAACCGCAAAAACCCATTGTTCTAAAGGTTGAGTCCAGAGATGGGACCGTCAATGGAGCCTGA--------------------->Equus caballus cyp3A12 LOC100068123 XM 014729864---ATGAACCTGATTCCAAGCTTCTCCACAGACACCTGGGTTCTGCTGGCTACTATCCTGGGGCTCCTCTATCTATATGGGACCTATACACATGGGCTTTTTAAAAAACTAGGAATTCCTGGGCCAACACCTCTGCCTTTTTTGGGAAATGTCCTCAGCTATCGTAAGGGTTTTTGGGATTTTGACAAGACATGTTTTAAAAAGTATGGAAAAATATGGGGGTTTTACGATGGTCGACAGCCTGTGTTGGCCATCACGGATCCAGAGATGATCAAAACAGTATTAGTGAAAGAATGTTATTCCCTCTTCACAAACCGGCGGCCTGTTGGTCCAGTGGGAATTTTGAAAAGTAGCATCACTTTATCTGAGGATGAACAATGGAAAAGAATGCGAACGTTGCTGTCTCCAACTTTCACCAGTGGAAAGCTCAAGGAGATGTTCCCCACCATTAGTCAGTATGGAGATGTGTTGGTGAGGAACCTGAGGAAGGAAGCAGAGAAAGGCATGCCCATCACCTTAAAAGAGTTCTTTGGGGCCTACAGCATGGATGTGATTACTGGCATATCATTTGGAGTGAACATTGATTCCCTCAACAACCCACAACATCGCTTTGTGGAAAACTGCAAGAACATCTTAAAATTTCATTTCTTCGATCCACTCACTATCTTATTATCACTCTTGCCATTCCTTAGCCCAGTTTTGGAGGTATTGAATATACTTGTGTTTCCA------------------------------------------------------------------------CAACAAGTGGATTTTCTTCAATTGATGATTAACTCCCAGAATTCCAAAGAAATGAGCAGATATAAAGCTCTGTCTGATCTGGAGCTGGTGGTCCAATCTATTAGCTTTATTTTTGCTGGCTATGAGACTACTAGCAGTTCTCTTTCCTTCCTTACGTATGCCTTGGCCACTCACCCTGAAGTCCAGGAGAAACTGCAGGAGGAGATTGATTTGGCTTTCCCCAATAAGGCACCTCCCACCTATGATGCCCTGTTACAGATGGATTATCTTGACATGGTGTTGAATGAAAATCTCAGATTATTCCCAATTGCTGTTAGACTTGAGAGAGTCTGTAAGAAAAATGTGAAGATCAATGGGGTGTTCATTCCCAAAGGGACAGTGGTGATGGTGCCAAACTTTCTCCTTCACCGAGACCCGGAGTTCTGGCCAGAGCCTGAGGAGTTCCATCCTGAAAGGTTCAGTAAGAAGAACAAGGACAGAATAAATCCTTATGTATACTTGCCCTTTGGAACTGGACCCAGAAACTGCATTGCCATGAGATTCGCTATCATGAACATGAAACTTGCTGTTGTCAAGTTGCTGCAGAACTTCTCCTTCAAACCTTGTGAAGAAACACAGATCCCCCTGAAATTATGCAATCAAGGGCTAATGGAACCACAAAAACCCATTGTTCTAAAGGTTGAATTCAGAGACAGAACCGTGAGTGGCCCCTGA--------------------->Equus caballus cyp3A89 NM 001101651 XM 001505020---ATGGACCTGATCCCAAGCTTTTCCATGGAAACCTGGGTTCTCCTGGCTACCAGCCTGGTGCTCCTCTATCTATACGGGACCTACACTCATGGACTTTTTAAGAAGCTAGGAATTCCTGGGCCAACACCTCTGCCTTTTTTTGGAAATGTTCTGAGTTACCATAAGGGTATTTGGGATTTTGATAAGAAATGTTTTGAAAAGTATGGAAAAATGTGGGGGACTTATCATGGCACAAAACCTGTGCTGGCTATCACAGATCCAGACATGATCAAAACAGTACTAGTGAAAGAATGCTATTCTGTCTTTACAAACCGGCGGCCTTTTGGTCCATTCGGATTTATGAAAAGTGCCATCTCTCTGTCTGAGGATGAACAATGGAAGAGAATACGAACATTGCTGTCTCCAACCTTCACGAGTGGAAAGCTCAAGGAGATGTTCCCCATCCTTGGCCAGTATGGAGACGTGTTGGTTAGGAACCTGAAGAAGGAAGCAGAGAAAGGCAAGCCCATCACCTTGAAAGACATCTTTGGGGCCTACAGCATGGATGTGATTACTAGCACATCATTTGGAGTGAACATCGACTCCCTCAACAATCCACAAGATCCCTTTGTGGAAAATACCAAGAAGCTCTTCAGTTTTGATTTCCTTGATCCATTACTTCTCTCAATAACACTCTTTCCATTTCTTAATGCAGTTTTTGAAGTATTAAATGTCTTTGTGTTTCCAAAAAGTGTTACTGATTTTTTCATAAAATCTGTAAAAAGGATGAAAGAAAGTCGCCTCAAAGATAAAGAAAAGCACCGAGTTGATTTTCTTCAGCTGATGATTAACTCTCAGAATTCAAAAGAACTGGACACCCATAAAGCTCTGTCTGATCTGGAGCTCGTGGCCCAATCTATTATCTTTATTTTTGCTGGCTATGAGACCACTAGCAGTTCTCTTTCCTTCCTTATGTATTTTTTGGCCACTCACCCTGATGTCCAGCAGAAGCTGCAGGAGGAGATTGATGCGACTTTCCCCAATAAGGCTCCTCCCACCTATGATGCCCTGGTACAGATGGAGTATCTTGACATGGTGTTGAATGAATCCCTCAGGTTATTCCCAATTGCTGTTAGACTTGAGCGGGTCTGTAAGAAAGATGTGGAAATCAATGGGGTGTTCATTCCTAAAGGGACAGTGGTGATGGTGCCAACCTTTGCTCTTCACAAACACCCAGAGTTCTGGCCAGAGCCTGAGGAGTTCCGTCCTGAAAGGTTCAGTAAGGAGAACAAGGACAGCATAAATCCTTATATATACCTGCCCTTTGGAGCTGGACCTCGAAACTGCATTGGCATGAGGTTCGCTCTGATGAACATGAAACTTGCTCTTGTCAGAATGCTGCAGAACTTCTCCTTCAAACCTTGTAAAGAAACACAGATCCCCCTGAAATTAGGCAATCAAGGACTTCTTCAACCACAAAAACCCATTGTTCTAAAGGTTGAGTCCAGAGATGGGACAGTGAGTGGAGCCTGA--------------------->Equus caballus cyp3A96 NM 001146163 XM 001505023---ATGGACCTGATCCCAAGCTTTTCTGTGGAAACATGGGTTCTCCTGGCTACCAGCCTGGCTCTCCTCTATCTATATGGGATCTATACACATGGACTTTTTAAGAAGCTGGGAATTCCAGGGCCAAAACCTCTCCCTTTTTTGGGAACTCTCCTGGGCTACCGTAAGGGTTTCGGGGGTTTTGACACGGAATGTCTTAAAAAATACAGAAAAATGTGGGGGTTTTATGATGGTCGACAGCCTGTGTTGGCTATCACAGATCCAGACTTGATCAAAACTGTACTAGTGAAAGAATGTTATTCTGTCTTCACAAACCGGCGATCTTTTGGTCCAGTGGGGTTTATGAAAAATGCCATCTCTATATCTGAGGATGAACAATGGAAGAGAATACGAACTTTGCTGTCTCCAACCTTCACCAGTGGAAAGCTCAAGGAGATGTTCCCCATCATTGGCCAGTATGGAGATGTGTTGGTGAGGAACCTGAAGAAAGAAGCAGAGAAAGGCAAGCCCATTGCCTTGAAAGACATTTTTGGGGCCTACAGCATGGATGTGATTACTAGTACATCATTTGGAGTGAACATCGATTCCCTCAACAACCCACAAGATCCCTTTGTGGAAAATACAAAGAAACTCTTAAGATTTGATTTCCTTGATCCATTCATTCTCTCAATAACAATTTTTCCATTTCTTAATCCAGTTTTTGAATTATTAAATATATTTCTATTTCCAAAGAGTGTTACTGATTTTTTCACAAAATCTGTAAAAAGGATAAAAGAAAGTCGCCTCAAAGATAAAGAAAAGCAACGAGTGGATTTCCTTCAGCTGATGATTAACTCCCAAAACTCCAAAGAAATGGACACCCATAAAGCTCTGTCTGATCTGGAGCTTGTAGCCCAATCTATTATATTTATTTTTGCTGGCTATGAGCCCGTCAGCAGTTCTCTTTCTTTCCTTCTGTATCTTTTGGCCACTCACCCTGATGTCCAGCAGAAGCTGCAGGAGGAGATTGATGCAACTTTCCCCAATATGGCACCTCCCACCTATGATGCTCTGGTACAGATGGAGTATCTTGACATGGTGTTGAATGAGTCTCTCAGATTATTCCCAGTTGCTGGTAGAATTGAAAGGACCTGTAAGAAAGATGTGGAACTTGGTGGGGTATTCATTCCCAAAGGAACAGTGGTGATGGTGCCATCCTTTGCTCTTCACCGAGACACAGAGCTCTGGCCACAACCTGAGGAGTTCCATCCAGAAAGGTTCAGCAAGGAGAACAAGGACAGCATAAATCCTTATATATATATGCCCTTTGGAAATGGACCCCGAAACTGCATTGGCATGAGGTTTGCTCTGATGAACATGAAAGTTGCTGTTGTCAGAGTGCTGCAGAACTTCTCCTTCAAACCTTGTAAAGAAACACAGATTCCCCTGAAATTAGTCACTTATGGATTTCTTCAACCAGAAAAACCAATTGTTCTAAAAGTTGAGTCCAGAGCTGGGACCGTGAGTGGAGCCTGA--------------------->Equus caballus cyp3A93 NM 001190938 XM 001505019---ATGGACCTGATCCCAACCTTTTCCATGGAAACCTGGGTTCTCCTGGCTACCAGCCTAGTGCTCGTCTATCTATATGGGACCTATACACATGGACTTTTTAAGAAGCTGGGAATTCCTGGGCCAACACCTCTGCCTTTTTTTGGAAATGTTCTGTCCTACCGTAAGGGTATTTCGAATTTTGACAGGAAATGTTTTAAAAAGTATGGGAAAATGTGGGGGGTTTATGATGGTAGAAGGCCTGTGTTGGCTATCACAGACCCAGACATGATCAAAACAGTACTAGTGAAAGAATGTTATTCTGTCTTCACAAACCGGCGGCCTTTTCGTCCAGTGGGATTTATGAAAAGCGCCATCTCTCTGTCTGAGGATGAGGAATGGAAGAGAATACGAACGTTGCTGTCTCCAACCTTCACCAGTGGAAAGCTCAAGGAGATGTTCCCAATCATTGGCCAGTATGGAGATGTGTTGGTGAGGAACCTGAGGAAGGAAGCAGAGAAAGGCAAACCCGTCACCTTGAAAGACATCTTTGGGGCCTACAGCATGGATGTGATTACTAGCACATCATTTGGAGTAAACATCGATTCCCTCAACAACCCGCAGGATCCCTTTGTGGAAAATAGCAACAAGCTCTTAAGATTTGATTTCCTCAATCCACTCATTCTTTTAATGGTACTCTTTCCGTTTCTTCAGCCAATTTTTGAAGTATTAAATATCTCTCTGTTTCCAAAAAGTGCTATTGATTTTTTCACAAAATCTGTAAAAAGGATGAAAGAGAGTCGCCTCAAAGATAAAGACAAGCACCGAGTTGATTTTCTTCAGCTGATGATTAACTCTCAGAATTCCAAAGAACTGGACACCCATAAAGGTCTGTCTGATCTGGAGCTCGTGGCCCAATCTATTGTCTTTATTTTTGCTGGCTATGAGACCACAAGCACTTCTCTTTCCTTCCTTTTGTATCTTTTGGCCACTCACCCTGATGTCCAGCAGAAGCTGCAGGAGGAGATTGATGCAATTTTCCCGAATAAGGCACCTCCCACCTATGATGCCCTGGTACAGATGGACTATCTTGACATGGTGTTGAATGAATCTCTCAGATTATTCCCAGTTGCTGTTAGACTTGAGAGAGTCTGTAAGAAAGATGCGGAAATCAATGGAGTGTTCATTCCCAAAGGGACAGTGGTGATGGTGCCAACCTTTAGTCTTCACCGAGCCTCAGAGTTTTGGCCTGAGCCCGAGGAGTTCCGTCCTGAAAGATTCAGTAAGAAGAACAAGGACAACATAAATCCTTGTATATACATGCCTTTTGGAAACGGACCCCGAAATTGCATCGGCATGAGGTTTGCTATGGTGAATATGAAACTTGCTCTTGTCAGAGTGCTGCAGAACTTCTCCTTCAAACCTTGTAAAGAAACACAGATTCCCCTGAAATTAGGCAATCAAGGACTTCTTCAACCGCAAAAACCCATTGTTCTAAAGGTTGAGTCCAGAGATGGGACCGTCAATGGAGCCTGA--------------------->Equus caballus cyp3A95 NM 001190940 XM 001505022---ATGGACCTGATCCCAAGCCTTTCCATGGAAACGTGGGTTCTCCTAGCTACCAGTCTAGTGCTCCTCTATCTGTATGGGACCTATTCACATGGACATTTTAAGAAACTGGGAATTCCTGGGCCAACACCTCTGCCATTTTTTGGAACTGTTCTGGGCTACATTAAGGGTGTTTTGGCTTTTGACAAGAAATGTTTTAAGAAATATGGAAAAATGTGGGGGTTTTATGATGGTCGACAGCCCGTGTTGGCTATCACAGATCCAGACATGATCAAAACAGTACTAGTGAAAGAATGTTATTCTGTCTTCACAAACCGGCGGTCTTTTGGTCCAATGGGACTTATGAAAAATGCCATCACTATAGCTGAGGATGAAAAATGGAAGAGAATACGAACATTACTGTCTCCAACCTTCACCAGTGGGAAGCTCAAGGAGATGTTCCCTATCATTGGCCAGTATGGAGATATGTTGGTGAGGAACCTGAGGAAGGAAGCAGAGAAAGGCAAACCCATCACCATGAAAGACATCTTTGGGGCCTACAGCATGGATGTGATTACTAGCACATCATTTGGAGTGAACATAGATTCCCTCAATAACCCACAAGATCCTTTTGTGGAAAATACTAAGAAGCTCTTAAGATTTGATTTCCTTGATCCATTCTTCCTCTCAATACTATTCTTTCCATTTCTTATCCCAGTTTATGAAGCATTAAATATCTTTCTTTTTCCAAAATGTGTTACTGATTTCTTCATAAAATCTGTAAAAAAGATGAAAGAAAGTCGTCTCAAAGATAAAGCAAAGCAACGAGTGGATTTTCTTCAGCTAATGATTAACTCCCAAAATTCCAAAGAAATAGACAACCTTAAAGTTCTGTCTGATCTAGAACTTGTGGCCCAATCTATTACGTTTATCTTTGCTGGCTATGAGCCTGTTAGCACTTCTCTTTCCTTCCTTCTGTATCTTTTGGCCACTCACCCTGATGTCCAGCAGAAGCTACAGGAGGAGATTGATGCGACTTTCCCCAATAAGGCACCTCCTACCTATGATGCCCTGGTACAGATGGAATATCTTGATATGGTGTTGAATGAAACTCTCAGATTATTCCCAATTGCTGATAGACTTGAGAGGGTCTGTAAGAAAGATGTGGAAATCAATGGGGTGTTCATTCCCAAAGGGTCAACAGTGATGATGCCAATCTTTGTTCTTCACCAACACCCAGAGTTCTGGTCGGAACCTGAGGAGTTCCGTCCTGAAAGATTCAGTAAGAAGAACAAGGACAGCATAAATCCTTATACATACCTGCCCTTTGGAACTGGACCCCGAAACTGCATTGGCATGAGGTTCGCTATCGTAAACATAAAACTTGCGGTTGTCAGAGTGCTGCAGAACTTCTCCTTCAAACCTTGTAAAGAAACGCAGATTCCCCTTAAATTAGTCAGTCAAGGACTTATTCGACCGGAAAAACCCATTGTTCTGAAAGTTGAGTCGAGAGATGGGACCATGAGTGGAGCCTGA--------------------->Equus caballus cyp3A94 XM 014729835---ATGGACCTAATCCCAAGCTTTTCCATGGAAACCTGGGTTCTCCTGGCTACCAGCCTGGTGCTCCTTTATCTATATGGGACCTATACACATGGACTTTTTAAGAAGCTGGGAATTCCTGGGCCGACACCTCTACCTTTTTTGGGAACTGTTCTGGGCTACCTTAAGGGTTTCTGGGATTTTGACAAGAAATGTTTTAAAAAGTATGGAAGTATGTGGGGGATTTATGATGGCCCACAGCCTGTGTTGGCCATTACAGATCCAGATATGATCAAAACAGTACTAGTGAAAGAATGTTATTCTGTCTTCACAAACCGGCGGTCTCTTGGTCCAGTGGGATTTATGAAGAATGCCATCTCTCTGTCTGAGGATGAGCAATGGAAGAGAATAAGAACATTGCTGTCTCCAACCTTCACCAGTGGAAAGCTCAAGGAGATGTTCCCCATCATTGGCCAGTATGGAGATGTGTTGGTGAGGAACCTGAGGAAGGAAACAGAGAAAGGAAAACCTGTCACCTTGAAAAACATCTTTGGGGCCTACAGCATGGATGTGATTACTAGCACATCATTTGGAGTGAATATCGATTCCCTCAACAACCCACAAGATCCCTTTGTGGAAAATACCAAGAAGCTCTTAAGATTTAATTTCCTCAATCCATTCTTTCTCTCAATAACACTCTTTCCATTTCTTAAAGCAGTTTTTGAAGTAATACACATATATATGTTTCCAAAACGTGTTACTGATTTTTTCACAAAATCTGTAAAAAGGATGAAAGAAAGTCGCCTCAAAGATAAACAAAAGCACCGAGTGGATTTTCTTCAGCTGATGATTAACTCTCAGAATTCCAAAGAAATGGACACCCATAAAGCTCTGTCTGATCTGGAGCTTGTGGCCCAATCTATTATCTTTATTTTTGCTGGGTACGAGACCACTAGCACTTCTCTTTCTTTCCTTATGTATCTTTTGGCCACTCATCCTGATGTCCAGCAGAAGCTTCAGGATGAGATTGATGTGACTTTCCCAAATAAGGCCCTTCCCACCTATGATACCCTGTTACAGATGGAGTATCTTGACATGGTGCTGAATGAATCTCTCAGATTGTTCCCAATTGCTGGTAGACTTGAGAGGGTCTGTAAGAAAGATGTGGAAATCAATGGAGTGCTAATTCCCAAAGGGACAGTGGTGATGGTGCCAACCTTTATTCTTCATCGAGCCTCAGAGTTCTGGCCTGAGCCTGAGGAGTTCTGTCCTGAAAGATCC------------------------------------------------------------------------------------------------------------------------------------------------------------------CCCTGAAAATAGGCAATCAAGGACTTATTCAACCACAGAAACCCATTGTTCTAAAGGTTGAGTCCAGAG------ATGGTACCTGGA--------------------------->Equus caballus cyp3A97 XM 014729837 XM 014729837------------------------------------------------------------------------------------------------------------------------------------------------------------------------------------------------------------------------------------------------------------------------------------------------------------------------------------------------------ATGAAAAATGCCATCACTCGGTCTGAGGATGAACAATGGAAGAGAATACGAACTTTGCTGACGCCAACCTTCACCAGTGGAAAGCTCAAGGAGATGTTCCCCATCATTGGCCATTATGGAGATGTGTTGGTGAGGAACCTAAGGAATGAAGCAGAGAAAGGCAAACCCGTCACCTTGAAAAACATCTTTGGGGCCTACAGCATGGATGTGATTACTAGCACATCATTTGGAGTGAACATAGATTCCCTCAACAACCCACAAGATCCCTTTGTGGACAATGCTAAGAGGCTCTTAAGACTTGATTTTCTTGATCCACTCATTCTCTCAATAACTCTCTTTCCATTTCTTCGCCCAGTTTATGAAGCATTAAATATCAGTGTGTTTCCAAAAAGTGTAACTGATTTTTTCATAAAATCTGTAAAAAGGATGAAAGAAAGTCGCCTCAAAAATAAGGAAACGAACCGAGTGGATTTTCTTCAGTTGATGATTAACTCCCAGAATTCCAAAGAAATGGACACCCATAAAGCTCTGTCTGATCTCGAGCTTGTAGCCCAATCTATTGTGTTTATTTTTGCTGGCTATGAGACTACGAGCACTTCTCTCTCCTTCCTTATGTATCTTTTGGCCACTCACCCTGATGTCCAGCAGAAGCTGCAGAAGGAGATTGATGTGACTTTCCCCAATAAGGTACCTCCCACCTATGATGCCCTGCTACAGATGGACTATCTTGACATGGTGTTGAATGAATCTCTCAGATTATTCCCAGTTGCTGGTAGACTTCAGAGGATCTGTAAGAAAGATGTGGAACTCAATGGGGTGTTCATTCCCAAAAGGACACTGGTGACTGTGCCAACCTTTGTTCTTCACCGAGCCTCAGAGTTCTGGCCAGAGCCTGAAGAGTTTCGTCCTGAAAGGTTCAGTAAGGAGAACAAGGACAGCATAAATCCTTATATATACCTGCCTTTTGGAACCGGACCCCGAAACTGCATTGGCATGAGATTTGCTCTGATGAACATGAAACTTGCTGTCGTCAGAGTGCTGCAGAACTTCTCCTTCAAACCTTGTAAAGAAACAGAGATACCCATAAAATTAGGCACTGAAGCAATTGTGAAACCACAAAAGCCCATTGTTCTAAAAGTTGAGCCCAGAGATGGGACCGTGACTGGAGCCTGA--------------------->Felis catus CYP3A131 NM 001246278---ATGGACCTGATCCCAAGCTTTTTCACAGAAACCTGGCTTCTCCTGGCTACCAGCCTGGTGCTTCTCTATCTATATGGGACCCACACACATGGACTTTTTAAGAAGCTGGGAATTCCTGGGCCAAAACCTCTGCCTTTTTTGGGAACTGCTCTGGGGTACCGTCAGGGTTTTTGTGAATTTGATGAGAAATGTTTTAGAACGTATGGAAGAATGTGGGGGTTTTATGATAGGCGACAGCCAGTGTTGGCGATCACAGATCCGGACATGATCAAAACAGTACTAGTGAAAGAATGCTATTCTGTCTTCACAAACCGGCGGTCTTTTGGTCCAGTGGGATTTATGAAAGGTGCCATCTCTCTGTCTGAAGATGAACAATGGAAGAGAATACGAACAGTGCTGTCTCCAACCTTCACCAGTGGAAAACTCAAGGAGATGTTCCCCATCGTTGGCCAGTATGGAGATGTGTTGGTGAGGAACCTGAGGAAGGAGGCAGAGAAAGGCAAGCCCGTCAACTTGAAAGACATCTTGGGGGCCTACAGCATGGATGTGATTACTAGCACATCGTTTGGAGTGAACATTGATTCCCTCAACAACCCACGAGATCCCTTTGTGGAAAATACCAAGAAGCTCTTAAAATTTTCATTTCTGGATCCATTTTTCTTCTCATTATTACTCTTCCCATTCCTTACCCCAATTTTTGACCTATTAAATATCTGGCTGTTTCCAAAAAGTGTCATTAATTTTTTCACAAAATCTGTAAAAAGGATGAAGGAAAGTCGCCTCAAAGATAAACAAAAGCACCGAGTGGATTTGCTTCAGCTGATGATTAACTCCCAGAATTCCAAAGAAACGGACACCCATAAAGCTCTGTCTGATCTGGAGCTTGTGGCCCAATCTGTTATCTTTATTTTTGCTGGCTATGAGCCCACCAGCACTTCTCTTTCCTTCCTTGTGTATGAATTGGCCACTCATCCTGATGTCCAGCAAAAACTGCAGGAGGAGATTGATGCAACTTTCCCTAACAAGGCACCTCCCACTTATGATGCCCTTGTACAGATGGAGTATCTTGACATGGTGTTGAATGAAACCCTCAGATTATACCCAATCGCTGGTAGACTTGAGAGGGTCTGTAAGAGAGATGTGGAAATCAGTGGTGTGTTCATTCCCAAAGGGACAGTGGTGATGGTGCCAACCTTTACTCTTCACCGGGACCTGGATCTCTGGCCAGAGCCTGAGGAGTTCCATCCTGAAAGGTTCAGCAAGAAGAACAAGGACAGCATAAATCCTTATATATACCTGCCTTTTGGAACTGGACCCCGAAACTGCATTGGCATGAGGTTTGCGGTCATGAACATGAAACTTGCCCTTGTCACACTCCTTCAGAACTTCTCCTTCCAACCTTGTGAAGAAACACAGATCCCCCTGAAATTAAATGCTCAAGGTATTATTCAACCAGAAAAACCCATTGTTCTCAAGGTTGAGCTGAGAGATGGGAGTGTGAGTAGAGCCTGA--------------------->Felis catus CYP3A132 NM 001246271---ATGGACCTGATCCCAAGCTTTTCCATAGAAGCCTGGCTTCTTCTGGCTACCAGCCTGGTGCTCCTCTATCTATATGGGACCTACACACATGGACTTTTTAAGAAGCTCGGAATTCCTGGGCCAAAACCTCTGCCTTTTGTGGGAACGGCTCTGGGGTACCGTCAGGGCTTTTGTGCATTTGACGAGAAATGCTTTAAAACATATGGAAGAATGTGGGGGTTTTATGATGGGCGACAGCCAGTGTTGGCGATCACAGATCCGGACATGATCAAAACAGTACTAGTGAAAGAATGCTATTCTGTCTTCACAAACCGGCGGTCTCTTGGTCCAATGGGATTTATGAAAAGTGCCATCTCTCTGTCTGAAGATGAACAATGGAAGAGAATACGAACAATGCTGTCTCCAACCTTCACCAGTGGGAAGCTCAAGGAGATGTTCCCCATCGTTGGCCAGCATGGAGATGTGTTGGTGAGGAACCTGAGGAAGGAGGCAGAGAAAGGCAAGCCCGTCAACTTGAAAGACATCTTTGGGGCCTACAGCATGGATGTGATTACTGGTACCTCCTTTGGAGTGAACATTGATTCCCTCAACAACCCACAAGATCCCTTTGTGGAAAATACGAAGAATCTCTTCAAATTTTCCTTCCTTGATCCACTTTTCTTCTCAATAATACTCTTCCCATTCCTTACCCCAATTTTTGAACTATTAAATATCTGGATATTTCCAAAAAAAGTTACTGATTTTTTCACAAAATCTGTAAAAAGTATGAAGGAAAGTCGCCTCAAAGATAAACAAAAGCACCGAGTGGATTTGCTCCAGCTGATGATTAACTCCCAGAATTCCAAAGAAACAGACACCCATAAAGCTCTGTCTGATCTAGAGCTTGTGGCCCAATCTATTATGTTTATTTTTGGTGGCTATGAGACCACAAGCACTTCTCTTTCCTTCCTTGTGTATGATTTGGCCACTCACCCTGATGTCCAGCAGAAACTGCAGGAGGAGATTGATGCAACTTTCCCTGATAAGGCACCTCCCACTTATGATGCCCTTGTACAGATGGAGTATCTTGACATGGTGTTGAATGAAACCCTCAGATTATACCCAATCGCTGGTAGACTTGAGAGGGTCTGTAAGAGAGATGTGGAAATCAGTGGTGTGTTCATTCCCAAAGGGACAGTGGTGATGGTGCCAACCTTTACTCTTCACCGGGACCTGGATCTCTGGCCAGAGCCTGAGGAATTCTGTCCTGAAAGGTTCAGCAAGAAGAACAAGGATAGTATAAATCCTTATATATACCTGCCTTTTGGATCTGGACCCCGAAACTGCATTGGCATGAGGTTCGCGATTATGAACATGAAACTTGCCCTTGTCAGAGTCCTGCAGAACTTCTCCTTCAAGCCTTGTAAAGAAACACAGATCCCACTGAAATTAAACACTCTAAGTATTATTGAACCAGAAAAACCCATTGTTCTCAAGGTTGAGCTGAGAGATGGGAGTGTGAATGGAGCTTGA--------------------->Homo sapiens CYP1A1 NM 000499------ATGCTTTTCCCATCCATGTCGGCCACGGAGTTTCTTCTGGTCATCTTCTGTCTGGTATTCTGGGTAATCAGGGCCTCAAGACCTCAGGTCCCCAAAGGCCTGAAGAATCCACCAGGGCCATGGGGCTGGCCTCTGATTGGGCACATGCTGACCCTGGGAAAGAACCCGCACCTGGCACTGTCAAGGATGAGCCAGCAGTATGGGGACGTGCTGCAGATCCGAATTGGCTCCACACCCGTGGTGGTGCTGAGCGGCCTGGACACCATCCGGCAGGCCCTGGTGCGGCAGGGC---GATGATTTCAAGGGCCGGCCCGACCTCTACACCTTCACCCTCATCAGTAATGGTCAGAGCATGTCCTTCAGCCCAGACTGGGCTGCCCGCCGGCGCCTGGCCCAGAATGGCCTGAAAAGT------------------TTCTCCATTGCCTCTGACCCAGCCTCCTCAACCTCCTGCTACCTGGAAGAGCATGTGAGCAAGGAGGCTGAGGTCCTGATAAGCACGTTGTTTAACCCCTACAGGACCAATGTCATCTGTGCCATTTGCTTTGGCCGGCGCTATGACAGCCTAGTCAACCTGAATAATAATTTCGGGGAGGTGGTTGGCTCTGGAAACCCAGCTGACTTCATC---CCTATTCTT------------------CGCTACCTAAACCCTTCCCTGAATGCCTTC------------------AAGGACCTGAATGAGAAGTTCTACAGCTTCATGCAGAAGATGGTCAAGGAGCACTACAAAACCTTTGAGAAGGGCCACATCGACATCACAGACAGCCTGATTGAGCACTGTCAGGAGAAGCAGCTGGATGCCGTCCAG---CTGTCAGATGAGAAGATCATTAACATCGTCTTGGACCTCTTTGGAGCTGGGTTTGACACAGTCACAACTGCTATCTCCTGGAGCCTCATGTATTTGGTGATGAACCCCAGGGTACAGAGAAAGATCCAAGAGGAGCTAGACACAGTGATTGGCAGGTCACGGCGGCCCCGGCTCTCTGACAGATCCCATCTGCCCTATATGGAGGCCTTCATCCTGGAGACCTTCCGACACTCTTCCTTCGTCCCCACCATCCCCCACAGCACAACAAGAGACACAAGTTTGAAAGGCTTTTACATCCCCAAGGGGCGTTGTGTCTTTGTAAACCAGTGGCAGATCAACCATGACCAGAAGCTATGGGTCAACCCATCTGAGTTCCTACCTGAACGGTTTCTCACCCCTGATGGTGACAAGGTGTTAAGTGAGAAGGTGATTATCTTTGGCATGGGCAAGCGGAAGTGTATCGGTGAGACCATTGCCCGCTGGGAGGTCTTTCTCTTCCTGGCTATCCTGCTGCAACGGGTGGAATTC------------------AGCGTGCCACTGGGCGTGAAGGTGGAC------ATGACCCCCATCTATGGGCTAACCATGAAGCATGCCTGCTGTGAGCACTTCCAAATGCAGCTGCGCTCTTAG--------------->Homo sapiens CYP3A43 NM 022820---ATGGACCTGATCCCAAGCTTTTCCATGGAGACCTGGATTCTTCTGGTCACCATCCTGGTGCTCCTCTACTTATATGGAACCCATTCACATAATGTTCTAAAGAAGCTTGGGATTCCTGGGCCCAAACCTTTGCCTTTTTTGGGAACTGCTCTGGCCTACCTCAAGGGTGCGTGGGATTTTGACATGAAATGTTCTAAAAAGTATGGAAAATTATGGGGGTTTTATGATGGTCGACAGCCTGTGATAGCCATCACAGATCCAGGCATGATCAAGACAATACTGGTGAAAGAATGTTATTCCACCTTCACAAATCGGAGGATGATAGGTCCAATGGGATTTATGAAATCTGCTATTTCCTTGTCCGAGGATGAAGTGTGGAAGAGAGTACGAACATTGCTATCTCCAACTTTCACCAGTGGAAAACTCAAGGAGATGTTGCCCATCATCAGCCAATATGGTGATGTGTTGGTGAAGCATCTGAGAGAGGAAGAACAGAAAGGCAAGCCTGTTGCGTTGAAAAGCATCTTTGGAGCTTACAGCATGGATGTGATAACTAGCACGTCATTTGGAGTGAACATCGATTCCCTCCACAACCCACAAGATCCCTTTGTGCAAAACGCCAGGAAGCTCATAAAGTTTGATTTCTTGGACCCATTTGTTTTCTTAATAACACTCTTTCCATTCCTTACTCCAATTTGTGAAGCATTAAGCATCTCTCTGTTTCCAAGAGACGCTACTGATTTTTTCACAAAATCTGTACAAAGAATGAAAGAAAGCCGCCTTAAAGATAATGAAAAGCACCAAGTGGATTTGCTTCAGCTGATGATCGACTCCCAGAATTCCAAAGAAATTATGTCCCATAAAGCTTTGACTGATATGGAGCTTGTGGCTCAATCAATTATGTTTATTTTTGCTGGCTATGAGACCACTAGCACTGCTCTTTCCTTCGTTATGTATTTATTGGCCACACACCCAGATATTCAGGAGAAACTGCAGAGGGAGATTGATGCAGCTTTTCCCGATAAGGCACCTCCCACGTATGATGGCATGTTACAGATGGAGTATCTTGACATGGTGGTGAATGAAACACTCAGATTATTCCCAATCGCTGGCAGACTTGAGAGGGTCTGTAAGAAAGATGTTGAAATCAAGGGACTGATCATTCCCAAAGGGACAGTGGTGATGGTGCCAATCTTTGTTCTCCAACGAGACCCAGAACACTGGCCAGAACCTGATAAGTTCATTCCTGAAAGGTTCAGTAAGGAGAACAAGGACAACATAGATCCTTACTTATATATGCCCTTTGGAATTGGACCCCGAAAGTGCATCGGCATGAGGTTTGCCCTTATGAACATGAAACTTGCTATCATCAGAATTCTGCAGGAGTTTTCCATCAAACCTTGTAACGAAACGCAGATCCCCTTAAAATTAGGCAAGAGAGCACTTATTGAACCAGAACGACCTGTTGTTATAATGTTTGAGTCAAGAGTTGGAAAAGTAATTGGAGCCTGA--------------------->Homo sapiens CYP3A43 NM 057095---ATGGATCTCATTCCAAACTTTGCCATGGAAACATGGGTTCTTGTGGCTACCAGCCTGGTACTCCTCTATATTTATGGGACCCATTCACATAAACTTTTTAAGAAGCTGGGAATTCCTGGGCCAACCCCTCTGCCTTTTCTGGGAACTATTTTGTTCTACCTTAGGGGTCTTTGGAATTTTGACAGAGAATGTAATGAAAAATACGGAGAAATGTGGGGGCTGTATGAGGGGCAACAGCCCATGCTGGTCATCATGGATCCCGACATGATCAAAACAGTGTTAGTGAAAGAATGTTACTCTGTCTTCACAAACCAGATGCCTTTAGGTCCAATGGGATTTCTGAAAAGTGCCTTAAGTTTTGCTGAAGATGAAGAATGGAAGAGAATACGAACATTGCTATCTCCAGCTTTCACCAGTGTAAAATTCAAGGAAATGGTCCCCATCATTTCCCAATGTGGAGATATGTTGGTGAGAAGCCTGAGGCAGGAAGCAGAGAACAGCAAGTCCATCAACTTGAAAGATTTCTTTGGGGCCTACACCATGGATGTAATCACTGGCACATTATTTGGAGTGAACTTGGATTCTCTCAACAATCCACAAGATCCCTTTCTGAAAAATATGAAGAAGCTTTTAAAATTGGATTTTTTGGATCCCTTTTTACTCTTAATATCACTCTTTCCATTTCTTACCCCAGTTTTTGAAGCCCTAAATATCGGTTTGTTTCCAAAAGATGTTACCCATTTTTTAAAAAATTCCATTGAAAGGATGAAAGAAAGTCGCCTCAAAGATAAACAAAAGCATCGAGTAGATTTCTTTCAACAGATGATCGACTCCCAGAATTCCAAAGAAACAAAGTCCCATAAAGCTCTGTCTGATCTGGAGCTTGTGGCCCAGTCAATTATCATCATTTTTGCTGCCTATGACACAACTAGCACCACTCTCCCCTTCATTATGTATGAACTGGCCACTCACCCTGATGTCCAGCAGAAACTGCAGGAGGAGATTGACGCAGTTTTACCCAATAAGGCACCTGTCACCTACGATGCCCTGGTACAGATGGAGTACCTTGACATGGTGGTGAATGAAACGCTCAGATTATTCCCAGTTGTTAGTAGAGTTACGAGAGTCTGCAAGAAAGATATTGAAATCAATGGAGTGTTCATTCCCAAAGGGTTAGCAGTGATGGTTCCAATCTATGCTCTTCACCATGACCCAAAGTACTGGACAGAGCCTGAGAAGTTCTGCCCTGAAAGGTTCAGTAAGAAGAACAAGGACAGCATAGATCTTTACAGATACATACCTTTTGGAGCTGGACCCCGAAACTGCATTGGCATGAGGTTTGCTCTCACAAACATAAAACTTGCTGTCATTAGAGCACTGCAGAACTTCTCCTTCAAACCTTGTAAAGAGACTCAGATCCCACTGAAATTAGACAATCTACCAATTCTTCAACCAGAAAAACCTATTGTTCTAAAAGTGCACTTAAGAGATGGGATTACAAGTGGACCCTGA--------------------->Homo sapiens CYP3A4 NM 001202855---ATGGCTCTCATCCCAGACTTGGCCATGGAAACCTGGCTTCTCCTGGCTGTCAGCCTGGTGCTCCTCTATCTATATGGAACCCATTCACATGGACTTTTTAAGAAGCTTGGAATTCCAGGGCCCACACCTCTGCCTTTTTTGGGAAATATTTTGTCCTACCATAAGGGCTTTTGTATGTTTGACATGGAATGTCATAAAAAGTATGGAAAAGTGTGGGGCTTTTATGATGGTCAACAGCCTGTGCTGGCTATCACAGATCCTGACATGATCAAAACAGTGCTAGTGAAAGAATGTTATTCTGTCTTCACAAACCGGAGGCCTTTTGGTCCAGTGGGATTTATGAAAAGTGCCATCTCTATAGCTGAGGATGAAGAATGGAAGAGATTACGATCATTGCTGTCTCCAACCTTCACCAGTGGAAAACTCAAGGAGATGGTCCCTATCATTGCCCAGTATGGAGATGTGTTGGTGAGAAATCTGAGGCGGGAAGCAGAGACAGGCAAGCCTGTCACCTTGAAAGACGTCTTTGGGGCCTACAGCATGGATGTGATCACTAGCACATCATTTGGAGTGAACATCGACTCTCTCAACAATCCACAAGACCCCTTTGTGGAAAACACCAAGAAGCTTTTAAGATTTGATTTTTTGGATCCATTCTTTCTCTCAATAATC---TTTCCATTCCTCATCCCAATTCTTGAAGTATTAAATATCTGTGTGTTTCCAAGAGAAGTTACAAATTTTTTAAGAAAATCTGTAAAAAGGATGAAAGAAAGTCGCCTCGAAGATACACAAAAGCACCGAGTGGATTTCCTTCAGCTGATGATTGACTCTCAGAATTCAAAAGAAACTGAGTCCCACAAAGCTCTGTCCGATCTGGAGCTCGTGGCCCAATCAATTATCTTTATTTTTGCTGGCTATGAAACCACGAGCAGTGTTCTCTCCTTCATTATGTATGAACTGGCCACTCACCCTGATGTCCAGCAGAAACTGCAGGAGGAAATTGATGCAGTTTTACCCAATAAGGCACCACCCACCTATGATACTGTGCTACAGATGGAGTATCTTGACATGGTGGTGAATGAAACGCTCAGATTATTCCCAATTGCTATGAGACTTGAGAGGGTCTGCAAAAAAGATGTTGAGATCAATGGGATGTTCATTCCCAAAGGGGTGGTGGTGATGATTCCAAGCTATGCTCTTCACCGTGACCCAAAGTACTGGACAGAGCCTGAGAAGTTCCTCCCTGAAAGATTCAGCAAGAAGAACAAGGACAACATAGATCCTTACATATACACACCCTTTGGAAGTGGACCCAGAAACTGCATTGGCATGAGGTTTGCTCTCATGAACATGAAACTTGCTCTAATCAGAGTCCTTCAGAACTTCTCCTTCAAACCTTGTAAAGAAACACAGATCCCCCTGAAATTAAGCTTAGGAGGACTTCTTCAACCAGAAAAACCCGTTGTTCTAAAGGTTGAGTCAAGGGATGGCACCGTAAGTGGAGCCTGA--------------------->Homo sapiens CYP3A5 NM 000777---ATGGACCTCATCCCAAATTTGGCGGTGGAAACCTGGCTTCTCCTGGCTGTCAGCCTGGTGCTCCTCTATCTATATGGGACCCGTACACATGGACTTTTTAAGAGACTGGGAATTCCAGGGCCCACACCTCTGCCTTTGTTGGGAAATGTTTTGTCCTATCGTCAGGGTCTCTGGAAATTTGACACAGAGTGCTATAAAAAGTATGGAAAAATGTGGGGAACGTATGAAGGTCAACTCCCTGTGCTGGCCATCACAGATCCCGACGTGATCAGAACAGTGCTAGTGAAAGAATGTTATTCTGTCTTCACAAATCGAAGGTCTTTAGGCCCAGTGGGATTTATGAAAAGTGCCATCTCTTTAGCTGAGGATGAAGAATGGAAGAGAATACGGTCATTGCTGTCTCCAACCTTCACCAGCGGAAAACTCAAGGAGATGTTCCCCATCATTGCCCAGTATGGAGATGTATTGGTGAGAAACTTGAGGCGGGAAGCAGAGAAAGGCAAGCCTGTCACCTTGAAAGACATCTTTGGGGCCTACAGCATGGATGTGATTACTGGCACATCATTTGGAGTGAACATCGACTCTCTCAACAATCCACAAGACCCCTTTGTGGAGAGCACTAAGAAGTTCCTAAAATTTGGTTTCTTAGATCCATTATTTCTCTCAATAATACTCTTTCCATTCCTTACCCCAGTTTTTGAAGCATTAAATGTCTCTCTGTTTCCAAAAGATACCATAAATTTTTTAAGTAAATCTGTAAACAGAATGAAGAAAAGTCGCCTCAACGACAAACAAAAGCACCGACTAGATTTCCTTCAGCTGATGATTGACTCCCAGAATTCGAAAGAAACTGAGTCCCACAAAGCTCTGTCTGATCTGGAGCTCGCAGCCCAGTCAATAATCTTCATTTTTGCTGGCTATGAAACCACCAGCAGTGTTCTTTCCTTCACTTTATATGAACTGGCCACTCACCCTGATGTCCAGCAGAAACTGCAAAAGGAGATTGATGCAGTTTTGCCCAATAAGGCACCACCTACCTATGATGCCGTGGTACAGATGGAGTACCTTGACATGGTGGTGAATGAAACACTCAGATTATTCCCAGTTGCTATTAGACTTGAGAGGACTTGCAAGAAAGATGTTGAAATCAATGGGGTATTCATTCCCAAAGGGTCAATGGTGGTGATTCCAACTTATGCTCTTCACCATGACCCAAAGTACTGGACAGAGCCTGAGGAGTTCCGCCCTGAAAGGTTCAGTAAGAAG---AAGGACAGCATAGATCCTTACATATACACACCCTTTGGAACTGGACCCAGAAACTGCATTGGCATGAGGTTTGCTCTCATGAACATGAAACTTGCTCTAATCAGAGTCCTTCAGAACTTCTCCTTCAAACCTTGTAAAGAAACACAGATCCCCTTGAAATTAGACACGCAAGGACTTCTTCAACCAGAAAAACCCATTGTTCTAAAGGTGGATTCAAGAGATGGAACCCTAAGTGGAGAATGA--------------------->Homo sapiens CYP1A2 NM 000761---ATGGCATTGTCCCAGTCTTTCTCGGCCACAGAGCTTCTCCTGGCCTCTGCCTGCCTGGTATTCTGGGTGCTCAAGGGTTTGAGGCCTCGGGTCCCCAAAGGCCTGAAAAGTCCACCAGAGCCATGGGGCTGGCCCTTGCTCGGGCATGTGCTGACCCTGGGGAAGAACCCGCACCTGGCACTGTCAAGGATGAGCCAGCGCTACGGGGACGTCCTGCAGATCCGCATTGGCTCCACGCCCGTGCTGGTGCTGAGCCGCCTGGACACCATCCGGCAGGCCCTGGTGCGGCAGGGC---GACGATTTCAAGGGCCGGCCTGACCTCTACACCTCCACCCTCATCACTGATGGCCAGAGCTTGACCTTCAGCACAGACTGGGCTGCCCGCCGGCGCCTGGCCCAGAATGCCCTCAACACC------------------TTCTCCATCGCCTCTGACCCAGCTTCCTCATCCTCCTGCTACCTGGAGGAGCATGTGAGCAAGGAGGCTAAGGCCCTGATCAGCAGGTTGTTCGACCCTTACAATGCCAACGTCATTGGTGCCATGTGCTTCGGACAGCACTTCCCTGAGAGTAGCGATGAGATGCTCAGCCTCGTGAAGAACACTCATGAGTTCGTGGAGACTGCCTCCTCCAACCCCCTGGACTTCTTCCCCATCCTT---CGCTACCTGAACCCTGCCCTGCAGAGGTTCAAGGCC------TTCAACCAGAGGTTCCTGTGGTTCCTGCAGAAAACAGTCCAG------------GAGCACTATCAGGACTTTGACAAGAACAGTGTCGACATCACGGGTGCCCTGTTCAAGCACAGCAAGAAGGGGCCTAGAGCCAGCGGCAACCTCATCCCACAGGAGAAGATTGTCAACCTTGTCAATGACATCTTTGGAGCAGGATTTGACACAGTCACCACAGCCATCTCCTGGAGCCTCATGTACCTTGTGACCAAGCCTGAGATACAGAGGAAGATCCAGAAGGAGCTGGACACTGTGATTGGCAGGGAGCGGCGGCCCCGGCTCTCTGACAGACCCCAGCTGCCCTACTTGGAGGCCTTCATCCTGGAGACCTTCCGATTCTTGCCCTTCACC------ATCCCCCACAGCACAACAAGGGACACAACGCTGAATGGCTTCTACATCCCCAAGAAATGCTGTGTCTTCGTAAACCAGTGGCAGGTCAACCATGACCCAGAGCTGTGGGAGGACCCCTCTGAGTTCCGGCCTGAGCGGTTCCTCACCGCCGATGGCACTGCCATTAACAAGCCCTTGATGATGCTGTTTGGCATGGGCAAGCGCCGGTGTATCGGGGAAGTCCTGGCCAAGTGGGAGATCTTCCTCTTCCTGGCCATCCTGCTACAGCAACTGGAGTTC------------------AGCGTGCCGCCGGGCGTGAAAGTCGAC------CTGACCCCCATCTACGGGCTGACCATGAAGCACGCCCGCTGTGAACATGTCCAGGCGCGGCTGCGC------TTCTCCATCAATTGA>Loxodonta africana cypA21 LOC100658511 XM 010596265---ATGGCTCTTCTGCCCAGCCTTGGGGCAGAGACCTGGGTGCTCCTGGCAACCTGCGGTGCCCTCTTGCTGCTGTATGGGATATGGCCATATAATTTTTTTAGGAAGCTGGGTATTCCTGGACCCAGGCCTCTGCCATTTGTTGGGACATTTTTGGAATATCGAAAGGGAATGTTAGAATTTGACCTGGAATGTTCTAAGAAATATGGCAAAATATGGGGCATCTATCACGGCAGACAGCCTGTCCTGGCCATCACGGACCCTGTTCTCATCAAGACGGTTCTGGTCAAGGAGTTCTACACTGTTTTTACCAACCGACGGAACTTGGGTTTAAATGGAGATTTGATATCTAGCATCAGCATCGCAGATGATGAAAAGTGGAAGTGGATCAGAGCCCTTCTCTCTCCAGCCTTCAGCAGCGGGAAGCTCAAGAAAATGTTTCCCCTCATCAAACACCATGCAGACATGCTGGTGCAAAACCTTGAGAAGAAAGCC---CGGGGTGAGGCAGTGAACATGACAGAGATTTTTGGAGCCTACAGTCTGGATGTCATCGCCAGCACTTCCTTTGGTGTGGACATTGATTCCATCAACAACCCGGATGATATTCTTCACCACCACGTTAAGAAGTTGATCTCCTTCCCTTTTAATAACCCCCTGATCTTCCTCATAGAGTTGTTCCCTTTCCTTGTGCCATTGCTGGAAAGGATGGATGTGTCTCTGCTTTCCTGGAAGGAGCGTGACTTCTTTGTGAATGTAACCCAGCGTCTTAAGGAGCAACGGCAAGCAAGTGGATGCAGGGACTGTGTGGATTTGCTACAGCTAATGATCGATTCCCAGGCCACGGGCAGCCCAGAATCC------GCTTTGACAGACATGGAGATTGCTGCTCAAGTCATCACCTTTATTTTTGCTGGCTATGAGACCTCAAGCTTAACCCTCAGCTTCATATCTTACAACCTTGCTACTCATCCTGAGGTGCAAGAGAGGCTTCAAGAGGAGATAGACAGTGCCTTGCCCAACAAGGCAGACCCGACCTACGAGGTCCTCTTCCAGATGGAGTATCTGGATATGGTGGTAAATGAGACTCTACGGCTCTTCCCTCTGGGGGGACGTCTGGAGAGGGTCTGCAAGAAGACTGTTGAGATCAACGGGGTGACCATCCCCAAGGGAACGATGGTGGTCATTCCCACCTATGTTCTGAATCACAATTCTGAGTACTGGCCTGAGCCCGAGGAGTTCTGTCCTGAGAGGTTCAGTAAGGAGAACAAGAAGAGGCTGGACCCCTATGTGTTCCTCCCCTTTGGGATCGGGCCTCGGAACTGCATTGGCATGAGGTTTGCGCTCCTTGCCCTAAAAGCCGCCCTTGTCCTGCTTCTGCAGAACTTCTCCTTGGAGACATGCAAAGACACCCCAATCCCCTTAGAGCTGGACACCAACAGCTTCATGGTACCCAAGAAACCCATCTTTCTGAAGCTCACGCCCAGAATGAAGGCTGTGTCCCAGGAGTGA--------------------->Loxodonta africana cyp3A9 LOC100658793 XM 003416599---ATGGCCTTTCTGCCCAGCCTGGGAGCAGAGACCTGGGTGTTTCTGGTGACCTGTGGTGCCCTCTTGCTGCTGTATGGGATATGGCCATATAATTTTTTTAGGAAGCTGGGTATTCCTGGACCCAGGCCTCTGCCATTCATTGGGACATATTTGGAATACCGAAAGGGAATGTTAGAATTTGATCTGGAATGTTCTAAGAAATATGGCAAAATATGGGGCCTATATGAAGGCAGACAGCCTATCCTGGCCATCCTGGATCCTGATCTCATCAAGACGGTTCTTGTCAAAGAGTTCTACACCGCTTTTACCAACCGACGGAACTTGGCTTTAAGTGGAAATCTGAAATTGGCCATCACTGAGGTAGAGGATGAGATGTGGAAGCGGATTAGGGCCATTATCTCCCCAACCTTCTCCAGCGGGAAGCTCAAGGAGATGTTTCCCCTCATCAAACACCATGGAGACATTCTGATGAAAAACATTGAGAAGAAAGTGGCTCAGGATGAGGTGGTCAATGTGAGCGAGATTTTTGGAGCCTACAGTCTGGATGTCATCACCAGCACTTCCTTTGGTATAGACACTGATTCCATCAATAACCCCGATGATATTATTCTACGCTGTGTTAAGAAGGCGGTCTCCGTCAGTTTTCTGAGCCCCCTGATCTTCCTAACAGGGTTGTTCCCTTTTCTTGTACCATTGCTGGAAAGGATGAATGTGACTCTGCTTTCCAGGAAGGAGTTGGACTTCTTTGTGAATGTAACCCAGCGTCTTAAGGAGCAACGGCAAGCAAGTGGACGCAGTGACCGTGTGGATTTGCTACAGCTAATGATCGATTCCCAGGCCACAGTCAGCCTGGAA---------GCTTTGACGGACGTAGAGATTACTGCTCAAAGCATCATCTTTATTTTTGCTGGATTTGAGACCTCAAGTTTAACCCTCAGCTTCATAGCTTATAACCTCGCCACTCACCCCGAGGTGCAAGAGAAACTTCAAGAGGAGATCGACAGTGCCTTGCCCAACAAGGAGGACTTCACCTACGATGCCCTCTTCCAGATGGAGTATCTGGACATGGTGGTGAATGAGACCCTCCGGCTCTTCCCTCTGGGGGGACGTCTGGAGAGAGTTTGCAAGAAGACTATTGAGATCAATGGTGTCACTGTCCCCAAAGGAACGGTCGTGGTCATTCCCACTTATGTTCTGCACCGCGATCCAGCATATTGGCCTGAGCCGGAGAAATTCTGTCCTGAGAGGTTCAGTAAAGACAACAAGAAGGGGTTGGACCCCTATGTGTTCCTCCCCTTTGGGATCGGGCCTCGGAACTGCATTGGCATGAGGTTCTCACTCCTTTCTCTGAAGGCAGCCCTTGTCCTGCTTCTGCAGAACTTCTCCTTGGAGATTTGCAAAGAGACTCCCATCCCCTTAGAACTGAACACCAACAGCTTCATGGTACCCAAGAAGCCTATCTTTCTGAAGCTCACACCCAGAACAAGGGTTGTGTCCCAGGAGTGA--------------------->Loxodonta africana cyp3A4 LOC100659080 XM 02355593---------------------------ATGAATGTATGGTCTCTCAGAAGT---------------------------------------------------------------------GGCCCCAGAGCAATTAGATTAATCAGCTCTGTTTCCCGACATAGG---------------------------------------------------------TTTTATGATGGTCCGCAGCCTGTGATAGCCATCACAGATCCAGGCATGATCAAGACAGTACTGGTGAAAGAAAGTTATTCCACCTTCACAAATCGGAGGATGTTAGGTCCAACGGGATTTATGAAATCTGCTCTTTCCTCGTCCAAGGATGAACAGTGGAAGAGATTACGAACATTGCTATCTCCAACCTTCAGCAGTGGAAAACTCAAGGAGATGTTCCCCATCATCAGCCAATATGGTGACTTGGTGGTGAAGCATCTGAGAGAGAAAACACAGAAAGGCAAGCCTGTCACATTGAAAAGTGTCTTCGGGGCTTACAGCATGGATGTGATAACTAGCACGTCATTTGGAGTGAACATCGATTCCCTCAGCAACCCACAAGATCTCTTTGTGAAAAATGCCAGGAACCTCATAAGATTTGATTTCTTGGACCCACTTATTTTCTTAATAACACTCTTTCCATTCCTTATTCCAATTTGTGAAGCATTAAAAATCTCTGTGTTCCCAAGAGCTGCTACTGATTTTTTCACAAAATCTGTACAAAGAATTAAAGAAAGCCGCCTTAAAGATAATCAAAAGCGCCGAGTGGATTTGCTTCAGCTGATGATGGACTCCCAGGAGACCAAAGAAATCTCACCCCAGAAAGCTCTGACTGATACGGAGCTCGTGGCTCAATCAATTATGTTTATTTTTGCTGGCTATGAGACCACTAGCACTGCTCTTTCCTTCGTTATGTATTTATTGGCCACACACCCTGATATTCAGGAGAAACTGCAGAGGGAGATTGATGCAGCTTTTCCCAATAAGGCACCTCCGACGTATGACGCCACGTTACAGATGGCGTATCTTGACATGGTGGTGAACGAAACACTCAGATTATTCCCAATCGCTGGCAGACTTGAGAGGGTCTGTAAGAAAGATATTGAAATCAATGGAGTGACCATTCCCAAAGGGACAATCGTGATGGTGCCAATCTTTGTTCTTCACCGAGACGCAGAACACTGGCCAGAGCCTGAGAAGTTCATGCCTGAAAGGTTCAGTAAGGAGAACAAGGACAACGTAGATCCTTACTTATATCTGCCCTTTGGAACTGGACCCCGAAACTGCATCGGCATGAGGTTTGCTCTCATGAACATGAAGCTTGCTATCATCAAAATTCTGCAAGAGTTCTCCGTCAAACCTTGTAAAGAAACACAGATCCCCTTAAAATTAGGCAGGGAAAGAATCCTGGCACCGGAAGGACCTATTGTTATAAAGTTTGAGTCAAGAGATGGACATGCAAGTGGAGCCTAA--------------------->Loxodonta africana cyp3A8 LOC100677295 XM 023555932---ATGGACCTGATTCCAAGCTTTTCAGCGGAGACCTGGATTCTCCTGGTCACCAGCCTGGTGCTCCTCTACCTATATGGAACCCATTCACATAATGTTCTAAAGAAGCTCAGGATTCCTGGGCCCAAGCCTCTGCCTTTTGTGGGGAGCGTTCTGGCCCACCGCAAGGGTTTGTGGGATTTTGACATGAAATGTTCTAAAAAGTATGGAAAAATATGGGGGTTTTATCATGGTCTACAGCCTGTGATAGCCATCACAGATCCTGGCATGATCAAGACAATAATGGTGAAAGAATGTTATTCCACCTTCACAAACCGGAGGGTTTTTGTTCCAATGGGATTTATGAAATCTGCCATTTCCTTGTCTAAGGATGAAGAATGGAGGAGAGTACGAACGTTGCTGTCTCCAACCTTCACCAGTGGAAAACTCAAGGAGATGCTCCCCATCATCGGCCAGTATGGAGAAGTATTGTTGAAGCATCTGAGAGAGGAAGCAGAAAAAGGCAAGCCTGTCACATTGAAGAACATCTTCGGGGCTTACAGCATGGATGTGATCACAAGCACGTCATTTGGAGTGAACATCGATTCCCTCAACAACCCACAAGATCCCTTTGTGCAAAACATCAGGAAGCTCATGAGATTTAATATCTTCGACCCATTGATTTTCACAATAACAGTCTTTCCATTCCTTACTCCAATTCTTGAAGCACTAAGTATCTCTGTGTTTCCAAGAGCTGTTACTGATTTTTTTACAAAATCTGTAAAAACAATAAAAGAAAGCCGCCTTAAAGACAATAAAAAGCACCGAGTGGACTTTCTTCAGCTGATGATCGATTCCCAGAATTCCAAGGAAACTATGTCCCATAAAGCTTTGACTGATATGGAGCTCGTAGCCCAATCAATTATCTTTATTTTTGCTGGTTATGAGACCACTAGCACTACTCTTTCCTTCCTTATGTATTTATTGGCCACCCACCCTGATATTCAGCAGAAACTGCAGAAGGAGATTGATGCGGCTTTCCCCAATAAGGCATCTCCCACATATGATGTCATGTTGCAGATGGAATATCTTGACATGGTGGTGAATGAAACACTCAGATTATTCCCAATTGTTGGCAGAATTGAGAGGGTCTGCAAGAAAGATGTTGAAATCAGTGGAGTGACCATTCCCAAAGGGGCAGTGGCAATGGTGCCAGCCTTTGCTCTTCACCGAGACCCAGAACACTGGCCAGAGCCTGAGAAGTTCATTCCTGAGAGGTTCAGTAAGGAGAACAAGGACAGCATAGATCCTTACTTATACCTGCCCTTTGGAATTGGACCCCGAAATTGCATCGGTATGAGGTTTGCTCTCATGAACATGAAACTTGCTATCATCAGAGTTCTGCAGGAGTTCTCCGTCAAACCTTGTAAAGAAACACAGATCCCCATAAAAGTAGGCCATGGAGCAATTATTGCACCAGAAGTACCTGTTGTTATAATGTTTGAGTCAAGAGACGGAAATGCAAGTGGAGCCTGA--------------------->Loxodonta africana cyp3A12 LOC100677576 XM 023555935---ATGGACCTGATCCCAAGCTTTTCCATGGAGACCTGGATTCTTCTGGTCACCATCCTGGTACTCCTCTACTTATATGGAACCCATTCACATAATGTTCTAAAGAAGCTTGGGATTCCTGGGCCCAAACCTTTGCCTTTTTTGGGAACTTCTCTGGCCTACCTCAAGGGTTCGTGGGATTTTGACATGAAATGTTCTAAAAAGTATGGAAAATTTTGGGGGTTTTATGATGGTCGACAGCCTGTGATAGCCATCACAGATCCAGTCATGATCAAGACAATACTGGTGAAAGAATGTTATTCCACCTTCACAAATCGGAGGATGATAGGTCCAATGGGATTTATGAAATCTGCTATTTCCTTGTCCGAGGATGAAGTGTGGAAGAGAGTACGAACATTGCTGTCTCCAACTTTCACCAGTGGAAAACTCAAGGAGATGTTCCCCATCATCAGCCAATATGGTGATGTGTTGGTGAAGCATCTGAGAGAGGAAGAACAGAAAGGCAAGCCTGTTGCGTTGAAAAGCATCTTTGGAGCTTACAGCATGGATGTGATAACTAGCACGTCATTTGGAGTGAACATCGATTCCCTCCACAACCCACAAGATCCCTTTGTGCAAAACGCCAGGAAGCTCATAAAGTTTGATTTCTTGGACCCATTTGTTTTCTTAATAACACTCTTTCCATTCCTTACTCCAATTTGTGAAGCATTAAACATCTCTCTGTTTCCAAGAGACGCTACTGATTTTTTCACAAAATCTGTACAAAGAATGAAAGAAAGCCGCCTTAAAGATAATGAAAAGCACCAAGTGGATTTGCTTCAGCTGATGATCGACTCCCAGAATTCCAAAGAAATTATGTCCCATAAAGCTTTGACTGATATGGAGCTTGTGGCTCAATCAATTATGTTTATTTTTGCTGGCTATGAGACCACTAGCACTACTCTTTCCTTCGTTATGTATTTATTGGCCACACACCCAGATATTCAGGAGAAACTGCAGAGGGAGATTGATGCAGCTTTTCCCGATAAGGCACCTCCCACGTATGATGGCATGTTACAGATGGAGTATCTTGACATGGTGGTGAATGAAACACTCAGATTATTCCCAATCGCTGGCAGACTTGAGAGGGTCTGTAAGAAAGATGTTGAAATCAAGGGACTGATCATTCCCAAAGGGACAGTGATGATGGTGCCAATCTTTGTTCTCCAACGAGACCCAGAACACTGGCCAGAACCTGATAAGTTCATTCCTGAAAGGTTCAGTAAGGAGAACAAGGACAACATAGATCCTTACTTATATATGCCCTTTGGAATTGGACCCCGAAACTGCATCGGCATGAGGTTTGCCCTTATGAACATGAAACTTGCTATCATCAGAATTCTGCAGGAGTTTTCCATCAAACCTTGTAACGAAACGCAGATCCCCTTAAAATTAGGCAAGAGAGCACTTATTGCACCAGAACGACCTGTTGTTATAATGTTTGATTCAAGAGTTGGAAAAGTAATTGGAGCCTGA--------------------->Orycteropus afer afer cyp3A8 LOC103197263 XM 007941162---ATGGACCTGATCCCAAGCTTTTCCGGGGAGACCTGGGTCCTCCTGGCCACCAGCCTGCTGCTCCTCTACATATATGGAACCTATTCACATAATGTTCTAAAGAAGCTCGGGATTCCAGGACCCAAGCCTCTGCCTTTTGTGGGAACTGTCTTGGCCTACCGTAAGGGTATGTGGGATTTTGACATGAAATCTTCTAAGAAGTATGGAAAATTATGGGGGTTTTATGATGGTCACCAGCCTGTGATAGCTATCACAGATCCAGGCATGATCAAGACCGTAATGGTGAAAGAATGTTATTCCATCTTCACAAACCGGAGGGCTGTTGGTCCATCGGGATTTATGAAACATTCCATTTCCATTGCTGAGGATGAAGAGTGGAAGAGACTACGAACATTGCTGTCTCCAACCTTCACCAGTGGGAAACTCAAAGAGATGTTCCCCATCATTTGCCAGTATGGAGATATATTGGTGAAGCATCTGAGAGAGGAAGCAGAAAAAAGCAAGCCTGTCACGTTGAAAAGCATATTTGGGGCCTACAGCATGGATGTCATCACTGGCACATCATTTGGAGTGAACATTGATTCCCTCAACAACCCACAAGATCCCTTTGTGCAAAAAGTCAGGAAGCTCATAAAATTCGATTTCCTGGACCCACTGATGTTCTCAATAATAATGTTCCCATTTCTTACACCAATTCTTGAAGCTTTAAGCATCTCTCTGTTTCCAAAAGATACTACTGACTTTCTCACAAAATCTGTGCAAAGAATGAAAGAACAGCGCCTTAAAGAAAAGCAAAAGCACCGAATGGATCTTCTTCAGCTGATGATTGACTCCCAGAATTCCAAAGACACTGAGTCCCATAAAGCTTTGACTGACACGGAGCTGGTAGCTCAGTCAATTATCTTTATTTTTGCTGGCTACGAGACCACTAGCACTACTCTTTCCTTCCTTATGTATCTATTGGCCACACATCCTGATATTCAGCAGAAGCTTCAAAAGGAGATTGATGAGGCTTTGCCCAATAAGGCACCTCCCACTTATGACACTGTGGTACAGATGGAGTATCTTGACATGGTGGTGAATGAAACACTGAGATTATTTCCAGTCGCTCCCAGACTTGAGAGGGTCTGTAAGAAAGATGTTGAAATCAATGGAGTGTTCATTCCCAAAGGAGCATCGGTGATGGTGCCAGTCTATGTTCTTCACCGAGACCCAGAACTCTGGCCAGAGCCTGAGAAGTTCCTTCCTGAAAGGTTCAGTAAAGAGAACAAGAGCAACATAGATCCTTACTTATATCTGCCCTTTGGAACTGGACCCCGAAACTGCATCGGCATGAGGTTTGCTCTCATGAACATGAAACTTGCTGTCACCAGAGTTATGCAGGAGTTCTCTGTCAAACCTTGTAAAGAAACACAGATCCCGATACAATTAGGCAGGGAACCACTGATTTCACCGAAGGTACCTGTTGTTATAAAGTTTGAATTAAGAGATGGAAATGCAAGTGGAGCATGA--------------------->Orycteropus afer afer cyp3A24 LOC103198683 XM 007942868ATGCTTACCTTCTACCATTCCTTTGCCATGAGCCCCAGAGTGAATTTAATTGTCTGTATCACCCTTCAGTTGCTGTATGGAATATGGCCGTATAACTTGTTTAAGAAGCTGGGTATCCCTGGACCCAGGCCTCTGCCTTTCTTTGGGACATTTTTGGAATATCGAAAGGGACTTTTTGAATTTGACCTGGAATGTTCTAAGAAATATGGCAAAATGTGGGGCTTCTTTGAAGGCAGACAGCCCCTCCTGGCCATCCTGGATCCTGCCCTCATCAAGACGGTTCTGGTCAAGGAGTGCTACACCCTTTTTACCAACCGAAGGAACTTCGGTTTAAATGGAGATTTGGACTCAGGCCTCATCACCGCAGAAGATGAGAAGTGGAAGTGGATGAGATCCGTTATCTCCCCAACCTTCACCAGTGGGAAGCTCAAGGAGATGTTTCCCCTCATCAAACAACACGGAGACATTCTGGTGCGAAACATTGAGAAGAGAGCGGCTCGGGACGAGGCGGTGAACATGAAGGAGATTTTTGGAGCCTATACCCTGGATATCATCACCAGCACTTTCTTTGGTGTCCACACTGATTCCATCAATAACCCAGATGATATTATTCTTCACCAACTTAAGAAACTGATGTCCTTCAGTGTTCTGAGCCCTTTGATGATCCTTATAGTGATATTTCCTTTCTTTGTGCCACTGCTCGAAAGCATGAATGTGACTCTAGCTCCCCGGAAGCAGATGGACTTCTTTGTGAATGTGACCAAGCGTATTAAGAAGGAACGGCAAAGGAGTGGATGCAGGGACCGTGTGGATTTTCTACAGCTGATGATCGATTCGCAGGCCACCGTCAGCTCAGAACCC------GCTTTGACAGACCGGGAGATTTGCGCTCAAGCTGTCACCTTTCTTATTGCTGGATATGAGACCTCAAGCTCAACCCTCGCCTTTATAGCTTACAACCTGGCCACTCACCCGCAGGTGCAAGCAAAACTTCAAGAGGAGATCGACAGTGCCTTGCCCAACAAGGTGATGTGA------------------------------------------------------------------------------------------------------------------------------------------------------------------------------------------------------------------------------------------------------------------------------------------------------------------------------------------------------------------------------------------------------------------------------------------------------------------------------------------------------------------>Rattus norvegicus Cyp3a18 NM 145782---ATGGAGATCATTCCCAACCTTTCTATAGAGACCTGGGTGCTTCTAGCTACTAGCTTGATGCTCTTCTACATATATGGGACCTATTCTCATGGCCTGTTTAAGAAACTAGGAATTCCTGGACCCAAACCTGTGCCTTTATTTGGCACCATTTTCAACTACGGTGATGGCATGTGGAAATTTGATGATGACTGCTATAAAAAGTATGGAAAAATATGGGGGTTTTATGAGGGCCCACAGCCTTTTTTGGCTATCATGGATCCAGAGATCATCAAAATGGTGCTGGTGAAAGAATGTTACTCAGTCTTCACAAACCGTCGGTGTTTTGGGCCAATGGGATTTATGAAAAAGGCCATTACCATGTCTGAGGATGAAGAATGGAAGAGACTTCGAACAATCCTGTCTCCAACCTTCACCAGTGGCAAACTCAAGGAGATGTTCCCCCTCATGAGACAGTATGGAGATACATTGTTGAAGAACTTGAGGCGAGAAGAAGCAAAAGGGGAGCCCATCAACATGAAAGACATCTTTGGAGCTTATAGCATGGACGTGATCACTGGCACATCATTTGGAGTGAACGTCGATTCCCTCAACAATCCACAGGATCCCTTCGTGCAGAAAGCCAAGAAGATCTTAAAATTTCAAATTTTTGATCCATTTCTTCTCTCTGTAGTTCTGTTTCCATTTCTTACTCCAATATATGAGATGTTAAATTTTTCAATTTTTCCAAGACAGTCAATGAACTTTTTCAAAAAATTCGTAAAAACAATGAAGAAAAATCGCCTTGATTCAAACCAGAAGAGCCGAGTGGATTTTCTTCAACTGATGATGAATACTCAGAACTCCAAAGGCCAAGAGTCCCAGAAAGCTCTTTCTGATCTAGAAATGGCAGCACAAGCTATTATTTTCATTTTTGGGGGTTATGATGCCACAAGCACCTCCATTTCCTTCATAATGTATGAACTGGCCACTCGCCCCAATGTGCAAAAGAAACTCCAGAATGAGATTGATAGAGCTCTGCCCAATAAGGCACCTGTCACCTATGATGCTCTGATGGAAATGGAGTACCTGGACATGGTGGTGAATGAAAGTCTAAGATTGTACCCAATTGCTACCAGGCTAGACAGAGTCTCAAAAAAGGATGTGGAAATCAATGGAGTTTTTATTCCCAAAGGGACTGTAGTTACGATACCAATCTATCCTCTTCATCGGAACCCTGAGTACTGGCTAGAGCCTGAGGAATTCAACCCTGAAAGGTTCAGCAAGGAGAACAAGGGCAGCATTGATCCTTATGTATATCTGCCCTTTGGAAATGGACCCAGGAACTGCATTGGCATGAGGTTTGCTCTCATCAGCATGAAACTTGCTGTCATAGGAGTCCTGCAGAACTTCAATATCCAGCCTTGTGAGAAGACACAGATCCCTCTGAAGATCAGTAGGCAACCAATTTTCCAACCAGAAGGACCCATCATCCTAAAGCTTGTGTCAAGAGATTAA--------------------------------------->Rattus norvegicus Cyp3a23-3a1 NM 013105---ATGGACCTGCTTTCAGCTCTCACACTGGAAACCTGGGTCCTCCTGGCAGTCGTCCTGGTGCTCCTCTACGGATTTGGGACCCGCACACATGGACTTTTCAAGAAACAGGGGATTCCTGGGCCCAAACCTCTGCCTTTTTTTGGCACTGTGCTGAATTACTATATGGGTTTATGGAAATTCGATGTGGAGTGCCATAAAAAGTATGGAAAAATATGGGGGTTGTTTGATGGTCAAATGCCTCTGTTTGCCATCACGGACACAGAAATGATCAAGAATGTGCTAGTGAAGGAATGCTTTTCTGTCTTCACAAACCGGCGGGATTTTGGCCCAGTGGGGATTATGGGGAAAGCCATCTCTGTATCTAAGGATGAGGAGTGGAAGAGATATAGAGCCTTGCTGTCACCCACGTTCACCAGTGGAAGACTCAAGGAGATGTTCCCTGTCATCGAACAGTATGGAGACATTTTGGTAAAATACTTGAGGCAAGAG------AAAGGCAAACCTGTCCCTGTGAAAGAAGTGTTTGGTGCCTACAGCATGGATGTGATCACCAGCACATCATTTGGAGTGAATGTTGATTCCCTCAACAACCCGAAGGATCCTTTTGTGGAGAAAGCCAAGAAGCTCTTAAGAATTGATTTTTTTGATCCGTTGTTCTTGTCAGTAGTACTCTTTCCATTCCTCACGCCAGTATATGAGATGTTAAACATCTGCATGTTCCCAAAAGATTCAATAGAATTTTTCAAAAAATTTGTGTACAGAATGAAGGAAACCCGCCTGGATTCTGTGCAGAAGCATCGAGTGGATTTTCTTCAGCTGATGATGAATGCTCATAATTCTAAAGACAAAGAATCTCATACAGCCCTATCCGATATGGAGATCACAGCCCAGTCAATCATTTTTATTTTTGCTGGATATGAACCCACCAGCAGCACACTTTCCTTTGTCCTGCATTCCCTGGCCACTCACCCAGATACACAGAAGAAACTGCAGGAGGAGATCGACAGGGCTCTGCCCAATAAGGCACCTCCCACCTATGATACTGTGATGGAAATGGAATACCTGGATATGGTGTTGAATGAAACCCTCAGATTGTATCCAATTGGTAATAGACTTGAGAGAGTCTGTAAAAAAGATGTTGAAATCAATGGTGTGTTTATGCCCAAAGGGTCAGTGGTCATGATTCCATCTTATGCTCTTCACCGTGATCCACAGCACTGGCCAGAGCCTGAGGAATTTCGCCCAGAAAGGTTCAGCAAGGAGAACAAGGGCAGCATTGATCCTTATGTATATCTGCCCTTTGGAAATGGACCCAGGAACTGCATTGGCATGAGGTTTGCTCTCATGAATATGAAACTCGCTCTCACTAAAGTTCTGCAAAACTTCTCCTTCCAGCCTTGTAAGGAAACACAGATACCTCTGAAATTAAGCAGACAAGGACTTCTTCAACCAACAAAACCCATTATTCTAAAGGTTGTGCCACGGGATGAAATCATAACTGGATCATGA--------------------->Rattus norvegicus Cyp3a2 NM 153312---ATGGACCTGCTTTCAGCTCTCACACTGGAAACCTGGGTCCTCCTGGCAGTCATCCTGGTGCTTCTCTACCGACTTGGAACCCATAGACATGGAATTTTTAAGAAACAAGGAATTCCTGGGCCAAAACCTCTGCCTTTTTTAGGCACTGTGCTGAATTACTACAAGGGCTTAGGGAGATTTGACATGGAGTGCTATAAAAAGTATGGAAAAATATGGGGGTTGTTTGATGGTCAAACGCCTGTGTTTGCCATCATGGACACAGAGATGATTAAGAATGTACTAGTGAAGGAATGCTTTTCTGTCTTCACAAACCGGCGGGATTTTGGCCCAGTGGGGATTATGGGGAAAGCTGTCTCTGTAGCTAAGGATGAGGAGTGGAAGAGATATAGAGCCTTGCTGTCCCCCACGTTCACCAGTGGAAGACTCAAGGAGATGTTCCCCATCATTGAACAGTATGGAGACATTTTGGTAAAGTACTTGAAGCAAGAGGCGGAGACAGGCAAGCCTGTCACCATGAAAAAAGTGTTTGGTGCCTACAGCATGGATGTGATCACCAGCACATCATTTGGAGTGAACGTCGATTCCCTTAACAACCCAAAGGATCCTTTTGTGGAGAAAACCAAGAAATTGTTAAGATTTGACTTTTTTGATCCGTTGTTCTTGTCAGTAGTACTCTTTCCATTCCTCACCCCAATATATGAGATGCTAAATATCTGCATGTTCCCAAAGGATTCAATAGCATTTTTCCAAAAATTTGTGCACAGAATAAAGGAAACCCGTCTGGATTCTAAGCATAAGCACCGAGTGGATTTTCTCCAGCTGATGCTGAACGCTCATAATTCCAAAGACGAAGTGTCTCATAAAGCCCTGTCTGATGTTGAAATCATAGCCCAGTCAGTTATCTTTATTTTTGCTGGCTATGAAACCACCAGCAGCACACTCTCCTTTGTCTTGTATTTCCTGGCCACTCACCCTGATATTCAGAAGAAACTGCAGGAGGAGATCGATGGGGCCCTGCCGAGTAAGGCACCTCCTACCTACGATATTGTGATGGAAATGGAATACCTGGATATGGTGTTGAATGAAACTCTCAGATTGTATCCAATTGGTAATAGACTTGAGAGAGTCTGTAAAAAAGATATAGAACTTGATGGTTTGTTTATACCCAAAGGGTCAGTAGTGACGATTCCAACATATGCTCTTCATCATGACCCACAGCACTGGCCAAAGCCTGAGGAATTTCATCCAGAAAGGTTTAGCAAGGAGAACAAGGGAAGCATTGATCCTTATGTATATCTGCCCTTTGGAAATGGACCCAGGAACTGCATTGGCATGAGGTTTGCTCTCATGAATATGAAACTCGCTCTCACTAAAGTTCTGCAAAACTTCTCCTTCCAGCCTTGCAAGGAAACACAGATACCTCTGAAATTGAGTAGACAAGCAATTCTTGAACCAGAAAAACCCATTGTTCTAAAGGTTCTGCCACGGGATGCAGTCATAAATGGAGCCTGA--------------------->Rattus norvegicus Cyp3a62 NM 001024232---ATGGACCTGATCCCAAACATTTCCTTGGAAACCTGGATGCTCCTGGCTACCATCCTCGTGCTCCTGTATCTGTATGGAACCTCCACACATGGAAATTTTAAGAAATTAGGGATTTCTGGACCTAAACCACTGCCTTTTGTGGGAAATATTCTTGCATACCGCCATGGATTTTGGGAGTTTGATAGACACTGCCATAAAAAATATGGGGATATATGGGGGTTTTATGAGGGACGACAACCTATTTTGGCTATAACAGATCCAGATATAATAAAAACAGTGCTGGTAAAGGAATGTTATTCTACCTTCACAAACCGTCGGAGCTTTGGTCCAGCGGGAATTTTGAAAAAAGCTATCACCTTATCTGAGGATGAAGAATGGAAGAGATTAAGAACATTGCTATCTCCCACCTTCACCAGTGGGAAGCTCAAGGAGATGTTCCCCATCATTAACCAGTATGCAGATTTGCTGGTGAAAAACGTGAAACATGAAGCAGAAAAAGGCAATCCTATCACCATGAAAGACATCTTTGGGGCCTATAGCATGGATGTGATCACAGGCACATCATTTGGAGTAAACGTGGATTCCCTCAACAACCCACAGAATCCCTTTGTACAAAAAGTGAAGAAACTCTTAAAATTTAATTTCTTGGACCCATTCTTTCTCTCAGTGATACTCTTTCCATTCCTTACCCCAGTTTTTGAAGCATTTGACATAACTGTGTTTCCAAAAGATGTTATGAAGTTTTTTAGGACTTCTGTAGAACGAATGAAAGAGAACCGCATGCAAGAGAAAGTAAAGCAAAGATTAGATTTTCTTCAACTGATGATAAACTCCCAAAGTTCTGGAGATAAAGAGTCTCACCAAGGTTTAACTGATGTGGAGATTGTGGCTCAGTCCATTTTCTTTATTTTTGCCGGCTATGAGACCACTAGCAGTGCTCTTTCCTTTGCTTTGTATTTGCTGGCCACACACCCTGATCTCCAGAAGAAACTGCAGGATGAAATTGATGCAGCTCTGCCCAATAAGGCACCTGTGACCTATGATGTCCTGGTAGAGATGGAGTACTTGGACATGGTGTTAAATGAAACTCTCAGATTATTTCCAGTTGGTGGAAGACTCGAGAGGGTCTGTAAGAAAGATGTTGAAATCAATGGGGTTTTTATACCCAAAGGGACTGTGGTGATGGTACCAACCTTTGCTCTTCACAAAGACCCAAAGTGCTGGCCAGAGCCTGAGGAATTTTGTCCTGAAAGGTTCAGAAAGAAGAATCAGGACAGCATCAATCCTTACATATACCTGCCCTTTGGTAATGGACCCAGGAACTGTATTGGCATGAGGTTTGCTCTCATGAACATGAAAATTGCTCTTGTCAGAGTCCTGCAGAACTTCTCCTTTGGACTTTGTAAAGAAACTCAGATTCCTTTAAAACTAAGAAAGAAAGGATTTTTTCAACCAGAAAAACCCATCATTCTAAGGGCTGTATCAAGAGATTGA--------------------------------------->Rattus norvegicus Cyp3a73 XM 039089947---------------------------------------------------------------------------------------------------------------------------------------------------------------------------------------------------------------------------------------------------------------------------------------------------------------------------------------------------------------------------------------------------------------------------------------------------------------------------------------------------------------------------------------------------------------ATGGATGTTATTACCAGCACATCATTTGAAGTTAACATCAATTCCATCAACAACCCAAAGGATCCTTTTGTGGAGAAAGTCAAGAAATTCCAAAGATTTGACTTTTTTGATCCGTTGTTCTTGTCAGTAGTACTCTTTCCATTCCTCACCCCCATATATGAGATGTTAAATATCTGCCTCTTCCCAAAGGATTCAGTAGCATTTTTCCAAAAATTTGTGTACAGAATGAAGCAAACCCGCCTGGATTCTAAGCATAAGCACCGAGTGGATTTTCTTCAGCTGATGATGAATGCTCATAATTCCAAAGACAAAGTGTCTCATAAAGCCCTGTCTGACATTGAAATTGTAGCCCAGGCAATTATCTTTATTTTTGCTAGCTATGAAACCACCAGCAGCACACTTTCCTTTGTCTTGTATTCCCTGGCCACTCACCCTGACAGCCAGAAGAAACTGCAGGAGGAAATCGACAGGGCTCTACCCAATAAGGCACCTCCCACCTATGATACTGTGATGGAAATGGAGTATCTGGATATGGTGTTGAATGAAACCCCCAGATTGTATCCAATTGGTTATAGACTTGAGAGAGTCTGTAAAAAAGATATAAAACTTGATGGTGTGTTTATACCCAAAGGGTCAGTGGTGATGATTCCATTTTATACTCTTCAACATGACCCACAGCACTGGCCAGAGCCTGAGGAATTTCTTCCAGAAAGGTTCAGCAAGGAGAACAAGGGCAGCATTGATCCTTATGTATATCTGCCCTTTGGAAATGGACCCAGGAACTGTATTGGCATGAGGTTTGCTCTCATGAATATGAAACTTGCTCTCACTAAAGTTCTGCAAAACTTCTCCTTCCAGCTTTGTGAGGAAACACAGATACCTCTGAAATTAAGTAGACAAAGACTTTTTGGACCAGAAAAACCCATTGTTCTTAAGGTTGTGCCACGGGATGCAGTCATAACTGGAGCATGA--------------------->Rattus norvegicus Cyp3a9 NM 147206---ATGGATTTGATCCCAAACTTTTCCATGGAAACCTGGCTGCTCCTGGTTATCAGCCTGGTGCTCCTCTACCTATATGGAACTCATTCACATGGAATTTTTAAAAAGTTGGGAATTCCTGGGCCCAAACCTTTGCCTTTCTTGGGGACGATTCTTGCTTACAGGAAGGGCTTCTGGGAATTTGACAAATACTGCCATAAAAAATATGGGAAATTATGGGGGTTGTACGATGGTCGACAGCCTGTGCTAGCGATCACGGATCCAGACATAATCAAAACAGTGCTGGTGAAGGAATGTTACTCTACCTTCACAAACCGACGGAACTTTGGTCCAGTGGGTATTTTGAAAAAAGCCATCTCCATCTCTGAGGATGAAGAATGGAAGAGAATTCGAGCCCTGCTGTCTCCAACCTTCACCAGTGGGAAGCTCAAGGAGATGTTCCCCATCATTAACCAGTATACAGATATGTTGGTGAGAAACATGAGGCAGGGATCGGAGGAAGGCAAGCCCACCAGCATGAAAGACATCTTTGGGGCCTACAGCATGGATGTGATCACAGCCACCTCATTTGGAGTGAATGTTGATTCCCTTAACAACCCACAGGACCCTTTTGTGGAAAAAATCAAGAAGCTCTTAAAATTTGATATCTTTGATCCATTGTTCCTCTCAGTGACACTTTTTCCATTCCTTACCCCACTATTTGAAGCACTAAATGTCTCCATGTTTCCAAGAGATGTCATTGACTTTTTTAAAACTTCAGTAGAACGAATGAAAGAGAATCGCATGAAAGAGAAAGAAAAGCAAAGAATGGACTTTCTTCAGCTGATGATAAACTCCCAGAATTCCAAAGTCAAAGACTCTCATAAAGCATTATCCGATGTGGAGATTGTGGCCCAGTCAGTTATCTTCATTTTTGCCGGCTATGAGACCACTAGCAGTGCTCTTTCCTTTGTTTTGTATTTGCTGGCCATTCACCCTGATATACAGAAGAAACTGCAGGATGAAATTGATGCAGCTCTCCCCAATAAGGCACATGCCACCTATGATACCCTGCTACAAATGGAGTATCTAGATATGGTGGTGAATGAAACCCTCAGATTATATCCAATTGCTGGAAGGCTTGAGAGGGTCTGTAAGACAGATGTTGAAATCAATGGGGTGTTCATTCCCAAAGGGACTGTGGTGATGATACCAACCTTTGCTCTTCACAAAGACCCGCATTACTGGCCAGAGCCTGAGGAATTCCGCCCTGAAAGGTTCAGCAAGAAGAATCAGGATAACATCAATCCTTATATGTACCTGCCCTTTGGGAATGGACCCAGGAACTGTATTGGCATGAGGTTTGCTCTCATGAACATGAAAGTTGCTCTTGTCAGAGTCCTGCAGAACTTCTCCTTCCAACCTTGTAAGGAAACTCAGATCCCTTTAAAATTGAGCAAACAAGGACTTCTTCAACCAGAAAAGCCACTGCTTCTAAAAGTTGTGTCCAGAGATGAGACTGTGAATGGAGCTTGA--------------------->Sus scrofa CYP3A227 XM 021086162 LOC110259856---ATGGACCTGATTCCAAGTTTTTCTGTGGAAACTTGGCTTCTCCTGGTTACCAGCCTGGTGCTCCTCTATCTATATGGGACTTATTCACATGGATTTTTTAAGAAGCTGGGGATTCCTGGGCCGACACCTGTGCCTTATTTTGGAAATATTCTGGCCTACCGTAAGGGGATTTGGGATTTTGACAATAAATGTTTCCAAAAGTATGGAAAAATATGGGGGTTTTTTGATGGTCGGCAGCCCGTGTTGGCTATCACCGATCCAGACATGATCAAAACAGTCCTGGTGAAAGAATGTTACTCGGTCTTCACAAACCGGCGGACTTTTGGGCCAGTGGGAGTCATGAAAAATGCCATTTCTCTGGCTGAGGATGTGCAATGGAAGAAGATACGAACACTGCTGTGTCCAGCCTTCACCAGTGGAAAGCTCAAGGAAATATTCCCCATCATTGACCAATTTGGAGATGTCTTGGTGAAGAACATGAGGAGGGAAGCAGAGAAAGGCAAGCCTGTCACCATGAAAGACATTGTTGGGGGCTTCAGCATGGACGTGATCACTAGTACAGCTTTTGGAGTGAAGATTGATTCCCTCAACAACCCGCAAGACCCCTTTGTGTATTATACCAAGAGGCTCTTAAAATTTGATTTCCTCGACCCACTCCTTGTCATTTCCACACTCTTTCCATTCCTTCGTCCAATCTTTGAATTGTTAAGTTTCTCTGTGTTTCCAAAAACTGCTGTAAATTTTTTCACAAAATCTGTGAAAAAGATGAAAGAAAGTCGTCTCAAAGATGAAGATGTGCGTCGACTGGACCTTCTTCAGCTGATGATTAACTCCCAGAATTACAAAGAAATGGACGCCCGTAAAGCTCTGTCTGACCCAGAAATGGTGGCCCAAGGTATTATCTTTATTTTTGCTGGCTATGAGCCCACTACCGATTCTCTTTCCTTCCTTTTCTATAAACTGGCCACTCACCCTGATGTCCAGCGGAAGCTGCAGGAGGAGATTGATGCGACTTTTCCCAATAAGGCACCTCCCACATATGAGTCCCTGGAACAGATGGAGTATCTTGACATGGTGCTGAATGAATCTCTCAGATTGTTCCCACTTACGGCTAGACTGGAGAGGGTCTGCAAGAAGGATGTGGAAATCCATGGCGTGTTCATTCCCCAAGGGACCGTGGTGATGGTGCCGATCTACACGATTCATCAAGACCCAGGGCACTGGCCAGAGCCTGAGGAGTTCCATCCTGAAAGGTTCAGTAAGAAGAACAGGGACAGCATAAATCCTTACACACACATGCCCTTTGGCACTGGACCCCGCAACTGCATTGGCATGAGGTTTGCCCTCATGAACATGAAACTTGCTGCTGTCAAAGTCTTACAGAACTTCTCCTTCAAACCTTGCAAAGAAACACAGATTCCCCTGAAATTAAGTCCTCAAGTACTTATGCAACCAGAGAAACCCATTGTTCTAATGATTGAGCCACGAGATGGGACATTAAGCGGAGCTTGA--------------------->Sus scrofa CYP3A22 NM 001195509---ATGGACCTGATCCCAAGCTTTTCTGTGGAAACCTGGCTGCTTCTGGCTACCAGCCTGGTGCTCCTCTATCTATATGGGACCTATTCACACGGACTTTTTAAGAAGCTGGGGATTCCCGGGCCGAAACCTCTGCCTTATTTGGGAAGTGTTCTAGAATACCGTAAGGGCATGTGGCATTTTGACAATAAATGTTTTAAAAAGTATGGGAAAATGTGGGGGTTTTATGACGGTAGACAGCCTGTGCTGGCTATGAGAGACCCAGAAATGATCAAAGCAGTGCTTGTGAAAGAATGTTACTCGGCTTTCACAAATCGGCGGAATTTTGGTCCAGCGGGAGTTATGAAAAATGCCATCTCTGTGGCTAAGGATGAGCAATGGAAGCGAGTACGAACATTGCTGTCTCCAACCTTCACCAGTGGAAAGCTCAAAGAGATGTTCCCCATCATTGCCCAGTATGGAGATATGCTGGTGAGGAACCTGAGGAAGGAAGCAGAGAAAGGCAATTCCATCAACACGAAAGAAATCTTTGGGGCCTACAGCATGGACGTGATCACAAGCACAGCATTTGGAGTGGACGTCGATTCCCTCAACAACCCACGAGACCCCTTTGTACAATATGCCAGGAAGCTCCTAAGATTTGATTTCCTCGATCCATTTATTCTCTCAATAATATTATTTCCATTCCTCAGCCCATTCTTTGAAGTATTAGACATCACTTTGTTTCCAAGAAGTTCTGTGAAATTTTTCACACAGTCTGTAAAAAGGATGAAAGAAAGTCGCCTCAAAGATCAACAAATGCGCCGAGTGGACCTTCTTCAGCTGATGATTAACTCCCAGAATTCCAAAGAAACGGACGCCCATAAAGCTCTGTCTGATCAAGAACTTGTGGCCCAAAGTATTATCTTCATTTGTGCCGGCTATGAGACCACTAGCAGTTCTCTCTCCTTCCTGGCGTATATACTGGCTACTCACCCTGACGTCCAGCAGAATCTGCAGGAGGAGATTGATGCGACCTTCCCCAGCAAGGCCCTTCCCAGCTACGACGCCCTGGCACAGATGGAGTACCTTGACATGGTGGTGAATGAAATTCTCAGATTATACCCAATTGCTGCAAGACTGGAGAGGGTCTGTAAGAAGGATGTGGAAATCCATGGCGTGTCCGTTCCCAAGGGGACCGTGATGATGGTACCGGTTTTCTCGATTCACAGAGACCCGGAGCTCTGGCCGGAGCCTGAGGAGTTCCGGCCTGAAAGGTTCAGTAAGAAGAACAAGGACTCCATAAACCCTTACACTTACCTGCCCTTTGGGACTGGACCCCGCAACTGCATCGGCATGAGGTTTGCCCTCATGAACATGAAACTCGCTCTCGTCAGAGTCCTGCAGAACTTCTCCTTCAAACCTTGTAAAGAAACACAGACGCCCCTGAAACTAAGCTCTCAAGGACTTATACAACCAGAAAAACCCATTCTTCTGAAGGTTGTGCCCAGAGATGGGACCGTAAGTGGAGCCTGA--------------------->Sus scrofa CYP3A29 NM 214423---ATGGACCTGATCCCAGGCTTTTCCACAGAGACCTGGGTTCTCCTGGCTACCAGCCTGGTGCTCCTCTATCTATATGGGACGTATTCACATGGCCTTTTTAAGAAGCTGGGGATTCCTGGGCCAAGACCTCTGCCTTATTTTGGAAATATCCTGGGCTACCGTAAGGGTGTTGACCATTTTGACAAAAAGTGTTTTCAACAGTATGGGAAAATGTGGGGGGTTTATGATGGTCGGCAGCCTCTGCTGGCTGTCACAGATCCAAACATGATCAAATCAGTCCTAGTGAAGGAATGTTATTCTGTCTTCACAAACCGGAGGTCTTTTGGTCCATTGGGCGCTATGAGAAACGCTCTCTCTCTGGCTGAGGATGAAGAGTGGAAAAGAATCCGAACATTGCTGTCTCCGACCTTCACCAGTGGAAAGCTCAAGGAGATGTTCCCCATCATTAGCCATTATGGAGACTTGTTGGTGAGCAACCTGAGGAAGGAAGCAGAGAAAGGCAAACCTGTGACCATGAAAGACATCTTTGGGGCCTACAGCATGGACGTGATCACTAGCACAGCATTTGGAGTGAACATCGATTCCCTCAACAACCCACAAGACCCCTTTGTGGAAAACAGCAAGAAGCTCTTAAAATTTAGTTTCTTTGATCCATTCCTTCTCTCATTAATATTCTTTCCATTCCTCACCCCGATCTTCGAAGTATTAAACATCACTCTGTTTCCCAAAAGTTCTGTGAATTTTTTCACGAAATCTGTGAAAAGGATGAAAGAAAGTCGCCTCACAGATCAACAAAAGCGCCGAGTGGACCTTCTTCAGCTGATGATTAACTCCCAGAATTCCAAAGAAATGGACCCCCATAAAAGTCTGTCCAATGAAGAACTTGTGGCCCAAGGTATTATTTTTATTTTTGCTGGCTACGAGACCACTAGCAGTGCTCTCTCCCTCCTTGCGTATGAATTGGCCACTCACCCTGATGTCCAGCAGAAGCTGCAGGAGGAGATTGAGGCAACTTTCCCCAATAAGGCACCTCCCACCTACGATGCCCTGGCACAGATGGAGTATCTTGACATGGTAGTGAATGAAACTCTTAGATTATACCCAATTGCTGCTAGACTTGAGAGGGCCTGTAAGAAGGATGTGGAAATCCATGGCGTGTTCGTTCCCAAAGGGACCGTGGTGGTGGTGCCAGTCTTCGTGCTTCACAGAGACCCGGACCTCTGGCCAGAGCCTGAGGAGTTCCGTCCTGAAAGGTTCAGTAAGAAGCACAAGGACACCATAAATCCTTACACTTACCTGCCCTTTGGGACTGGACCCCGCAACTGCATTGGCATGAGGTTTGCCCTCATGAACATGAAACTCGCTCTCGTCAGAGTCCTGCAGAACTTCTCCTTCAAACCTTGTAAAGAAACACAGATCCCCCTGAAATTAACCACGCAAGGGCTTACACAACCGGAAAAACCTGTCGTTTTAAAGATTTTGCCCAGAGATGGGACCGTAAGTGGAGCCTGA--------------------->Sus scrofa CYP3A46 NM 001134824---ATGGACCTGATCCCAGGCTTTTCCACAGAAACCTGGGTTCTCCTGGCTACCAGCCTGGTGCTCCTCTATCTATATGGGACTTATTCACATGGCCTTTTTAAGAAGCTGGGGATTCCTGGGCCAAGACCTCTGCCTTATTTTGGAAATATCCTGGGCTACCGTAAGGGTGTTGACCATTTTGACAAAAAGTGTTTTCAACAGTATGGGAAAATGTGGGGGTTTTTTGATGGTCGGCAGCCCGTGTTGGCTATCACCGATCCAGACATGATCAAAACGGTCCTCGTGAAAGAATGTTATTCTGTCTTCACAAACCGGCGGTCTTTTGGTCCACGTGGTGCTATGAGAAGTGCTGTCTCTCTGGCTGAGGATGAAGAGTGGAAAAGAATCCGAACGTTGCTGTCTCCGACCTTCACCAGTGGAAAGCTCAAGGAGATGTTCCCCATCATTAGCCATTATGGAGACTTGTTGGTGAGCAACCTGAGGAAGGAAGCAGAGAAAGGCAAACCTGTGACCATGAAAGACATCTTTGGGGCCTACAGCATGGACGTGATCACTAGCACAGCATTTGGAGTGAACGTCGATTCCCTCAACAACCCACAAGACCCCTTTGTGGAAAACAGCAGGAAGCTCTTAAAATTTAGTTTCTTCAGTCCATTCTTTCTCTCAATAATATTCTTTCCATTCTTGACCCCGATCTTGGAAGTATTAAACATCACTCTGTTTCCCAAAAGTGTTGTGAATTTTTTCACGAGATCCATAAAAAGGATGAAAGAAAGTCGCCTCAAAGATACACAAAAGCACCGAGTGGACCTTCTTCAGCTGATGATTAACTCCCAGAATTCCAAAGAAATGGACGCCCATAAAGGTCTGTCCAATGAAGAACTTGTGGCCCAAGGTGTTATTTTTATTTTTGCTGGGTATGAGACCACTAGCAGTTCTCTCTCCCTCCTTGTGTATGAATTGGCCACTCACCCTGACGTCCAGCAGAAGCTGCAGGAGGAGATTGATGCGACCTTCCCCAATAAGGCGCCTCCCACCTACGATGGTCTGGCGCAAATGGAGTATCTTGACATGGTGGTGAACGAATCTCTCAGAATATTCCCAGTTACTCCTAGACTTGAGAGGTCCTGTAAGAAGGATGTGGAAATCCATGGCGTGTTCGTTCCCAAAGGGACCGTGATGATGGTGCCAATCTTTGCGCTTCACAGAGCCCCGGAGCTCTGGCCAGAGCCTGAGGAGTTCCGCCCTGAAAGGTTCAGTAAGAAGAACAAGGACACCATAAATCCTTACACTTACCTGCCCTTTGGGACTGGACCCCGCAACTGCATTGGCATGAGGTTTGCCCTCATGAACATGAAACTCGCTCTCGTCAGAGTCCTGCAGAACTTCTCCTTCAAACCTTGTAGAGAAACACAGATCCCCCTGAAAATAAGCTCCCAGGGACTTATCCAACCAGAAAAACCTATTGTTCTAATGGTTGTGCCCAGAGATGGGACCACAAGTGGAGCCTGA--------------------->Trichechus manatus latirostris CYP3A12 XM 012558254 LOC101340586---ATGGACCTGATCCCAAGCTTTTCCACCGAGACCTGGATTTTTCTGGTCACCAGCCTAGTGCTCCTCTACCTATATGGAACTTATTCACATAATGTTCTAAAGAAGCTCAGGATTCCTGGGCCTAAGCCTCTGCCTTTTGTGGGAAGTATTCTGGCCTACCGAAAGGGTGTATGGGATTTTGACATGAAATGTTCTAAAAAGTATGGAAAATTGTGGGGGTTTTATGATGGTCAATTGCCTGTAATAGCCATCACAGATCCAGGCATGATCAAGACAGTACTGGTGAAAGAATGTTACTCCAACTTTACAAACAGGAGGATTATTGGTCCATTGGGATTTATGAAATCTGCCGTTTCCTTGTCCAAGGATGAAGAATGGAAGAGAATACGAACATTGCTGTCTCCATCCTTCACTAGTGGAAAACTCAAGGAGATGTTCTGCATCATCGGCCAGTACGGAGACATGTTAGTGAAGCATCTGAGAGAGGAAGCACAGAAAGGCAAGCCAGTCACATTGAAAAGCATCTTCGGGGCTTATAGCATGGATGTGATCACTAGCACGTCATTTGGAGTGAACATCGATTCCCTCAACAACCCACAAGATCCCCTTCTGCAAAACGTCAGGAAGCTCTTAAAATTTGATTTTTTCGACCCATTGCTTTTCACAACAACACTCTTTCCATTCCTTACTTCAGTTTGTGAAGCATTAAGTATTTCTCTTTTTCCAAGAGCTGCTACTAATTTTGTCAAAAAATCTGTACAAAGAATGAAAGAAAGCCGCCTTAAAGATAATCAAAAG------GTAAGTTCTGGCGAAGTCTTAATTAATAGTAGGTTACCCCAAAAAACCGAAACCCACAGAACTTTGACTGATATGGAGCTTGTAGCCCAATCAATTATCTTTATTTCTGCTGGCTATGAGTCCACTAGCACTGCTCTTTCCTTCCTTATGTATTTAATGGCCACCCACCCTGATATTCAGCAGAAACTGCAGAAAGAGATTGATGCAACTTTTCCTAATAAGGCAACTCCCACATATGATGCTGTGTTACAGATGGAATATCTTGACATGGTGGTGAAAGAAACACTCAGATTATACCCAATCACTGCCAGACTTGAGAGGATCTGTAAAAATGATGCTGAAATCAATGGAGTGATCATTCCCAAAGGGACATTGGTGATGATACCAATCTTTGCTCTTCAACGAGACCCAGAACACTGGCCAGAGCCTGAGAAGTTCCTCCCTGAAAGATTCAGTAGGGAGAACAAGGACAATGTCGATCCTTACTTATATATGCCCTTTGGAAGTGGACCCCGAAACTGCATCGGCATGAGGTTTGCACTGATGAACATGAAACTTGCTGTCACCAGAATTCTGCAGGAGTTCTCCATCAAACCTTGTAAGGAAACACAGATCCCCTTAAAATTAGGCAGGAAACTAATTATCGCACCGGAAGGACCCATTGTGGTAATGTTTGAGTCAAGAGTTGGAAATGTAACTGGAGCCTGA--------------------->Trichechus manatus latirostris CYP3A8 XM 004385954 LOC101349229ATGATGGACCTCATTCCAAGCTTTTCCACAGAGACCTGGATTCTCCTGGTCACCAGTCTGGTGCTCCTCTACCTATATGGAACCTATTCACATAATGTTCTAAAGAAACTCCAGATTCCTGGGCCCAAGCCTCTGCCCTTTATGGGAAATCTTCTGGCCTACCGCAAGGGTTGGTGGAAATTTGACATGAAATGTTATAAAAAGTATGGAAAAATATGGGGGTTTTATGATGGTCGACAGCCTGTGATAGCCATAACAGATCCAGACATGATCAAGACAGTACTAGTGAAAGAATGTTATTCCACCTTCACAAACCGGAGGGTTTTGGGCCCAATGGGATTTATGAAATCTGCCATCTCTGTGTCCAGCGATGAAGAGTGGAAGAGAATACGAACATTGTTGTCTCCAACTTTCACCAGTGGAAAACTCAAGGAGATGTTCCCCATTATCAGACAGTATGGAGACGTGGTAGTGAAACATCTGAGAGAGGAAGTACAGAAAGGCAAGCCTGTCACATTGAAAAGCATCTTCGGGGCCTACAGCATGGATGTGACCACTAGCACATCATTTGGAGTGAACATTGATTCCCTCAGCAACCCACAAGATCCCTTTGTTGAAAACGTCAGGAAGCTCTTAAAATTAGATATCTTCGACCCATTCATTTTCTTAATAGTACTCTTTCCATTCCTTACTCCAATTTGTGAAGCATTAAGTATCTCTGTGTTTCCAAGAGCTGTTACTGATTTTTTCATAAAATCTGTAAAGACAATAAAAGAAAGCCGCCTTAAAGATAATAAAAAGCACCGAGTGGATTTTCTTCAGCTGATGATTGACTCTCAGAATTCCAAAGAAACTGCATCCCATAAAGCTTTGTCTGATATGGAGCTCATAGCCCAATCAATTGTCTTTATTTTTGCTGGCTATGAGACCACGAGTACTGCTCTTTCCTTCCTTATGTATTTATTGGCCACCCACCCTGATATTCAGCAGAAACTGCAGAAGGAGATTGATGCAGCTTTGCCCAATAAGGCACCTCCCACATATGATGTCATGTTACAGATGGAATATCTTGACATGGTGGTGAATGAAACACTCAGAATATTCCCAATTATTGGCAGACTTGAGAGGGTCTGTAAGAAAGATGTTGAAATCAACGGAGTGATCATTCCAAAAGGGGCATTGGTAATTATACCAGTCTTTGTTCTTCACCGAGACCCAGAACTCTGGCCAGAGCCTGAGAAGTTCCTTCCTGAAAGGTTCAGTAAGGAGAACAAGGACAATATAGATCCTTACGTATACCTGCCCTTTGGAACTGGACCCCGAAATTGCATTGGCATGAGGTTTGCTCTCATGAACATGAAACTTGCTGTCATCAGAGTTCTTCAGGAGTTCTCCATCAAACCTTGTAAAGAAACACAGATCCCCTTAAAATTCGGCAAGGGAGGATTTCTTGCACCGGAAGAACCTGTTGTTATGATGTTTGAATCAAGAGATGGAAATGCAAGTGAAGCCTGA---------------------
