# Supplementary material for: A Comparative Genomic and Phylogenetic Investigation of the Xenobiotic Metabolism Enzymes of Cytochrome P450 in Elephants Shows Loss in CYP2E and CYP4A
Source: Animals (Basel). 2023 Jun 9;13(12):1939. doi: 10.3390/ani13121939 (PMC10294912; doi:10.3390/ani13121939)
Supplement: Supplementary file 1 [file animals-13-01939-s001.zip › Figure S1. Cofirmation for pseudonization of CYP4A.pdf]

Confirmation of pseudonization in CYP4As gene

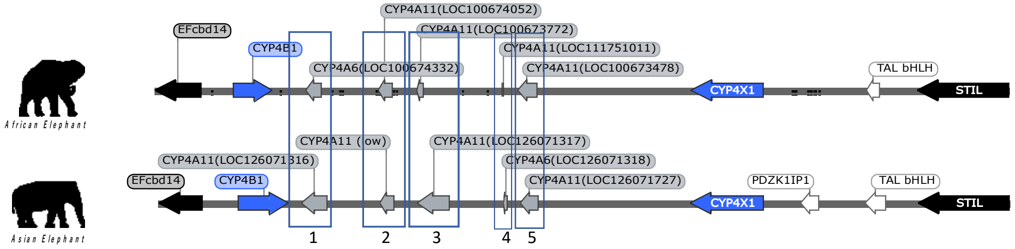

Group 1

number of aligned nucleotide	313	314	315	316	317	318	319	320	321	322	323	324	325	326	327	328	329	330	331	332	333	334	335	336	337	338	339	340	341	342	343	344	345	346	347	348	349	350	351	352	353	354	355	356	357	358	359	360	361	362	363	364	365	366	367	368	369
translated to animo acid Human CYP4A11	M			K			V			I			L			G			R			S			D			P			K			S			H			G			S			Y			R			F			L		
H. sapiens CYP4A11 NM 000778	A	T	G	A	A	G	G	T	G	A	T	T	C	T	G	G	G	G	A	G	A	T	C	A	G	A	C	C	C	G	A	A	A	T	C	C	C	A	T	G	G	T	T	C	C	T	A	C	A	G	A	T	T	C	C	T	G
H. sapiens CYP4A22 NM 001010969 XM 208213	A	T	G	A	A	G	G	T	G	A	T	T	C	T	G	G	G	G	A	G	A	T	C	A	G	A	C	C	C	G	A	A	A	T	C	C	C	A	T	G	G	A	T	C	C	T	A	C	A	A	A	T	T	C	C	T	G
O. afer afer XM 007946703 LOC103201934	A	T	G	A	A	G	G	T	G	A	T	T	C	T	A	G	G	G	A	G	A	T	C	A	G	A	C	C	C	A	A	A	G	T	T	A	A	G	A	G	A	T	A	C	C	T	A	C	A	G	A	T	T	C	A	T	G
E. edwardii XM 006902916 LOC102867299	A	T	T	A	A	G	G	T	G	A	T	T	C	T	G	G	G	G	A	G	A	T	C	A	G	A	C	C	C	A	A	A	G	T	C	T	A	C	T	A	G	A	A	C	C	T	A	C	A	G	A	T	T	C	C	T	G
E. edwardii XM 006902913 LOC102862835	A	T	G	A	A	G	G	T	G	G	T	T	C	T	G	G	C	G	A	G	A	T	C	A	G	A	T	G	T	A	A	A	G	A	C	T	C	A	T	G	A	G	C	G	C	T	A	C	C	G	A	T	T	C	C	T	A
T. manatus latirostris XM 004371774 LOC101346203	G	C	G	A	A	G	G	T	G	A	T	T	C	T	G	G	G	G	A	G	A	C	C	A	G	A	C	C	C	A	A	A	A	T	C	T	C	A	T	G	A	G	C	C	C	T	A	C	A	G	A	T	T	C	C	T	G
T. manatus latirostris XM 012554256 LOC101345950	A	T	G	A	A	G	G	T	G	A	T	T	A	T	G	G	G	G	A	G	A	T	C	A	G	A	C	C	C	A	G	A	G	G	C	T	A	A	T	G	A	T	G	C	T	T	A	T	A	G	A	T	T	C	C	T	G
L. Africana CYP4A11 LOC100673772 NW 003573437	T	G	A	A	G	G	T	G	G	T	T	A	T	G	G	A	G	A	G	A	T	C	A	G	A	C	A	C	A	A	A	G	G	C	T	A	A	C	G	A	T	G	C	C	T	A	C	G	G	A	T	T	C	C	T	A	G
E. maximus CYP4A11 LOC126071317 NC 064821	T	G	A	A	G	G	T	G	G	T	T	A	T	G	G	A	G	A	G	A	T	C	A	G	A	C	A	C	A	A	A	G	G	C	T	A	A	C	G	A	T	G	C	C	T	A	C	G	G	A	T	T	C	C	T	A	G
translated to animo acid E.maximus CYP4A11	*			R			W			L			W			R			D			Q			T			Q			R			L			T			M			P			T			D			S			*		
	EXON2																									EXON3																															

Group 2

number of aligned nucleotide	79	80	81	82	83	84	85	86	87	88	89	90	91	92	93	94	95	96	97	98	99	100	101	102	103	104	105	106	107	108	109	110	111	112	113	114	115	116	117	118	119	120	121	122	123	124	125	126	127	128	129	130	131	132	133	134	135	136	137	138	139	140	141
translated to animo acid Human CYP4A11	L			L			L			L			L			I			K			A			V			Q			L			Y			L			H			R			Q			W			L			L			K			A		
H. sapiens CYP4A11 NM 000778	C	T	G	C	T	T	C	T	G	C	T	G	C	T	G	A	T	C	A	A	G	G	C	A	G	T	T	C	A	G	C	T	C	T	A	C	C	T	G	C	A	C	A	G	G	C	A	G	T	G	G	C	T	G	C	T	C	A	A	A	G	C	C
H. sapiens CYP4A22 NM 001010969 XM 208213	C	T	G	C	T	T	C	T	G	C	T	G	C	T	G	A	T	C	A	A	G	G	C	A	G	C	T	C	A	G	C	T	C	T	A	C	C	T	G	C	A	T	A	G	G	C	A	G	T	G	G	C	T	G	C	T	C	A	A	A	G	C	C
O. afer afer XM 007946703 LOC103201934	C	T	G	G	T	T	C	T	G	C	T	G	T	T	G	C	T	C	A	A	G	G	C	A	G	C	C	C	A	G	T	T	C	T	A	C	T	T	G	C	G	C	A	G	G	C	A	A	T	G	G	C	T	A	C	T	C	A	A	A	G	C	C
E. edwardii XM 006902916 LOC102867299	C	T	T	G	T	T	C	T	G	C	T	G	C	T	G	T	T	C	A	A	G	G	C	A	A	T	C	C	A	A	T	T	A	T	A	C	C	T	G	C	G	C	A	G	A	C	A	G	T	G	G	C	T	G	T	T	G	A	A	A	G	T	C
E. edwardii XM 006902913 LOC102862835	C	T	A	G	T	T	C	T	A	C	T	G	C	T	G	C	T	C	A	A	G	G	C	A	G	C	T	C	A	G	T	T	C	T	A	C	C	T	G	G	A	C	A	G	G	C	A	G	T	G	G	C	T	A	C	T	C	A	A	A	G	C	T
T. manatus latirostris XM 004371774 LOC101346203	C	T	G	G	T	T	C	T	G	C	T	G	C	T	G	C	T	C	A	A	G	G	C	A	G	C	C	C	A	G	C	T	C	T	A	C	C	T	G	C	G	C	A	G	G	C	A	G	T	G	G	C	T	A	C	T	C	A	A	G	G	C	C
T. manatus latirostris XM 012554256 LOC101345950	C	T	G	G	T	T	C	T	G	C	T	G	C	T	G	C	T	C	A	A	A	G	C	A	G	C	C	C	A	G	C	T	C	T	A	C	C	T	G	T	G	C	A	G	G	C	T	A	T	G	G	C	T	A	C	T	C	A	A	A	G	C	C
L. Africana CYP4A11 LOC100673772 NW 003573437	C	T	G	G	C	T	C	T	G	C	T	A	c	T	T	G	C	T	C	A	A	G	G	C	A	G	C	C	C	A	G	C	T	C	T	A	T	C	T	G	C	G	C	A	G	G	C	G	T	T	G	G	T	T	A	C	T	C	A	A	A	G	C
E. maximus CYP4A11 LOC126071317 NC 064821	C	T	G	G	C	T	C	T	G	C	T	A	T	T	G	C	T	C	A	A	G	G	C	A	G	C	C	C	A	G	C	T	C	T	A	T	C	T	G	C	G	C	A	G	G	C	G	T	T	G	G	T	T	A	C	T	C	A	A	A	G	C	C
translated to animo acid E.maximus CYP4A11	L			A			L			L			L			L			K			A			A			Q			L			Y			L			R			R			R			W			L			L			K			A		
	EXON2																																																														

number of aligned nucleotide	289	290	291	292	293	294	295	296	297	298	299	300	301	302	303	304	305	306	307	308	309	310	311	312	313	314	315	316	317	318	319	320	321	322	323	324	325	326	327	328	329	330	331	332	333	334	335	336	337	338	339	340	341	342	343
translated to animo acid Human CYP4A11	V			Q			L			Y			D			P			D			Y			M			K			V			I			L			G			R			S			D			P			
H. sapiens CYP4A11 NM 000778	G	T	C	C	A	G	C	T	C	T	A	T	G	A	C	C	C	T	G	A	C	T	A	T	A	T	G	A	A	G	G	T	G	A	T	T	C	T	G	G	G	G	A	G	A	T	C	A	G	A	C	C	C	G	-
H. sapiens CYP4A22 NM 001010969 XM 208213	G	T	C	C	A	G	C	T	C	T	A	T	G	A	C	C	C	T	G	A	C	T	A	T	A	T	G	A	A	G	G	T	G	A	T	T	C	T	G	G	G	G	A	G	A	T	C	A	G	A	C	C	C	G	-
O. afer afer XM 007946703 LOC103201934	T	T	C	C	A	T	G	T	T	T	A	T	G	A	T	C	C	T	G	A	C	T	A	C	A	T	G	A	A	G	G	T	G	A	T	T	C	T	A	G	G	G	A	G	A	T	C	A	G	A	C	C	C	A	-
E. edwardii XM 006902916 LOC102867299	G	T	C	A	T	G	A	T	C	T	A	T	G	A	T	C	C	T	G	A	C	T	A	C	A	T	T	A	A	G	G	T	G	A	T	T	C	T	G	G	G	G	A	G	A	T	C	A	G	A	C	C	C	A	-
E. edwardii XM 006902913 LOC102862835	A	T	C	T	C	A	A	T	A	T	A	T	G	A	T	C	C	T	G	A	C	T	A	C	A	T	G	A	A	G	G	T	G	G	T	T	C	T	G	G	C	G	A	G	A	T	C	A	G	A	T	G	T	A	-
T. manatus latirostris XM 004371774 LOC101346203	T	T	C	C	A	G	G	T	C	T	A	T	G	A	T	C	C	C	G	A	C	T	A	C	G	C	G	A	A	G	G	T	G	A	T	T	C	T	G	G	G	G	A	G	A	C	C	A	G	A	C	C	C	A	-
T. manatus latirostris XM 012554256 LOC101345950	A	T	C	C	T	G	G	T	C	A	A	T	G	A	C	C	C	T	G	A	C	T	A	C	A	T	G	A	A	G	G	T	G	A	T	T	A	T	G	G	G	G	A	G	A	T	C	A	G	A	C	C	C	A	-
L. Africana CYP4A11 LOC100673772 NW 003573437	C	T	A	C	G	C	T	G	A	C	T	A	G	A	T	G	A	A	G	G	T	G	A	T	T	C	T	G	G	G	G	A	G	A	T	C	A	G	A	C	C	C	A	A	A	G	C	T	T	C	A	T	G	A	T
E. maximus CYP4A11 LOC126071317 NC 064821	A	T	G	G	C	T	G	T	C	T	A	C	-	-	-	G	C	T	G	A	C	T	A	G	A	T	G	A	A	G	G	T	G	A	T	T	C	T	G	G	G	G	A	G	A	T	C	A	G	A	C	C	C	A	-
translated to animo acid E.maximus CYP4A11	M			A			V			Y						A			D			*			M			K			V			I			L			G			R			S			D			P			
	EXON2																																																							

Group 3

number of aligned nucleotide	196	197	198	199	200	201	202	203	204	205	206	207	208	209	210	211	212	213	214	215	216	217	218	219	220	221	222	223	224	225	226	227	228	229	230	231	232	233	234	235	236	237	238	239	240	241	242	243	244	245	246	247	248	249	250	251	252
translated to animo acid Human CYP4A11	L			Q			Q			D			Q			E			L			Q			R			I			Q			K			W			V			E			T			F			P			S		
H. sapiens CYP4A11 NM 000778	C	T	C	C	A	A	C	A	G	G	A	C	C	A	G	G	A	G	C	T	A	C	A	A	C	G	G	A	T	T	C	A	G	A	A	A	T	G	G	G	T	G	G	A	G	A	C	A	T	T	C	C	C	A	A	G	T
H. sapiens CYP4A22 NM 001010969 XM 208213	T	T	C	C	A	A	C	A	C	G	A	C	C	A	G	G	A	G	C	T	A	C	A	A	C	G	G	A	T	T	C	A	G	G	A	A	C	G	G	G	T	G	A	A	G	A	C	A	T	T	C	C	C	A	A	G	T
O. afer afer XM 007946703 LOC103201934	T	T	T	C	A	A	A	G	G	G	A	C	A	C	A	G	A	G	C	T	G	C	A	A	C	A	T	T	T	C	T	T	G	A	C	A	T	G	G	G	T	T	C	A	G	A	A	A	T	T	C	C	C	A	T	G	T
E. edwardii XM 006902916 LOC102867299	T	T	T	A	C	A	A	A	G	G	A	T	C	A	G	G	A	G	T	T	G	C	A	A	C	A	G	T	T	A	C	T	G	A	A	A	T	G	G	G	T	G	G	A	G	A	A	A	T	T	C	C	C	A	T	G	T
E. edwardii XM 006902913 LOC102862835	T	T	T	C	A	A	A	C	A	G	A	T	C	A	G	G	A	G	C	T	G	C	C	A	C	A	G	T	T	G	A	T	G	A	A	A	T	G	G	G	T	G	G	A	G	A	A	A	T	T	C	C	C	A	T	G	T
T. manatus latirostris XM 004371774 LOC101346203	T	T	T	C	A	A	A	A	G	G	A	T	C	A	G	G	A	G	C	T	G	C	A	A	C	A	T	T	T	T	C	T	G	A	A	A	T	G	G	G	T	A	G	A	G	A	A	A	T	T	C	C	C	G	T	G	T
T. manatus latirostris XM 012554256 LOC101345950	T	T	T	C	A	A	A	A	G	G	A	T	C	A	G	G	A	G	C	T	G	C	A	A	C	A	G	T	T	G	A	T	G	A	A	A	T	G	G	G	T	A	G	A	G	A	A	A	T	T	C	C	C	A	T	G	T
L. Africana CYP4A11 LOC100673772 NW 003573437	-	-	-	-	-	-	-	-	-	-	-	-	-	-	-	G	A	G	A	T	C	A	G	A	C	T	C	A	A	A	C	T	C	T	C	A	T	G	G	T	A	G	C	T	A	C	A	G	A	T	T	C	C	T	-	-	-
E. maximus CYP4A11 LOC126071317 NC 064821	T	T	C	C	A	A	A	A	A	G	A	A	C	A	G	G	A	G	C	T	G	C	A	A	C	A	G	C	T	T	C	T	G	A	A	A	T	G	G	T	A	G	G	T	A	A	A	T	T	C	C	A	T	G	T	G	C
translated to animo acid E.maximus CYP4A11	F			Q			K			E			Q			E			L			Q			Q			L			L			K			W			*			V			N			S			M			C		
																																							EXON2																	

Group 4

number of aligned nucleotide	340	341	342	343	344	345	346	347	348	349	350	351	352	353	354	355	356	357	358	359	360	361	362	363	364	365	366	367	368	369	370	371	372	373	374	375	376	377	378	379	380	381	382	383	384	385	386	387	388	389	390	391	392	393	394	395	396
translated to animo acid Human CYP4A11	Q			H			T			D			Q			V			I			Q			L			R			K			A			Q			L			Q			K			E			G			E		
H. sapiens CYP4A11 NM 000778	C	A	G	C	A	C	A	C	A	G	A	C	C	A	A	G	T	G	A	T	C	C	A	A	C	T	G	A	G	G	A	A	G	G	C	T	C	A	A	C	T	A	C	A	G	A	A	G	G	A	G	G	G	G	G	A	G
H. sapiens CYP4A22 NM 001010969 XM 208213	C	A	G	C	A	C	A	C	A	G	A	C	C	A	A	G	T	G	A	T	C	C	A	A	C	T	G	A	G	G	A	A	G	G	C	T	C	A	A	C	T	A	C	A	G	A	A	G	G	A	G	G	G	G	G	A	G
O. afer afer XM 007946703 LOC103201934	C	A	A	C	A	C	A	C	A	G	A	C	A	A	A	G	T	G	A	T	T	A	A	G	C	T	G	A	G	A	A	A	A	G	C	T	A	G	T	C	T	G	C	A	A	G	A	G	G	A	G	G	G	A	G	A	G
E. edwardii XM 006902916 LOC102867299	C	A	G	C	A	C	A	C	A	G	A	C	C	A	A	G	T	G	A	T	C	A	A	G	C	A	A	A	G	G	A	A	G	A	T	T	T	C	T	C	T	G	C	A	G	A	A	G	G	A	A	G	G	A	G	A	G
E. edwardii XM 006902913 LOC102862835	C	A	A	C	A	C	A	C	A	G	A	C	C	A	A	G	T	G	A	T	C	A	A	A	C	T	G	A	G	G	A	A	G	G	C	T	C	A	A	C	T	T	C	A	A	G	A	G	A	A	T	G	G	A	G	G	G
T. manatus latirostris XM 004371774 LOC101346203	C	A	G	C	A	C	A	C	A	G	A	G	C	A	A	G	T	G	A	T	C	A	A	G	C	T	G	A	G	G	A	A	G	G	C	T	G	A	T	C	T	G	C	A	G	A	A	G	G	A	A	G	G	A	G	A	G
T. manatus latirostris XM 012554256 LOC101345950	C	A	G	C	A	T	A	C	A	G	A	C	C	A	A	G	T	C	A	T	C	A	G	G	T	T	G	A	G	G	A	A	G	G	C	T	A	G	C	C	T	G	C	A	G	G	A	G	?	G	A	G	G	A	G	A	G
L. Africana CYP4A11 LOC100673772 NW 003573437	C	A	G	C	A	C	A	C	A	G	A	C	T	G	A	G	T	G	A	T	C	A	A	G	T	G	G	A	G	A	A	A	A	G	C	T	T	A	C	C	T	A	A	A	G	G	A	G	G	A	A	G	G	A	G	A	A
E. maximus CYP4A11 LOC126071317 NC 064821	C	A	G	C	A	C	A	C	A	G	A	C	T	G	A	G	T	G	A	T	C	A	A	G	T	G	G	A	G	A	A	A	A	G	C	T	T	A	C	T	T	A	A	A	G	G	A	G	G	A	A	G	G	A	G	A	A
translated to animo acid E.maximus CYP4A11	Q			H			T			D			*			V			I			K			W			R			K			A			Y			L			K			E			E			G			E		
							EXON 6																																								EXON 7											

Group 5

number of aligned nucleotide	313	314	315	316	317	318	319	320	321	322	323	324	325	326	327	328	329	330	331	332	333	334	335	336	337	338	339	340	341	342	343	344	345	346	347	348	349	350	351	352	353	354	355	356	357	358	359	360	361	362	363	364	365	366	367	368	369
translated to animo acid Human CYP4A11	M			K			V			I			L			G			R			S			D			P			K			S			H			G			S			Y			R			F			L		
H. sapiens CYP4A11 NM 000778	A	T	G	A	A	G	G	T	G	A	T	T	C	T	G	G	G	G	A	G	A	T	C	A	G	A	C	C	C	G	A	A	A	T	C	C	C	A	T	G	G	T	T	C	C	T	A	C	A	G	A	T	T	C	C	T	G
H. sapiens CYP4A22 NM 001010969 XM 208213	A	T	G	A	A	G	G	T	G	A	T	T	C	T	G	G	G	G	A	G	A	T	C	A	G	A	C	C	C	G	A	A	A	T	C	C	C	A	T	G	G	A	T	C	C	T	A	C	A	A	A	T	T	C	C	T	G
O. afer afer XM 007946703 LOC103201934	A	T	G	A	A	G	G	T	G	A	T	T	C	T	A	G	G	G	A	G	A	T	C	A	G	A	C	C	C	A	A	A	G	T	T	A	A	G	A	G	A	T	A	C	C	T	A	C	A	G	A	T	T	C	A	T	G
E. edwardii XM 006902916 LOC102867299	A	T	T	A	A	G	G	T	G	A	T	T	C	T	G	G	G	G	A	G	A	T	C	A	G	A	C	C	C	A	A	A	G	T	C	T	A	C	T	A	G	A	A	C	C	T	A	C	A	G	A	T	T	C	C	T	G
E. edwardii XM 006902913 LOC102862835	A	T	G	A	A	G	G	T	G	G	T	T	C	T	G	G	C	G	A	G	A	T	C	A	G	A	T	G	T	A	A	A	G	A	C	T	C	A	T	G	A	G	C	G	C	T	A	C	C	G	A	T	T	C	C	T	A
T. manatus latirostris XM 004371774 LOC101346203	G	C	G	A	A	G	G	T	G	A	T	T	C	T	G	G	G	G	A	G	A	C	C	A	G	A	C	C	C	A	A	A	A	T	C	T	C	A	T	G	A	G	C	C	C	T	A	C	A	G	A	T	T	C	C	T	G
T. manatus latirostris XM 012554256 LOC101345950	A	T	G	A	A	G	G	T	G	A	T	T	A	T	G	G	G	G	A	G	A	T	C	A	G	A	C	C	C	A	G	A	G	G	C	T	A	A	T	G	A	T	G	C	T	T	A	T	A	G	A	T	T	C	C	T	G
L. Africana CYP4A11 LOC100673772 NW 003573437	T	G	A	A	G	G	T	G	A	T	T	A	T	G	G	G	G	A	-	-	-	-	-	-	-	-	-	-	-	-	-	-	-	-	-	-	G	A	T	C	A	G	A	C	C	C	A	A	A	G	G	C	T	A	T	T	G
E. maximus CYP4A11 LOC126071317 NC 064821	T	G	A	A	G	G	T	G	A	T	T	A	T	G	G	G	G	A	-	-	-	-	-	-	-	-	-	-	-	-	-	-	-	-	-	-	G	A	T	C	A	G	A	C	C	C	A	A	A	G	G	C	T	A	T	T	G
translated to animo acid E.maximus CYP4A11	*			R			*			L			W			G																					D			Q			T			Q			R			L			L		
														EXON2																																					EXON3				

Figure S1. The loss of the CYP4A gene in African and Asian elephants was confirmed by MEGAX alignment. CYP4A gene sequences were visualized and compared to confirm pseudogene. Analysis was performed separately for each homologous gene as described in the synteny map. Human CYP4A11 (NM_000778) were referenced for nucleotide number of aligned genes. Different exon regions were described and highlighted in the manuscript. In addition, human and Asian elephant amino acids are listed at the top and bottom of the table. As a result, in all sequences stop codons (*) were found. Groups 1, 4, and 5 identified several stop codons in positions conserved in African and Asian elephants. Groups 2 and 3 are less similar in sequence, particularly in Group 2, a gene insertion was identified in African elephant. Stop codon detected in all group was located on the loci at less than 50% of the full length of the gene sequence.
