# Supplementary material for: A Comparative Genomic and Phylogenetic Investigation of the Xenobiotic Metabolism Enzymes of Cytochrome P450 in Elephants Shows Loss in CYP2E and CYP4A
Source: Animals (Basel). 2023 Jun 9;13(12):1939. doi: 10.3390/ani13121939 (PMC10294912; doi:10.3390/ani13121939)
Supplement: Supplementary file 1 [file animals-13-01939-s001.zip › Table S1. Assemble and annotation information.pdf]

	Assembly name	Organism Name	Annotation Name
1	GCF_000243295.1	*Trichechus manatus latirostris*	NCBI Annotation Release 102
2	GCF_000001405.40	*Homo sapiens*	GCF_000001405.40-RS_2023_03
3	GCF_000001905.1	*Loxodonta africana*	NCBI Annotation Release 102
4	GCF_024166365.1	*Elephas maximus*	GCF_024166365.1-RS_2023_02
5	GCF_000298275.1	*Orycteropus afer afer*	NCBI Annotation Release 101
6	GCF_002863925.1	*Equus caballus*	NCBI Annotation Release 103
7	GCF_002263795.2	*Bos taurus*	NCBI eukaryotic genome annotation pipeline
8	GCF_000299155.1	*Elephantulus edwardii*	NCBI Annotation Release 100
9	GCF_015227675.2	*Rattus norvegicus*	NCBI Annotation Release 108
10	GCF_000003025.6	*Sus scrofa*	NCBI Annotation Release 106
11	GCF_014441545.1	*Canis lupus familiaris*	NCBI Annotation Release 106
12	GCF_018350175.1	*Felis catus*	NCBI Annotation Release 105

Supplemental Table S1: Assembly and annotation names of sequences from 12 different species: African elephant (*Loxodonta Africana*) and Asian elephant (*Elephas maximus*), aardvark (Orycteropus afer), manatee (*Trichechus manatus*), cape elephant shrew (*Elephantulus edwardii*), horse (*Equus ferus caballus*), rat (*Rattus norvegicus*), dog (*Canis lupus familiaris*), cat (*Felis catus*), pig (*Sus scrofa domesticus*), cow (*Bos taurus*), and human (*Homo sapiens*).
